# The ToxCast pipeline: updates to curve-fitting approaches and database structure

**DOI:** 10.3389/ftox.2023.1275980

**Published:** 2023-09-21

**Authors:** M. Feshuk, L. Kolaczkowski, K. Dunham, S. E. Davidson-Fritz, K. E. Carstens, J. Brown, R. S. Judson, K. Paul Friedman

**Affiliations:** ^1^ Center for Computational Toxicology and Exposure, Office of Research and Development, U.S. Environmental Protection Agency, Research Triangle Park, Durham, NC, United States; ^2^ National Student Services Contractor, Oak Ridge Associated Universities, Oak Ridge, TN, United States

**Keywords:** ToxCast database, high-throughput screening, data analysis, data pipeline, new approach methods

## Abstract

**Introduction:** The US Environmental Protection Agency Toxicity Forecaster (ToxCast) program makes *in vitro* medium- and high-throughput screening assay data publicly available for prioritization and hazard characterization of thousands of chemicals. The assays employ a variety of technologies to evaluate the effects of chemical exposure on diverse biological targets, from distinct proteins to more complex cellular processes like mitochondrial toxicity, nuclear receptor signaling, immune responses, and developmental toxicity. The ToxCast data pipeline (tcpl) is an open-source R package that stores, manages, curve-fits, and visualizes ToxCast data and populates the linked MySQL Database, invitrodb.

**Methods:** Herein we describe major updates to tcpl and invitrodb to accommodate a new curve-fitting approach. The original tcpl curve-fitting models (constant, Hill, and gain-loss models) have been expanded to include Polynomial 1 (Linear), Polynomial 2 (Quadratic), Power, Exponential 2, Exponential 3, Exponential 4, and Exponential 5 based on BMDExpress and encoded by the R package dependency, tcplfit2. Inclusion of these models impacted invitrodb (beta version v4.0) and tcpl v3 in several ways: (1) long-format storage of generic modeling parameters to permit additional curve-fitting models; (2) updated logic for winning model selection; (3) continuous hit calling logic; and (4) removal of redundant endpoints as a result of bidirectional fitting.

**Results and discussion:** Overall, the hit call and potency estimates were largely consistent between invitrodb v3.5 and 4.0. Tcpl and invitrodb provide a standard for consistent and reproducible curve-fitting and data management for diverse, targeted *in vitro* assay data with readily available documentation, thus enabling sharing and use of these data in myriad toxicology applications. The software and database updates described herein promote comparability across multiple tiers of data within the US Environmental Protection Agency CompTox Blueprint.

## Highlights

• The ToxCast program makes targeted *in vitro* screening assay data publicly available for prioritization and hazard characterization.

• Data needs in next generation risk assessment necessitated software and database updates for consistent and reproducible curve-fitting and data management across screening efforts.

• Updates include additional models, bidirectional curve-fitting, and continuous hit calling.

• Annotation structure, fit categories, and cautionary flags on curve-fitting behavior were modified for future invitrodb release.

• Curve-fitting updates resulted in small changes in activity hit calls and potency estimates but without a uniform trend.

## Introduction

Thousands of chemicals found in commerce and the environment are associated with limited information regarding their potential impacts or hazards to human health and ecological systems (Judson et al., 2009; USEPA, 2021c). Given time and resource requirements of traditional toxicity testing, new approach methodologies (NAMs) could inform prioritization and assessment of chemicals of concern in an efficient, risk-based context (USEPA, 2011; Paul Friedman et al., 2020; USEPA, 2021a; USEPA, 2021b; USEPA, 2021c; USEPA, 2022a; Dobreniecki et al., 2022). Leading this charge, the CompTox Blueprint (Thomas et al., 2019) outlined a tiered testing framework for hazard characterization wherein Tier 1 considers both chemical structure and broad coverage, high content assays across multiple cell types to comprehensively evaluate a chemical or groups of chemicals based on structural similarity to others with potential hazards. Chemicals from Tier 1 with a predicted biological target or pathway could undergo further screening in Tier 2 targeted assays and Tier 3 organotypic or biologically complex models, such as assays within the US Environmental Protection Agency (EPA) Toxicity Forecaster (ToxCast) program and multi-lateral Tox21 program (Kavlock et al., 2012; Richard et al., 2021). The ToxCast program makes *in vitro* medium- and high-throughput screening assay data publicly available for the prioritization and hazard characterization of thousands of chemicals of interest. The assays included use a variety of technologies to evaluate the effects of chemical exposure on diverse biological targets from distinct proteins to more complex cellular processes like mitochondrial toxicity, nuclear receptor signaling, immune responses, and developmental toxicity. Importantly, public access to structured data analyzed in a common way provides data for next-generation risk assessment using bioactivity as an indicator of potential hazard (Thomas et al., 2019; Baltazar et al., 2020; Bhuller et al., 2021; Dent et al., 2021; Rajagopal et al., 2022).

**FIGURE 1:**
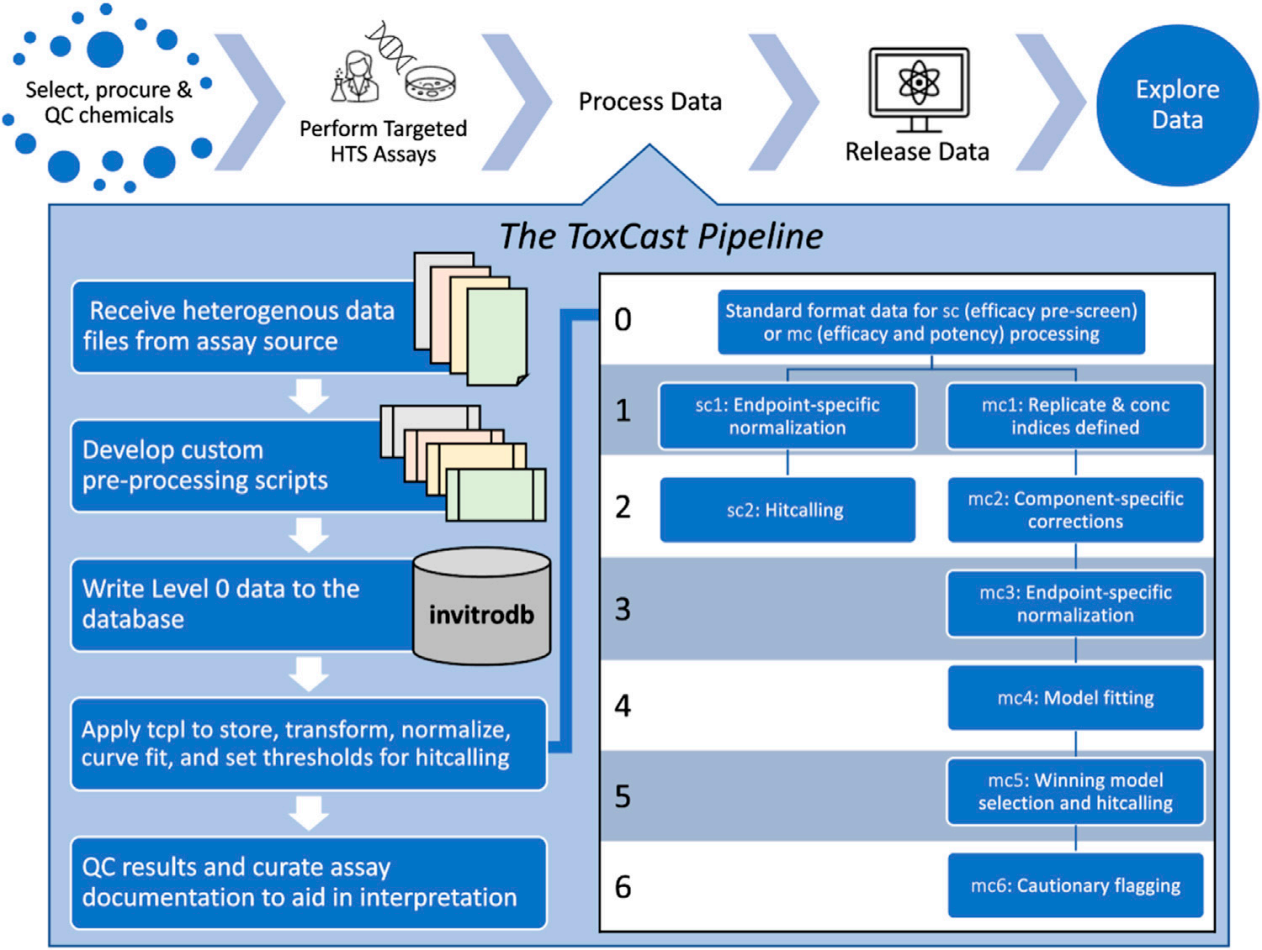
Conceptual overview of the ToxCast Pipeline functionality. The ToxCast Pipeline (tcpl) addresses the need to process data following chemical selection, procurement, and quality control (QC) for targeted high-throughput screening (HTS) assays. Processing with tcpl then enables data release and exploration in user interfaces. Each of the six levels of the ToxCast database, invitrodb, correspond to steps including pre-processing of received data to match level 0; applying tcpl to store, transform, normalize, curve-fit, and set thresholds for hit calling and potency determination; and evaluation of results (including flagging of curve-quality indicators at level 6) and final curation of assay details to make the data as informative as possible for downstream applications.

To support these efforts, the ToxCast data pipeline, tcpl, is an open-source R package that stores, manages, curve-fits, and visualizes ToxCast data as well as populating the linked MySQL Database, invitrodb (Filer et al., 2017). While developed primarily for ToxCast, the tcpl R package (USEPA, 2022b) is written to be generally applicable to the chemical-screening community, with public access to versioned releases, a manual of all functions, and informative vignettes in a Comprehensive R Archive Network (CRAN) repository^1^ and public access to the software development in a GitHub repository^2^. This flexible analysis pipeline is capable of efficiently processing and storing large volumes of heterogeneous targeted assay data. Data received in different formats from numerous vendors are transformed to a standard computable format and loaded into invitrodb by vendor-specific R scripts (Figure 1). Once data are loaded into the database, tcpl provides functions to process, normalize, model, annotate, and visualize the data. The most recent database release, invitrodb version 3.5 (USEPA, 2022d), included data for 9,541 substances, including 196 per- and polyfluoroalkyl substances (PFAS), with new endpoints related to steroidogenesis, cardiotoxicity, and neurodevelopment or neuroactivity. All ToxCast data are made fully accessible for download^3^ and accessible for browsing and downloading of summary information and plots under the Bioactivity tab of the CompTox Chemicals Dashboard^4^ (Williams et al., 2017). Many users typically focus on key summary metrics from curve-fitting concentration response data, including the qualitative response (positive or negative hit call [hitc]) and the quantitative potency at which activity may be observed, such as the activity concentration at 50% of maximal activity (AC50) or activity concentration at the cutoff for a positive (ACC).

A salient need moving forward is for curve-fitting of bioactivity data of any tier in the CompTox Blueprint to be as analogous as practicable. The primary objective of the work described herein was to implement changes in tcpl and subsequently in invitrodb to include curve-fitting models available in BMDExpress version 2 (Phillips et al., 2019) and used to curve-fit Tier 1 bioactivity data (Harrill et al., 2021; Nyffeler et al., 2021; Nyffeler et al., 2022), and then to evaluate the potential impacts on ToxCast data interpretation. To accomplish this, tcpl v3.0, released in August 2022, incorporates a new dependency for curve-fitting: R package tcplfit2 (Sheffield et al., 2021; USEPA, 2022c). The R package tcplfit2 is already employed for analysis in Tier 1 high-throughput transcriptomics (HTTr) and high-throughput phenotypic profiling (HTPP) screening results (Nyffeler et al., 2020; Harrill et al., 2021; Nyffeler et al., 2022; Speen et al., 2022). Existing annotation of ToxCast assays to gene and intended target family (a label intended to capture biological processes or types of macromolecular interaction), along with upgrading curve-fitting using tcplfit2, will enable a more seamless integration with HTPP and HTTr results. Incorporating tcplfit2 into tcpl increases the number of curve-fitting models from three to ten; permits data to be fit bidirectionally instead of unidirectionally thereby reducing the number of redundant endpoints in ToxCast; provides the user with expanded options when selecting an appropriate activity cutoff to produce potency estimates; and, produces a hitc on a continuous scale rather than a binary one. These major changes required expansive updates to tcpl and invitrodb, which are described herein. Further, we evaluate the hypothesis that using tcplfit2 curve-fitting on data included in invitrodb v3.5 and previously modeled with tcpl v2.1.0 would not significantly alter rates of positive activity calls or estimates of chemical potency. Overall, these efforts provide the software and database utilities needed to promote integration of bioactivity data from multiple tiers of bioactivity screening.

## Methods and updates

### Background

The tcpl package includes processing functionality for two screening paradigms: 1) single-concentration (sc) screening and 2) multiple-concentration (mc) screening (Filer et al., 2017). Figure 1 provides a conceptual overview of this ToxCast Pipeline functionality. Sc screening consists of testing chemicals at one concentration, often for the purpose of identifying potentially active chemicals to test in the mc format. Mc screening consists of testing chemicals across a concentration range, such that the modeled activity can give an estimate of potency, efficacy, etc. Both processing paradigms involve multiple levels of data.

Custom pre-processing scripts prepare heterogeneous source files for writing to invitrodb at level 0, where critical fields are standardized: assay component, sample, assay plate, row, and column location on each assay plate, well type, well quality, concentration of test chemical, raw value, and the source file name. Tcpl and invitrodb together provide two parallel tracks for data processing: sc at levels 0 through 2 and mc at levels 0 through 6. Processing is sequential, and every level of processing requires successful processing at the preceding level. The processing requirements vary by screening paradigm and level; however, in general, many of the processing steps require specific methods to accommodate different experimental designs or data processing approaches. In addition to storing the data, the tcpl database, invitrodb, stores every processing and analysis decision at the assay component or assay endpoint level to facilitate transparency and reproducibility. The vignettes provided within the tcpl package provide a comprehensive overview of ToxCast data processing and normalization (USEPA, 2022b). The following section will primarily describe the major software and data updates of mc processing at levels 3-5.

### Updated database interaction

Using tcpl (v3.0 and up) to manage and curve-fit data requires users to connect to their own instance of the MySQL database, invitrodb, which is used to store all data and processing decisions made by the user. The R package tcpl maintains some limited backward compatibility with versions of invitrodb that predate invitrodb v4.0 by automatically checking the schema structure of the user’s database configuration before writing data. In previous versions of tcpl (2.0.0-2.1.0), a functionality referred to as “tcplLite” relied on input of flat files formatted like the tables of invitrodb to produce curve-fitting and summary information without a database connection. Now, tcplfit2 can be used to curve-fit data and make potency estimates independent of the invitrodb schema if needed, and consequently tcplLite is no longer needed or supported.

### Modifications to the invitrodb schema

Supplementary Figure S1 in Supplementary File S1 in provides a generalized representation of the invitrodb schema and table relationships, with new (mc4_param, mc5_param, mc5_fit_categories) or impacted tables (assay_component, assay_component_endpoint, sc2, sc2_methods, mc4, mc4_methods, mc5, mc6_methods) highlighted. In the invitrodb v3.5 data model, processed data were structured in wide format with a fixed number of columns in the level 4 (mc4) and level 5 (mc5) tables based on three curve-fitting models: constant, Hill, and gain-loss. Starting in invitrodb v4.0, the mc4 and mc5 tables, and the newly added mc4_param and mc5_param tables, are in long data format, such that additional models and parameters can be added without adjusting the schema. Complete tcplfit2 model parameters are captured within the mc4_param and mc5_param tables, allowing for generic fitting and hit calling, with summary-level statistics for all models and the winning model stored in mc4 and mc5, respectively. Data and parameter tables (e.g., mc4 and mc4_param or mc5 and mc5_param) should be reviewed together if full modelling details are needed per the helper function, tcplLoadData. These schema changes provide a way to continually expand modeling capabilities in tcpl while maintaining a single data model.

### Updated pipelining methods

New curve-fitting and hit calling requirements necessitated changes in assigned pipelining methods at mc level 3 and higher in the mc pipeline. Mc processing proceeds the same as tcpl v2.1.0 at level 1 (defining replicate and concentration indices) and level 2 (assay component transformations or corrections). In previous versions of tcpl and invitrodb, dual endpoint results were registered for components that were interpretable bidirectionally, which necessitated that data be “flipped” (multiplied by negative 1) to enable curve-fitting only in the positive direction. With the addition of bidirectional curve-fitting, assay data with bidirectional response are no longer artificially flipped into the positive analysis direction by multiplying the response values by negative 1 (*i.e.*, the level 3 normalization method “resp.multneg1” was removed in these cases). For unidirectional endpoints, where data were expected to have only gain or loss of signal, such as cytotoxicity endpoints, a new method was added to prevent bidirectional fitting and fit data in only the positive analysis direction. For bidirectional endpoints, such as those measuring both induction and inhibition, the chemical’s response directionality can be inferred from the sign of the modeled top of the curve. At curve-fitting (mc level 4), the user must select a method for computing the BMAD as an estimate of the dispersion of control or baseline treatment values. For benchmark response (BMR) derivation, the user must now specify the “onesd.aeid.lowconc.twells” method to estimate 1.349 standard deviations of baseline response (Thomas et al., 2007) in the two lowest concentrations of treatment wells. Given that many endpoints utilize neutral controls to understand baseline variability, future updates may recalculate BMR as defined by neutral control wells. Newly re-developed level 6 methods, described in a later section of this paper, will provide “flags” for potentially aberrant curve-fitting behavior and will be implemented in an upcoming database release (invitrodb v4.1).

### Updated assay endpoint annotation

To connect bioactivity data with other data and improve data interpretation, invitrodb stores chemical and assay annotations. Chemical annotations within the ToxCast program are synchronized with the US EPA’s Distributed Structure-Searchable Toxicity (DSSTox) database (Grulke et al., 2019) and the associated chemical sample information. Assay, assay component, and assay endpoint annotations are developed manually to map critical information about the assay technology and the technological target, including a mapping to the most relevant National Center for Biotechnology Information (NCBI) gene identifier and the most relevant manually developed intended target families, such as “cell cycle,” “mitochondria,” and “neuroactivity.” The assay, assay component, and assay component endpoint tables store these important experimental and biological details and are exported for download as part of invitrodb and as separate summary files. These curations along with the standardized data processing procedure ensure that invitrodb follows and supports the Findable, Accessible, Interoperable, and Reusable (FAIR) Data Principles (Wilkinson et al., 2016) needed for data interoperability in workflows that utilize bioactivity data.

A key change implemented in adopting bidirectional curve-fitting in tcpl is a large reduction in the redundancy of assay endpoint data represented in invitrodb v4.0; as indicated above, there is no longer a need to create “_up” and “_dn” endpoints for the same assay data that can be interpreted in both the positive and negative direction. The assay description tables include information on the source of assay data, the assay principle and technological platform, elements measured (raw readout), and how the measurement was interpreted (normalized component data). Assay source (e.g., the vendor or laboratory), assay, assay component (e.g., the readout measured), and assay endpoint, in hierarchical order, are registered via tcpl commands into a collection of tables in invitrodb. In versions of invitrodb through v3.5, as one moves down the hierarchy, each additional level has a ‘one-to-many’ relationship with the previous level depending on the assay. For example, an assay may include multiple readouts, labeled as assay components, and then analysis of these component data independently for gain or loss of signal could result in multiple assay endpoints. An assay endpoint can derive only from a single assay component. In the past, an assay component might have two assay endpoints reflecting gain or loss of signal direction since previous versions of tcpl only allowed modeling in the positive analysis direction. Given bidirectional fitting enabled by tcplfit2, a single endpoint is now sufficient to capture both gain and loss of signal. A single assay may still result in multiple components and endpoints, *e.g.*, in the event multiple time points or aspects are measured for that assay, but for most assay components and assay endpoints, the relationship in invitrodb v4.0 and beyond will be one-to-one. Many endpoints were removed and/or renamed in invitrodb v4.0, and annotations were updated to reflect this paradigm shift as the “up” and “down” data are no longer separated into different assay endpoints to represent different curve-fitting directions. It should be noted that a subset of assay endpoints in invitrodb are designated as being meaningful only in one direction, and within tcpl fitting can be set as unidirectional at level 4. Annotations were also updated to reflect this paradigm shift, but continued curation efforts will enable better data aggregation in subsequent invitrodb releases. See Table 1 for a summary of assay, assay component, and assay component endpoint counts, as well as Supplementary Table S1 in Supplementary File S2 in for a complete mapping of invitrodb v3.5 to invitrodb v4.0 assay endpoints.

**TABLE 1:** Reduction in assay component endpoints. Bidirectional fitting, as appropriate, enabled removal of many assay component endpoints from invitrodb v4.0 that previously in invitroDB v3.5 had been curve-fit in the positive direction and then also multiplied by negative one and for curve-fit again due to curve-fitting in positive direction only with tcpl v1.2.2-2.1.0.

Assay element	Invitrodb v3.5	Invitrodb v4.0	Change
**Assay Source:** Describes the vendor or origination of the data	26	26	0
**Assay:** Describes the procedure, conducted by some vendor, to generate the component data	623	625	2
**Assay Component:** Describes the raw data readouts	1499	1496	−3
**Assay Component Endpoint:** Represents the normalized component data	2243	1496	−747
**Samples:** Distinct quantity of chemical procured and screened	46712	46712	0
**Chemicals:** Unique chemical compounds screened	9541	9541	0
**Endpoint-Samples:** Combination of unique samples screened per endpoint	3979274	3215442	−763832

### Improved curve-fitting, potency estimation, and hit calling

**FIGURE 2:**
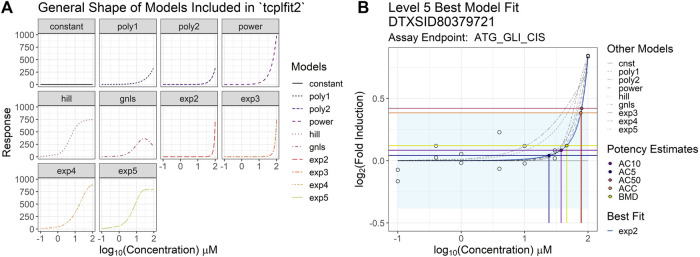
Examples of tcpl v3.0 curve-fitting **(A)** contains simulated concentration-response curves to illustrate the general underlying curve shape covered by each of the models included in the tcplfit2 package and used on the backend of the level 4 data processing in tcpl. Each sub-plot in the figure corresponds to a single parametric model included in the model fitting process and has a corresponding color and line type to accompany it. All sub-plots are plotted such that the *x*-axis represents the log10-transformed concentration, and the *y*-axis represents the response values. **(B)** illustrates the results from the Level 5 analyses in the tcpl pipeline package including the best model fit and subsequent point-of-departure (POD) estimates. The model with the lowest AIC value is selected as the winning model (modl) and is used to determine the activity hit call (hitc) for the concentration series. If two models have equal AIC values, then the simpler model (i.e., model with fewer parameters) wins. The light-blue shaded region represents the estimated efficacy cutoff (coff). Each of the concentration-response models fit in Level 4 are included in the plot, where the blue curve indicates the best model fit for the observed data (white circles) and the rest are depicted by the gray curves. The horizontal lines show the activity responses from which potency estimates of interest are defined, and the vertical lines show the corresponding POD estimates. The black point shows the AC5 (concentration producing 5% of the maximal response), the purple point shows the AC10 (concentration producing 10% of the maximal response), the yellow point shows the BMD (benchmark dose), the orange point shows the ACC (concentration producing a response at the efficacy cutoff), and the pink point shows the AC50 (concentration producing 50% of the maximal response).

Tcpl is now dependent on the tcplfit2 R package (USEPA, 2022c), which provides several key pieces of functionality, including curve-fitting all of the mc data for invitrodb. Tcplfit2 importantly expands the number of curve-fitting models in tcpl from 3 (Hill, gain-loss [a modified Hill], and constant) to 10, including linear and quadratic polynomials, power, and four exponential models, informed by BMDExpress2 (Phillips et al., 2019) and implemented previously (NTP, 2018). All ten parametric models available in tcplfit2 are used to fit each concentration-response series in a bidirectional manner. The model with the lowest Akaike Information Criterion (AIC) value is selected as the winning model (as denoted by the modl field in invitrodb) and is used to determine the activity hitc for the concentration series. Gain-loss fitting in tcplfit2 has been updated from the previous tcpl gain-loss implementation to include a more stringent minimum width difference. The minimum width difference, or the difference between the estimated gain and loss AC50 values, increased to 1.5 log10 units compared to 0.25 units, which is meant to deter aberrant single point hit scenarios, as many assays have 0.5 log10 concentration spacing. If two models have equal AIC values, the simpler model, i.e., the model with fewer parameters, wins the tie. Unlike previous versions of tcpl, the constant model never wins using tcplfit2; instead, the constant model AIC is compared to the AIC of other models to inform the first proportional weight considered in the calculation of the continuous hitc (i.e., a constant model AIC value lower than any winning model will result in hitc that approaches 0). The mathematical equations comprising the curve-fitting models are described in more detail in the tcplfit2 manual (USEPA, 2022c) and summarized in Supplementary Table S2 in Supplementary File S2. The general shapes of the 10 curve-fitting models from tcplFit2 are illustrated in Figure 2A for reference.

Curve-fitting enables determination of various metrics of potency, *i.e.*, concentrations at which some amount of *in vitro* bioactivity is expected to occur, illustrated in Figure 2B. A common potency metric used from tcpl is the activity concentration at 50% of maximal activity, or AC50, provided for the Hill and Gain-Loss models. All versions of tcpl output the activity concentration at 10% maximal response (AC10), the activity concentration at baseline (ACB, baseline defined as 3*baseline median absolute deviation, or BMAD, of the control) and activity concentration at cutoff (ACC) as defined by the user for the assay endpoint. All versions of tcpl provides methods for estimation of the baseline sampling variability, or noise around the assay controls, including calculation of the median absolute deviation over all response values given by wells that may represent baseline response (the BMAD), such as the neutral or vehicle control or the first two concentrations in the concentration series for all chemicals screened. The default baseline region is defined as ±3*BMAD⁠, and the ACB is the concentration at which the model first reaches a default of 3*BMAD⁠. Users define mc5 methods depending on assay and data type, with some common cutoff thresholds used to establish an ACC including 3*BMAD, 20% percent change, or 1.2*log10 fold-change.

With the introduction of tcplfit2, more potency metrics will be available for the 10 curve-fitting models. In addition to potency estimates mentioned above, benchmark dose (BMD) concentrations will be calculated per a similar benchmark dose modeling approach to the one used in the program BMDExpress2 (Phillips et al., 2019) (Figure 2B). In the current versions of tcpl and tcplfit2, the benchmark response (BMR) is only defined as 1.349 standard deviations of baseline response in the two lowest concentrations of treatment wells (Thomas et al., 2007). Tcplfit2 modelling outputs a BMD as defined by the BMR level. A 90% confidence interval around the BMD, bounded by the benchmark dose lower bound (BMDL) and the benchmark dose upper bound (BMDU), is also computed and provided to reflect the uncertainty in the BMD estimate. The calculation of these confidence intervals will occasionally fail due to a singular matrix inverse, and in these cases, BMDU and BMDL will not be reported. This case occurs when the data are especially noisy and the confidence interval around the BMD approaches infinity.

Additionally, activity hitc are computed as a continuous value that may be further binarized into active or inactive, depending on the level of stringency required by the user; herein, hitc <0.90 are considered inactive. This cutoff was determined from analysis of high-throughput transcriptomics (HTTr) data analyzed using tcplfit2, and comparison to known positive and negative reference chemicals. Continuous hitc in tcplfit2 (Sheffield et al., 2021; USEPA, 2022c) are defined as the product of three proportional weights representing the confidence that: 1) the AIC of the winning model is better than the constant model (i.e., winning model is not fit to background noise); 2) at least one concentration has a median response that exceeds cutoff; and, 3) the top from the winning model exceeds the cutoff. For invitrodb version 4.0, the second value of the probability considered any concentration-response pair (i.e., individual replicate) exceeding the cutoff. However, in the upcoming release invitrodb v4.1, this logic will be revised to the probability that the median response at any concentration exceeds the cutoff. The constant model may never be selected as the winning model, but if the constant model has the lowest AIC compared to other models, the calculated continuous hitc will approach zero. Users may interpret the continuous hitc into active or inactive designations based on different thresholds. Further testing through implementation of this new functionality may reveal appropriate thresholds for different use cases or specific assay technologies. Changes in hitc definitions between invitrodb v3.5 and v4.0 are summarized in Table 2.

**TABLE 2:** Hit Call and Potency Definitions.

Activity hit calls	Invitrodb v3.5	Invitrodb v4.0
Active, or positive response predicted	hitc = 1	hitc ≥0.90
Inactive, or negative/equivocal response	hitc = 0	hitc <0.90
Unable to fit	hitc = −1	hitc = 0; modl = “none”

Potency estimates		
**AC5**: Concentration producing 5% of the maximal response	NA	In mc5_param, hit_param as ac5 (µM)
**AC10:** Concentration producing 10% of the maximal response	In mc5, modl_ac10 (log10-µM)	In mc5_param, hit_param as ac10 (µM)
**AC50:** Concentration producing 50% of the maximal response	In mc5, modl_ga (log10-µM)	In mc5_param, hit_param as ac50 (µM)
**ACB:** Concentration at baseline, where baseline is defined as 0 ± 3bmad	In mc5, modl_acb (log10-µM)	NA
**ACC:** Concentration at cutoff	In mc5, modl_acc (log10-µM)	In mc5_param, hit_param as acc (µM)
**BMD:** Benchmark Dose, as defined by the Benchmark Response (BMR) level, which is estimated as one standard deviation of the baseline response for the endpoint using the two lowest concentrations across all tested chemicals	NA	In mc5_param, hit_param as bmd (bmr, bmdu, bmdl also provided) (µM)

Invitrodb v3.5 contains potency estimates in “wide” format whereas v4.0 uses the “long” format mc5_param to store all associated parameters. Potency estimates in invitrodb v3.5 were delivered in log10-µM in the mc5 table, whereas potency estimates in invitrodb v4.0 and beyond will be delivered in µM units.

Sc data, though not the focus of this analysis, also required an update; however, this update will not be available until it is implemented in invitrodb v4.1. The sc processing updates encompass new logic to allow for bidirectional responses, where an active hitc requires that the maximum absolute median response is greater than the value of the user defined cutoff. Methods to appropriately treat unidirectional sc2 data have been added to tcpl to handle future sc data processing needs (for invitrodb v4.1 and beyond).

### Fit category tree redesign

**FIGURE 3:**
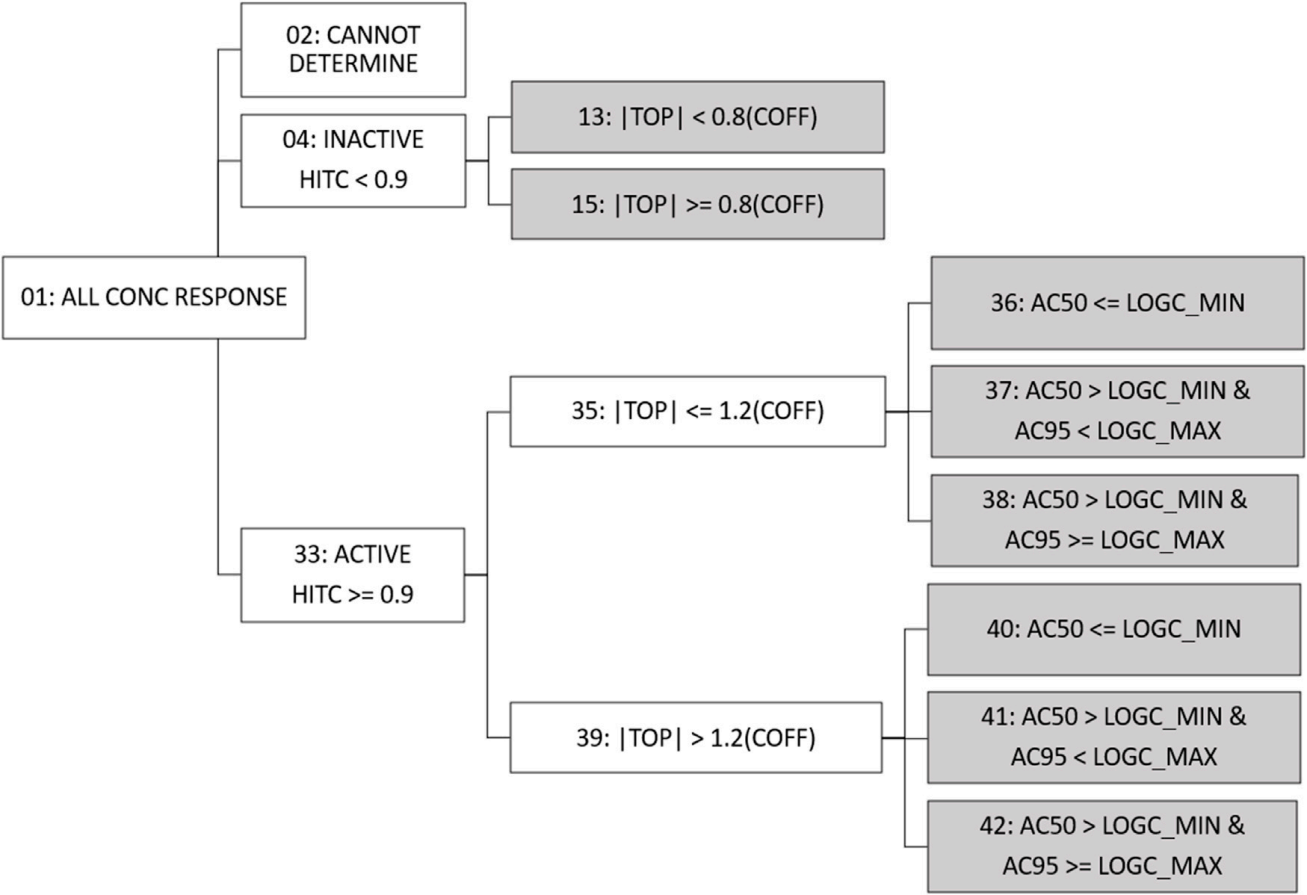
Fit Category Tree. All concentration series enter the fit category (fitc) hierarchical tree and move through activity, efficacy, and potency comparisons to assign final fitc (indicated by gray shading). For continuity purposes, fitc numbering has been conserved from past tcpl versions. Conc = concentration; hitc = hit call; |top| = absolute value of the modeled curve top; coff = cutoff; logc_min = minimum log10 concentration tested; logc_max = maximum log10 concentration tested; AC50 = 50% activity concentration; AC95 = 95% activity concentration.

Fit category (fitc) was made available in previous versions of invitrodb at level 5 as a means of encoding summary descriptions of curve behavior, based on the winning model, active or inactive designation based on hitc, efficacy, and relationship between the AC50 and the concentration range screened. Fitc was assigned to each curve (identified using the level 4 id, or “m4id”) after winning model and hitc determination. In response to the addition of more curve-fitting models, updates were needed to fitc to include any model rather than simply constant, Hill, and gain-loss. Starting with invitrodb v4.1, a more generic approach to fitc will be used to enable addition of any future curve-fitting models, where the fitc is largely based upon the relative efficacy and, in the case of actives, the location of the AC50 and concentration at 95% activity (an estimate of maximum activity concentration, AC95) compared to the tested concentration range. Figure 3 illustrates the fitc hierarchical tree used to determine the fitc. All concentration response curves are first split into active, inactive, or cannot determine. “Cannot determine” is indicative of exceptions that cannot be curve-fit, e.g., a concentration series with fewer than 4 concentrations. Active designations are determined for fitc based on whether the hitc surpasses the 0.90 threshold. For those series that are designated inactive with a hitc less than 0.90, fitc can be used to indicate to what extent the curve represents borderline inactivity via comparison of top modeled efficacy to the cutoff (i.e., the absolute value of the modeled top is less than 0.8 times the cutoff).

For active curves, efficacy, as represented by the modeled top, is compared to 1.2 times the cutoff (less than or equal to, or greater than), thereby differentiating curves that may represent borderline activity from moderate activity. Active curves also have potency metrics estimated, *e.g.*, AC50 and AC95 values, that can be compared to the range of concentrations screened to indicate curves for which potency estimates are more quantitatively informative. Curves for which the AC50 is less than or equal to the minimum concentration tested (fitc = 36, 40) may indicate AC50 values that are less quantitatively informative than AC50 values within the concentration range screened. When the AC50 is greater than the minimum concentration tested but the AC95 is greater than or equal to the maximum concentration tested (fitc = 38, 42), it is possible the maximum activity was not fully observed in the concentration range screened. Fitc for curves where the AC50 and AC95 are both within the concentration range screened (fitc = 37, 41) represent the most quantitatively informative AC50 values. In some previous applications, curves with the modeled top less than or equal to 1.2 times the cutoff and an AC50 less than the concentration range screened (fitc = 36) have been excluded from quantitative estimates of potency as potential noise resultant to overfitting (Paul Friedman et al., 2020; HealthCanada, 2021; Carstens et al., 2022).

### Revised cautionary flags

In addition to the continuous hitc and the fitc, cautionary flags on curve-fitting can provide context to interpret potential false positives (or negatives) in ToxCast data, enabling the user to decide the stringency with which to filter these targeted in vitro screening data. Cautionary flags on fitting were developed in previous versions of tcpl and have been stored at level 6. These flags are programmatically generated and indicate characteristics of a curve that need extra attention or potential anomalies in the curve or data. Many of the flags from the past versions of tcpl are re-implemented in tcpl v3.1.0+ for invitrodb v4.1, with updates to the coded logic largely to address the introduction of the BMD, bidirectional fitting, and the continuous hitc.

For example, a curve may be considered a single point hit with activity not at the highest concentration tested, but reinspection of the flagged curve could indicate a potential false positive. Other flags may suggest borderline activity, overfitting, or cell viability assays that are fit with gain-loss as the winning model. It is important to note that flags have no effect on the hitc or potency estimates, but they may indicate that a curve requires further examination to aid in data interpretation. For a full list of flags to be applied in invitrodb v4.1 and a brief description, refer to Table 3.

**TABLE 3:** Cautionary flags.

Flag name	Flag description
modl.directionality.fail	Model directionality is questionable as data points are split in positive and negative axis. tcplfit2 models assume data is zero-centered and the absolute response is increasing
low.nrep	Average number of replicates per conc is less than 2
low.nconc	Number of concentrations tested is less than 4
bmd.high	Bmd > ac50, indication of high baseline variability
singlept.hit.high	Only highest conc above baseline, active
singlept.hit.mid	Only one conc above baseline, active
multipoint.neg	Multiple points above baseline, inactive
gnls.lowconc	Complete gain-loss curve not within concentration range tested, as the “Gain” AC50 less than lowest concentration tested or the “Loss” AC50 greater than mean concentration tested
noise	Noisy data (rme > coff)
border	Borderline activity with top≤1.2*coff or top≥0.8*coff
efficacy.50	Less than 50% efficacy
ac50.lowconc	AC50 less than lowest concentration tested
viability.gnls	Cell viability assay fit with gain-loss winning model

Cautionary flags have been redeveloped for invitrodb v4.1, wherein these flags will be stored by curve-fit identifier in the level 6 table. These flags can indicate cautions on interpretation or use of these curve-fits and are determined programmatically to support users in programmatic or manual examination of curves used in their applications. The new cautionary flags in development for invitrodb v4.1 indicate potential issues such as low replicate number, hitcalls based on a single concentration above baseline, and noisy data.

### Interoperable plotting

For plotting, several tcpl functions had been used to produce the different plotting outputs. In v3.0 onwards, a single plotting function with many customizable options, tcplPlot, allows for interactive, yet consistent visualization of concentration-response curves. As a new standalone plotting utility built with the R library plotly to display the additional curve-fitting models, tcplPlot implements the R library plumber to provide representational state transfer-application programming interface (REST API) functionality, which also works with web applications. Plotly concentration-response plots are akin to the bioactivity plots surfaced via the CompTox Chemicals Dashboard. The tcplPlot function requires a level, field, and value input combination to load the necessary data and display the associated plots and tables as output. The plotting utility supports a variety of publication-quality file type options, including raster graphics (PNG, JPG, and TIFF) to retain color quality when printing to photograph and vector graphics (SVG and PDF) to retain image resolution when scaled to large formats. Further customization of outputs is possible via parameters, such as the `multi` parameter for single or multiple plots per page, or the `verbose` parameter to include a table containing potency and model performance metrics. This plotting utility update simplifies the functions in tcpl for plotting and provides standardized plotting for multiple applications, including web applications, individuals, and manuscripts.

## Results

To facilitate understanding of the impacts of tcpl updates on ToxCast data, we undertook a large experiment to compare invitrodb v3.5, processed using tcpl v.2.1.0, with invitrodb v4.0, which includes the same data as invitrodb v3.5 but reprocessed with tcpl v3.0.1 into an updated database schema to accommodate enhancements (as detailed above and summarized in Table 4). A first evident result of the upgrade to tcpl v3.0.1 is a reduction in the number of assay endpoints by 747 endpoints, resultant to bidirectional fitting. Counts of the assay sources, assays, assay components, assay component endpoints, samples, chemicals, and endpoint-samples between versions are represented in Table 1. As with any large database development, there were minor adjustments in invitrodb v4.0 related to data management, including removal of any invitrodb v3.5 endpoints (along with corresponding assays and components) that were incompletely processed in v3.5 and inclusion of a few TOX21 endpoints, containing single concentration data only, that should have been included in v3.5 and are now additions in v4.0. The number of samples and chemicals remains constant; however, there are 763,832 fewer endpoint-sample curves given the 747 endpoints that were removed since they are no longer needed given bidirectional fitting. The database comparison analysis, including all code to generate the analysis in this manuscript, and the beta invitrodb v4.0 database, are presented in Supplementary File S3 and at: https://clowder.edap-cluster.com/datasets/6451716ce4b08a6b3942fc66, with primary results summarized here.

**TABLE 4:** Invitrodb and tcpl package enhancement summary.

Enhancement	Invitrodb v3.5 and tcpl v2.0	Invitrodb v4.0 and tcpl v3.0
Curve-fitting models	Models included hill, gain-loss, and constant	In addition to hill, gain-loss, and constant, models included Polynomial 1 (Linear), Polynomial 2 (Quadratic), Power, Exponential 2, Exponential 3, Exponential 4, and Exponential 5 based on BMDExpress and encoded by R package dependency tcplFit2
Activity hit calls	Hitcall was binary: 0 = negative, 1 = positive, −1 = Unable to fit (usually due to fewer than 4 concentrations)	Hitcall is continuous as the product of three proportional weights: median response and top of model exceed the cutoff, and AIC is less than the AIC of the constant model fit
Potency estimates	Potency estimates were based on modelled active concentration series, including ACB (activity concentration at baseline, 3bmad), ACC (activity concentration at cutoff), and AC50 (activity concentration at 50% of maximal response)	Based on the program BMDExpress v2.0, tcplfit2 modelling outputs new potency and uncertainty estimates related to a benchmark dose (BMD) as defined by the Benchmark Response (BMR) level
Stand-alone pipelining	In addition to connecting to a tcpl database, tcplLite connection would create flat files structured like invitrodb for stand-alone pipelining applications	tcplLite is no longer supported by tcpl. tcplfit2*,* however, can be used for stand-alone applications, available at https://cran.r-project.org/package=tcplfit2
Endpoint structure and annotation	Tcpl only fit in the positive analysis direction therefore dual endpoints were registered to capture gain and loss of signal	Given bidirectional fitting, a single endpoint is sufficient to capture both gain and loss of signal. Many endpoints were removed and/or renamed, and annotations were updated to reflect this paradigm shift. Continued curation efforts enable better data aggregation
Schema changes	Processed data was previously stored in “wide” format with a fixed number of columns in the level 4 (mc4) and level 5 (mc5) tables based on three curve-fitting models	Complete tcplfit2 model parameters are captured within the mc4_param and mc5_param tables, allowing for generic fitting and hit calling, with summary-level statistics now only stored in mc4 and mc5
Plotting	Several functions were used to produce the different plotting outputs	tcplPlot() allows for interactive, yet consistent visualization of concentration-response curves. As a new stand-alone plotting utility built with plotly to display the additional curve-fitting models, the utility implements plumber to provide REST API functionality, which can support Docker integration and web hosting

A summary description of major updates to invitrodb in v4.0 and tcpl in v3.0 are provided in Table 4, including updates to curve-fitting models, hitcall determination, potency estimation, data pipelining independent of invitrodb (“stand-alone pipelining”), endpoint structure and annotation, changes to the invitrodb schema, and new plotting.

### Activity hitc changes

**FIGURE 4:**
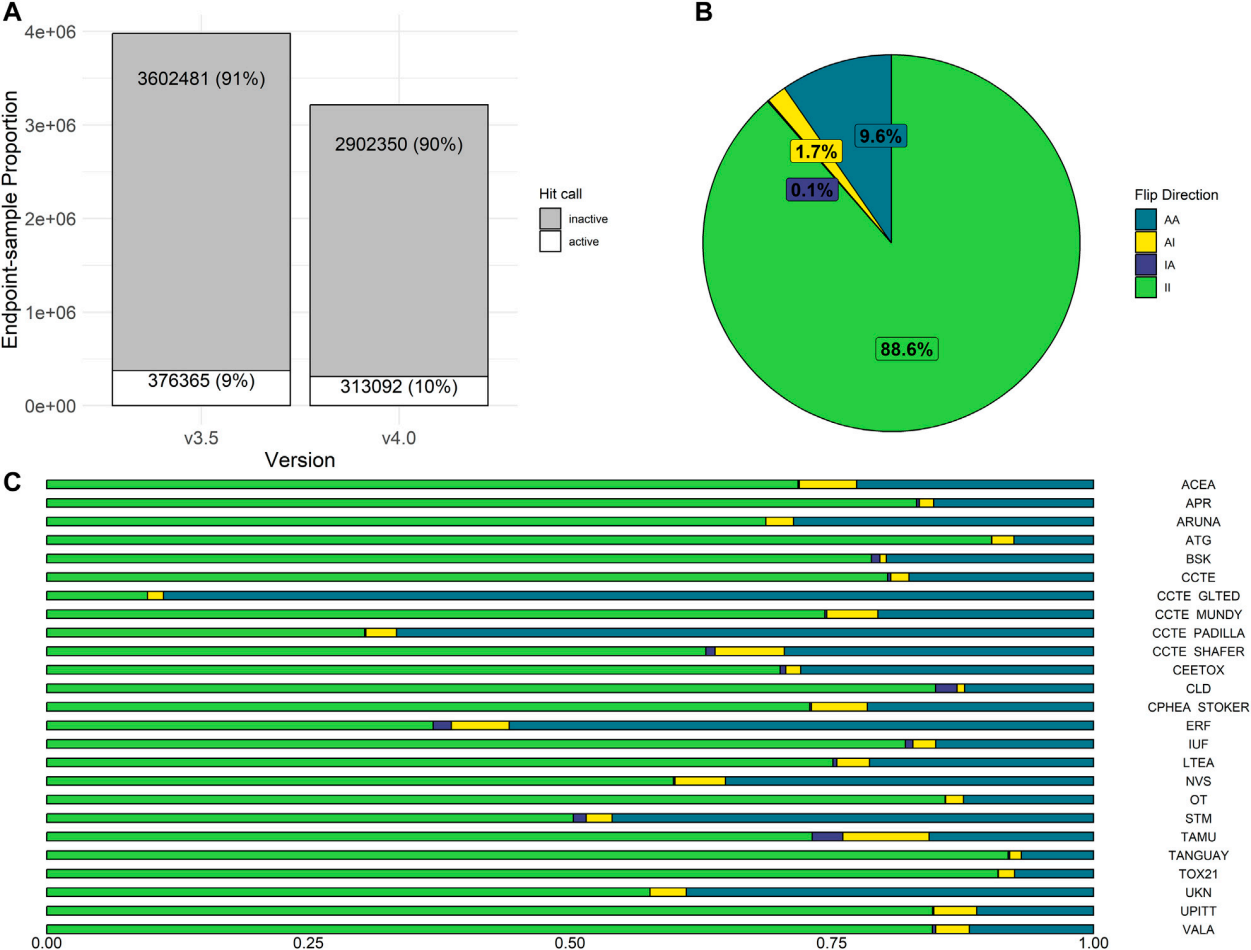
Activity Hit Call Shifts. In **(A)**, the proportion of hit calls in invitrodb versions 3.5 and 4.0 are displayed. In **(B)**, overall hit call change was examined, where possible flip directions include AA (sample was active in both v3.5 and v4.0), AI (active in v3.5, but inactive in v4.0), II (inactive in both v3.5 and v4.0), or IA (inactive in v3.5, but active in v4.0). II and AA represent no change in hit call determination. In **(C)**, hit call change across assay sources is displayed.

In invitrodb v3.5, activity hitc were binary, where 0 was negative, 1 was positive, and −1 corresponded to concentration-response series that tcpl was “unable to fit” (e.g., <4 concentrations). In invitrodb v4.0, the hitc is continuous from 0 to 1. For this analysis, a hitc greater than or equal to 0.90 was labeled active, whereas anything less was considered inactive. This threshold of 0.90 was based on other tcplfit2 implementations with *in vitro* screening data (Nyffeler et al., 2023) and reflects the apparent bimodal nature of the hitc distribution, where a preponderance of the hitc fall between 0 and 0.1 and 0.9 and 1.0 (Supplementary Figure S2 in Supplementary File S1). The “unable to fit” series now appear as model “none” with a hitc of 0 (inactive). Moving from tcpl v2.1.0 to tcpl v3.0.1 and using a hitc threshold of 0.90 resulted in very limited change in hitc rates: invitrodb v3.5 included 91% inactive and 9% active hitc whereas invitrodb v4.0 included 90% inactive and 10% active, as shown in Figure 4A.

Potential changes in individual hitc were also evaluated in aggregate by endpoint, as shown in Figure 4B. Approximately 98% of hitc remained unchanged by upgrading to tcpl v3.0.1 and implementation of tcplfit2 for curve-fitting. Possible hitc flip directions include AA (active in both invitrodb v3.5 and v4.0), AI (active in invitrodb v3.5, but inactive in v4.0), II (inactive in both invitrodb v3.5 and v4.0), or IA (inactive in invitrodb v3.5, but active in v4.0). II and AA represent no change in hitc determination for 88.6% and 9.6% of all endpoint-samples respectively, or 98.2% combined. In terms of flipped hitc, 1.7% of endpoint-samples were AI and only 0.1% converted to IA. In Figure 4C, the limited amount of change in endpoint-level hitc seems to come largely from endpoints moving from active to inactive (AI), as all 25 assay sources demonstrated a minor reduction in active hits. The majority of assay sources showed negligible relative change in actives, with only four assay sources showing greater than 10% relative change in active hitc. Across the entire database, there was a reduction in inactive hitc from deleted endpoints that were redundant due to unidirectional fitting in previous tcpl versions.

Both the number and type of invitrodb v3.5 cautionary flags may provide insight into why the hitc may have flipped between invitrodb v3.5 and invitrodb v4.0. As a baseline on cautionary flag count per curve, approximately 85% of AA curves (active in both invitrodb v3.5 and v4.0) had 1 or fewer flags (49.4% of AA curves with 0 flags and 35.2% with 1 flag). In contrast, approximately 50% of IA endpoint-samples had 1 or more flags, and 75% of AI endpoint-samples had 2 or more flags in invitrodb v3.5 (see Supplementary Figure S5 in Supplementary File S1). This suggests that curves that changed from active in invitrodb v3.5 to inactive in invitrodb v4.0 had a higher flag count in invitrodb v3.5 and may have represented less reproducibly active curves. For active hitc with no change (AA), the most frequently observed cautionary flags were “efficacy.50” (efficacy values less than 50%) and “singlept.hit.high” (single point active with activities only at the highest concentration). The most frequently observed cautionary flags for curves that went from active in invitrodb v3.5 to inactive in invitrodb v4.0 (AI) were: “efficacy.50; ” “border hit” (active with borderline activity); “overfit.hit” (active that would be changed following a small sample correction to the AIC given number of model parameters compared to concentrations tested, with automatic flagging of concentration-response series with less than 5 or 7 concentrations for Hill and gain-loss winners respectively); and, “singlept.hit.mid” (single point active with activities not at the highest concentration), suggesting that the impacts of tcpl v3.0.1 continuous hitc changes (i.e., active in invitrodb v3.5 and inactive in invitrodb v4.0) are largest for borderline and potentially overfit curves from invitrodb v3.5. The most frequently observed cautionary flag for inactive curves that became active in invitrodb v4.0 (IA) was “noise.” The ratio of the modeled top to the cutoff (top/cutoff) also suggests that most of the change in hitc occurred for curves in the range of 1- to 1.5-fold difference, i.e., for curves with limited efficacy that suggest borderline activity (Supplementary Figure S3 in Supplementary File S1).

### Winning model change

**FIGURE 5:**
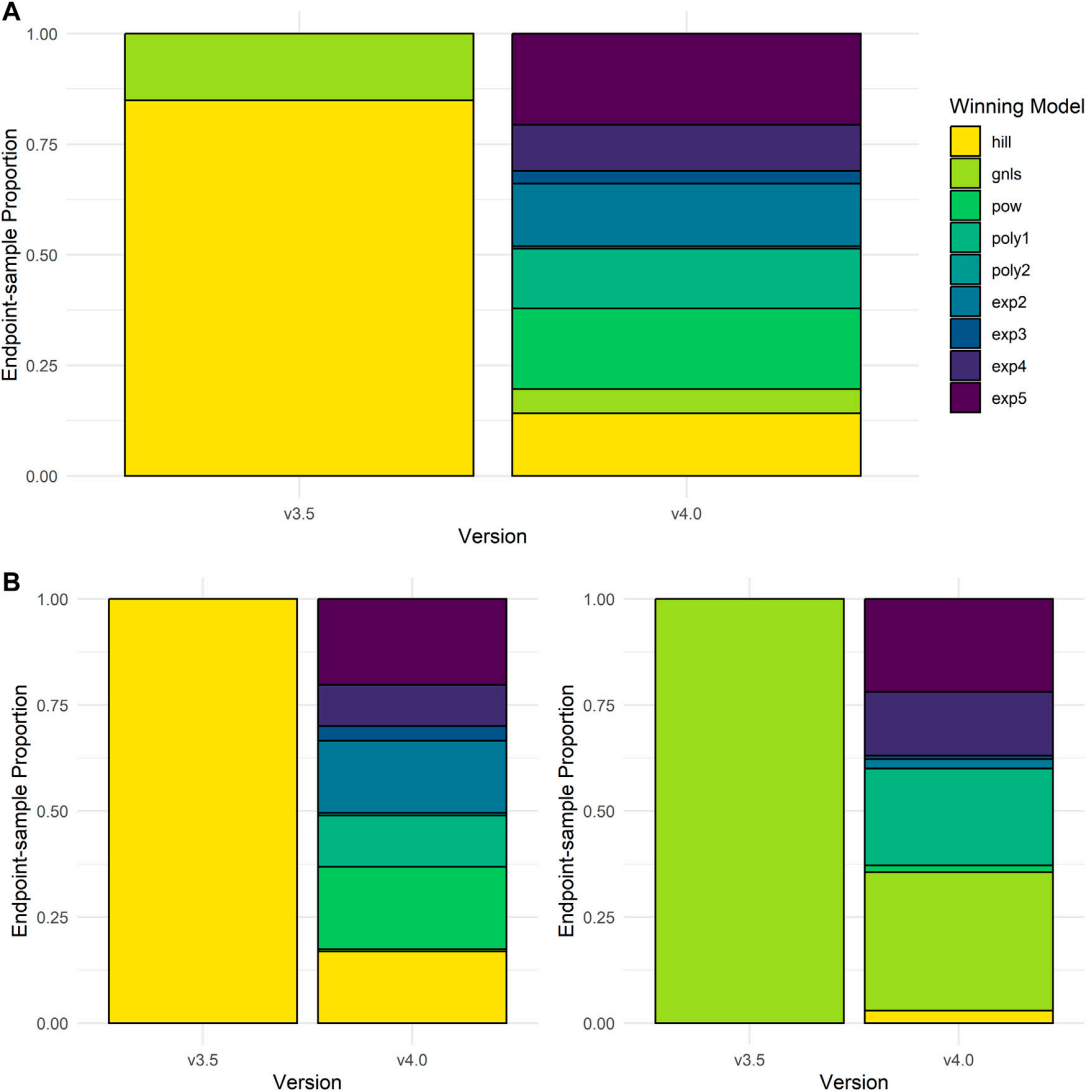
Winning Model Selection Shifts. In **(A)**, the proportion of model types that fit the active curves in invitrodb versions 3.5 and invitrodb 4.0 are compared. In **(B)**, the proportion of model types in invitrodb v4.0 that best fit the active Hill and gain-loss curves in invitrodb v3.5 are illustrated.

In re-fitting invitrodb v3.5 data using tcplfit2 curve-fitting to create invitrodb v4.0, we examined how often new models would be selected over Hill and gain loss fits. The proportion of each winning model selected for active concentration-response curves between invitrodb versions are presented in Figure 5A. In invitrodb v3.5, 85% of active hits were modeled best by a Hill fit and 15% were modeled best by a gain-loss fit. The winning model selection for active hits in v4.0 suggested that no one model type predominates using tcplfit2 curve-fitting, with 20.6% exponential-5 (exp5), 18.2% power (pow), 14.13% Hill, 14.1% exponential-2 (exp2), 13.6% linear polynomial (poly1), 10.4% exponential-4 (exp4), 5.5% gain-loss (gnls), 2.9% exponential-3 (exp3), and 0.55% quadratic polynomial (poly2). Figure 5B demonstrates the specific breakdown of how the Hill and gnls winning model fits from invitrodb v3.5 changed in invitrodb v4.0. For the 85% of active hits that were fit by a Hill in v3.5, the winning model selection in v4.0 suggests similar proportions of resultant exp5, pow, exp2, and Hill fits, with 20.25% exp5, 19.5% pow, 16.98% exp2, 16.87% Hill, along with 12.05% poly1, 9.72% exp4, 3.48% exp3, 0.64% poly2, and 0.51% gnls. For the 15% of active hits that were gnls in invitrodb v3.5, the winning model selection in v4.0 was distributed across several models, with approximately one-third of gnls fits remaining gnls: 32.66% gnls, 22.18% poly1, 21.94% exp5, 15.03% exp4, 2.93% Hill, 2.21% exp2, 1.59% pow, 0.79% exp3, and 0.04% poly1. Overall, as initially hypothesized, many active fits were distributed among the new models, likely because observing truly sigmoidal curves with full asymptotic behavior is relatively rare in high-throughput screening data using identity-blinded chemical libraries screened in the same concentration range.

### Potency change

**FIGURE 6:**
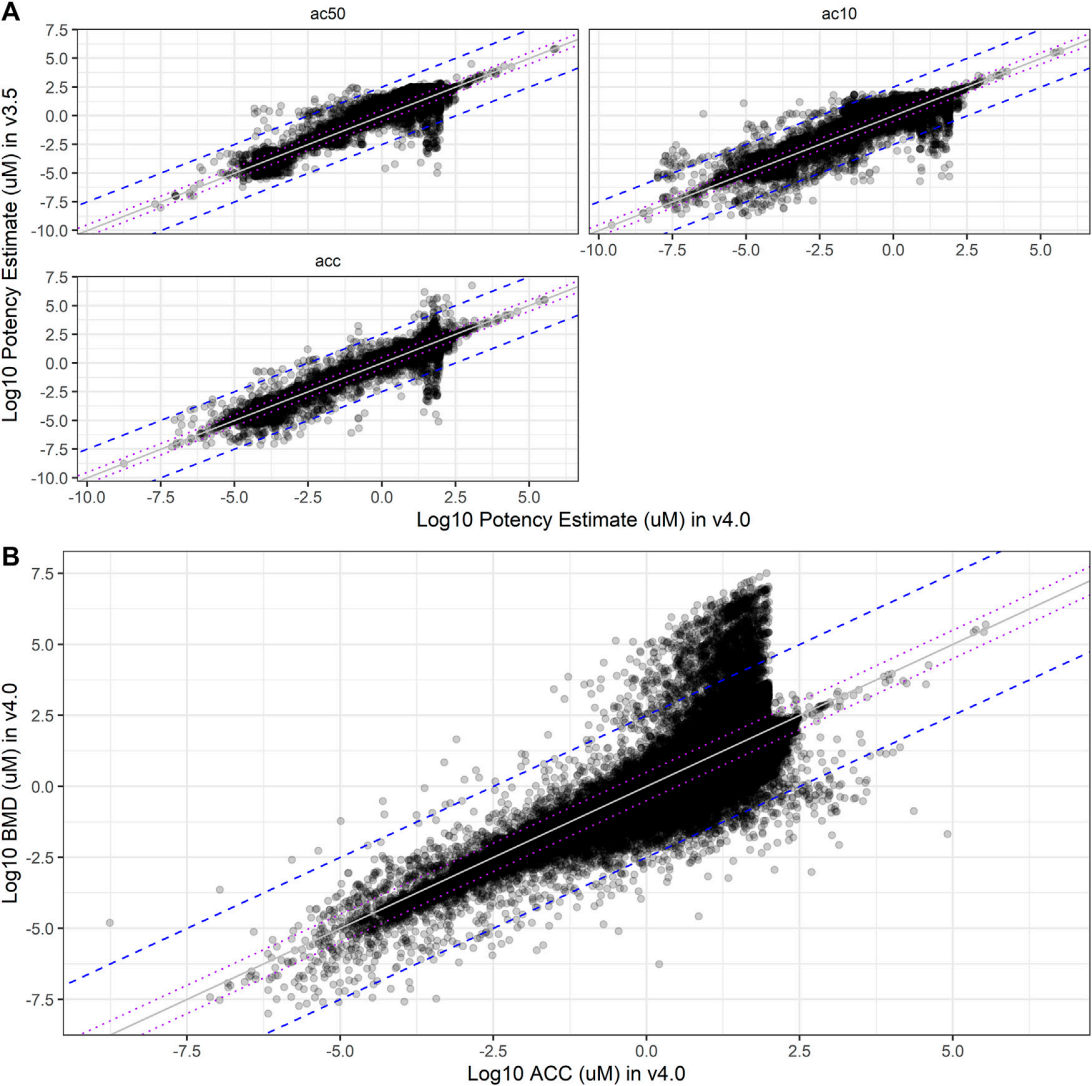
Potency Estimate Shifts. In **(A)**, comparisons of the log10-µM potency estimates from curve-fits in invitrodb v3.5 (y-axis) to invitrodb v4.0 (*x*-axis) are illustrated, including AC50, AC10, and ACC. Purple dotted lines close to unity bound ± 0.5 log10-µM, as well as the mean differences between database versions according to the RMSD values (see Table 5). The blue dashed lines further from unity bound ± 2.5 log10-µM. In **(B)**, a comparison of log10-µM BMD values to log10-µM ACC values in invitrodb v4.0 for active curves is illustrated. Purple dotted lines: ± 0.5 log10-µM; blue dashed lines: ± 2.5 log10-µM.

Critical information used from ToxCast includes summary potency metrics, such as ACC, AC10, and AC50 values. With the introduction of many more curve-fitting models and BMDs in invitrodb v4.0, questions about the differences between potency metrics in different versions of invitrodb were investigated. The changes in the definition of potency values provided between invitrodb v3.5 and 4.0 are summarized in Table 2. A null hypothesis was defined that summary potency metrics (ACC, AC10, and AC50) did not change between invitrodb v3.5 and v4.0. Direct comparison of the primary potency value types from invitrodb v3.5 and v4.0 are illustrated in Figure 6A. First, it is evident that most summary potency values, of any type, fall within −5 and 2.5 on the log10-µM scale. The direct comparison plots combined with calculation of the root mean squared deviation (RMSD) suggests that ACC and AC50 comparisons largely fall on or within 0.5 log10-µM of the unity line between invitrodb v3.5 and 4.0. To understand these differences quantitatively, the RMSD was computed along with bootstrap-resampled 95% confidence interval around these RMSD values, which suggest that AC10, ACC, and AC50 values were on average 0.28, 0.16, and 0.20 log10-µM different, respectively, between invitrodb versions (see Table 5). Some inherent variability around these potency metrics is expected (Watt and Judson, 2018), and previous interpretations and applications of these values have used a benchmark of a 0.3 log10-µM separation in potency values to be considered “different” (Paul Friedman et al., 2016; Carstens et al., 2022). The average differences in potency values between invitrodb v3.5 and v4.0 appear to be within generally observed variability in these types of data, i.e., less than 0.3 log10-µM, resulting from heterogeneous experimental designs that typically involve a limited number of replicates and concentrations. A slightly higher RMSD was observed for AC10 values, which is not unexpected, as a 10% effect value may be within baseline sampling variability (e.g., below the cutoff), whereas ACC values are at the cutoff and AC50 values may fall within a linear portion of the curve-fit, further away from baseline. In examining AA curves (Supplementary Figure S4 in Supplementary File S1), ACC distributions in invitrodb v3.5 include more extreme values on the higher end than in invitrodb v4.0, but the ACC distributions demonstrate similar central tendency between versions. AC50 distributions are nearly identical between database versions, when considering the full databases (Supplementary Figure S4A in Supplementary File S1) or the AA curves only (Supplementary Figure S4B in Supplementary File S1).

**TABLE 5:** Root mean squared difference (RMSD) in potency values.

Potency comparison	Calculation	2.5% lower bound	RMSD	97.5% upper bound
AC10	v4.0 - v3.5	0.271	0.275	0.279
AC50	v4.0 - v3.5	0.192	0.196	0.200
ACC	v4.0 - v3.5	0.154	0.158	0.163
BMD to ACC	BMD - ACC, v4.0 only	0.541	0.545	0.549

The root mean squared difference (RMSD) in potency values between database versions are (invitrodb v4.0 - invitrodbv3.5) are presented for ACC, AC10, and AC50. RMSD and lower and upper bound values are in log10-µM. The lower and upper bounds denote the lower and upper bounds of a bootstrap resampled 95% confidence interval on the RMSD estimates.

With respect to the BMD, a new potency metric introduced with tcplfit2, comparison to the ACC may provide the most informative comparison for understanding relative potency within invitrodb v4.0. The RMSD between BMD and ACC within invitrodb v4.0 suggests that these values may differ on average by 0.55 log10-µM (see Figure 6B and Table 5). Differences in the BMR and cutoff underly the differences in the BMD and ACC: though the log10-cutoff is linearly related to the log10-BMR, with an adjusted coefficient of determination of 0.7055, the mean difference (i.e., log10(cutoff) – log10(BMR)) in invitrodb v4.0 is typically less than 0.5 (with a mean of 0.210 ± standard deviation of 0.510) (Supplementary Figure S6 in Supplementary File S1). Given that the ACC and BMD are different, it is unsurprising that the ACC is greater than the BMDU ∼69% of the time (for the curves for which a BMDU could be computed). The mean ± standard deviation of the 90% confidence interval about the BMD, ranging from the BMDL to the BMDU, is 0.341 ± 0.433 log10-µM. This confidence interval size range suggests that BMD values may need to be separated, on average, by 0.3 log10-µM or more to represent truly different BMD values.

### Cytotoxicity threshold change

Estimates of chemical concentrations that elicit cytotoxicity and/or cell stress have been informative for contextualizing bioactivity screening data in ToxCast and the likelihood that these data may be confounded by assay interference resulting from cytotoxicity and/or cell stress, particularly when a parallel or in-well estimate of cell viability is unavailable. As such, general estimates of the median and lower bound concentrations that might elicit cytotoxicity and/or cell stress *in vitro* have previously been calculated using the tcpl function, tcplCytoPt, which considers activity across a suite of cell-based assays. These derived concentration threshold values have been released in the “cytotox” table of invitrodb and also provided on the CompTox Chemicals Dashboard, based on updates to previous work by Judson and others (Judson et al., 2016). In some use cases, this cytotoxicity-associated “burst” threshold may be used to infer activity that may represent a false positive response ascribed to assay interference, with the stringency of this interpretation subject to the use case. The tcplCytoPt function has been updated in response to major changes in invitrodb assay endpoint content and curve-fitting. These changes include changes in units (log10-µM to µM for stored potency data at mc level 5) and a revised requirement that a chemical included in a computation of the global median absolute deviation (MAD), an estimate of the variance expected for a chemical tested in many cytotoxicity and cell stress assays, be assayed in greater than or equal to 60 assay endpoints annotated as “burst” endpoints. Previously, it was required that the chemicals included in the global MAD calculation be assayed in all 91 cytotoxicity burst assay endpoints. Additional filtering of burst assay data was also required to ensure only losses in cell viability were included and any cell proliferation responses were excluded given the bidirectional endpoints.^5^

The resultant cytotoxicity threshold change from this update to the tcplCytoPt() function was evaluated via comparison of burst endpoint data in invitrodb v3.5 and the burst endpoint data that is present in v4.0 (noting that the actual cytotox table in invitrodb v4.0 has not been updated - rather, the cytotoxicity data was reviewed and evaluated for updates to the tcplCytoPt() function as described herein). A subset of 91 ToxCast endpoints across 7 assay sources were identified previously (stored in the assay_component_endpoint table of invitrodb v3.5 as “burst” = 1 and based on extension of previous work in Judson et al., 2015) as cytotoxicity-relevant and used to define this cytotoxicity burst concentration range. For the calculation, data were first filtered to remove any control or non-representative samples as well as any gain-loss curve-fits. For each chemical, a cytotoxicity point is defined by the median AC50 for all “burst” endpoints assayed, with a requirement that at least 3 assay endpoints be tested to report a value other than the default (1000 µM). The global MAD represents the estimated variance observed for chemicals tested in a large battery of cytotoxicity or cell stress assays; this global MAD value is then used to estimate a lower bound on the median AC50 associated with “burst” endpoints for all chemicals in the database that are positive in at least 3 “burst” endpoints. As stipulated above, for a chemical to be considered within the global MAD calculation, it must be within a highly tested set of chemicals: the chemical needs an active hit rate above 5% (3 assay endpoints) and be tested in a minimum of 60 burst assay endpoints (this latter requirement represents an update to the tcplCytoPt() function as a result of the analysis of invitrodb v4.0). A lower bound on the median cytotoxicity threshold for each chemical is calculated using the chemical-specific cytotoxicity burst median minus 3 times the global MAD (i.e., the burst lower bound equals the chemical-specific median cytotoxicity AC50—3*global MAD).

In invitrodb v3.5, the calculated global MAD was 0.25, using a previous requirement that the global MAD chemical set include chemicals tested in all 91 burst assay endpoints. Using the newly revised tcpltcplCytoPt() function to be released in tcpl v3.1.0, with a requirement that only 60 burst assay endpoints be tested for the global MAD chemical set, the re-computed global MAD for invitrodb v3.5 is 0.159 log10-µM (see Supplementary File S3). The computed global MAD for the burst assay endpoint data in invitrodb v4.0, as a beta-test and without representative sample determination, is 0.163 log10-µM (see Supplementary File S3). The overall median cytotoxicity burst value was roughly equivalent between invitrodb v3.5 and v4.0 datasets (0.05 log10-µM), and the median lower bound cytotoxicity value for all chemicals with cytotoxicity data in invitrodb was similar between datasets (−0.427 for invitrodb v3.5 and −0.439 log10-µM for invitrodb v4.0) (Supplementary Figure S7 in Supplementary File S1). In practical application, since the invitrodb v3.5 snapshot included a global MAD value of 0.250 log10-µM using the previous tcpltcplCytoPt() implementation, the lower bound on the cytotoxicity burst value will increase slightly in the next invitrodb release (v4.1) as the global MAD will be slightly smaller (approaching 0.16 log10-µM).

## Discussion

The ToxCast program continues to provide one of the largest and most impactful public repositories of targeted *in vitro* NAMs for toxicology; this resource has been transformative in the field, referenced in hundreds of publications that utilize these data in myriad applications, including but not limited to comparing environmental chemical concentrations with potential bioactivity (Schroeder et al., 2016; Blackwell et al., 2017; Li et al., 2017; Corsi et al., 2019; Alvarez et al., 2021; Baldwin et al., 2022; Loken et al., 2023), defining bioactivity:exposure ratios for prioritization of chemicals for further screening (Wetmore et al., 2015; Paul Friedman et al., 2020; HealthCanada, 2021), and use in defining adverse outcome pathways relevant for environmental chemicals (Oki et al., 2016; Fay et al., 2018; Nelms et al., 2018; Saili et al., 2019; Mortensen et al., 2022). The ToxCast Pipeline, tcpl, is the primary software utility for managing these data, and invitrodb provides an integrated database solution for storing and sharing these data in a systematic manner consistent with FAIR principles. In the work herein, we present major updates to tcpl and invitrodb, largely motivated by a desire to use a single curve-fitting approach, encoded by tcplfit2, to all tiers (Thomas et al., 2019) of *in vitro* screening data, including not only targeted NAMs in ToxCast (Tiers 2–3), but also broad Tier 1 profiling technologies such as high-throughput transcriptomics or high-throughput phenotypic profiling. Importantly, this work will enable comparison across all bioactivity data regardless of the tier or methodology as well as addition of more curve-fitting models in the future to better capture the varied response behavior observed *in vitro* NAMs. Incorporating this cross-tier curve-fitting approach also led to the elimination of database redundancy, i.e., data are no longer fit twice into separate “up” and “down” endpoints due to bidirectional curve-fitting for endpoints interpretable in both directions. Reduction in data redundancy may simplify assay annotations as well as modeling tasks that utilize ToxCast data as input. Finally, tcpl is also maturing in its interoperability with other software applications, with advancements in plotting as a demonstration. Together, these updates to tcpl and invitrodb improve the utility of ToxCast data within an integrated NAM strategy and unified open-source software approach.

Increasing the number and type of curve-fitting models applied to ToxCast data by incorporating tcplfit2 as a dependency brings greater consistency between different tiers of bioactivity data that are using the BMDExpress v2 model set (Phillips et al., 2019; Harrill et al., 2021) and results in additional benefits, including a continuous hitc logic and a more flexible pipelining and database format that can accept continued addition of concentration-response models. Interestingly, this major change in curve-fitting resulted in limited change to active hitc on an assay endpoint level (98% of assay endpoint level hitc remained consistent between invitrodb v3.5 and invitrodb v4.0) and a minimal 0.5% increase in active hitc overall between invitrodb v3.5 and v4.0. Differences in hitc seemed to occur most for curves that had 2 or more cautionary flags in invitrodb v3.5 or limited efficacy. Differences in estimates of potency between invitrodb v3.5 and invitrodb v4.0 were evaluated using RMSD, which suggested on average that AC10, ACC, and AC50 values were different by less than 0.3 log10-µM. A limitation of examining distributions of these potency metrics is that differences may occur in specific assay endpoints for specific chemicals; however, overall, the degree of change in the distribution of potency values between database versions appears reasonable given that these potency estimates have uncertainty associated with them that may approach the estimate RMSD values between invitrodb versions. Based on examination of the efficacy as “top over cutoff,” (Supplementary Figure S3 in Supplementary File S1) changes in hitc and potency estimates between database invitrodb v3.5 and v4.0 appeared to occur more frequently within responses that reflected borderline efficacy, noisy data, or overfitting behavior in invitrodb v3.5, and so it is possible these changes in hitc in invitrodb v4.0 reflect improvement.

In tcpl v1-2.1.0, an active hitc of 1 was assigned to concentration-response series where the Hill or Gain-Loss model won and both the modeled and observed maximum median responses surpassed the assigned cutoff. The continuous hit calling calculation, a product of three proportional weights, results in values between 0 and 1. The continuous hitc is binarized for use via active or inactive designations using a threshold of 0.9, but the continuous value is provided for users to make interpretations with appropriate stringency. The hitc values in invitrodb v4.0 do not appear to be normally distributed, i.e., the distribution of continuous hitc values is bimodal with many values from 0 to 0.1 and 0.9 to 1, with very few values in between 0.1 and 0.9. Hitc between 0.5 and 0.9 may indicate hitc with less confidence in its reproducibility, e.g., resultant to a noisy or a borderline response. A lower hitc threshold may be appropriate for different assays or applications that may require a less stringent readout of bioactivity as defined by the user based on investigation of specific assay endpoints.

The majority of hitc across both database versions are inactive, but there are some major differences in how inactive curves are described in invitrodb v3.5 versus invitrodb v4.0 that should be noted to promote consistent interpretation of these inactive concentration-response series. In invitrodb v3.5, the majority of inactives are fit with the constant model whereas in invitrodb v4.0, resultant to implementation of tcplfit2 in tcpl, the constant model cannot win, but a proportional weight reflecting whether the constant model should win based on AIC is incorporated into the continuous hitc logic. In both versions, potency estimates from curve-fitting inactive concentration-response series should be disregarded since they are likely uninformative, given their maximum median response likely failed to surpass the cutoff.

An important question addressed in this work, in addition to whether hitc and potency estimates would change when tcplfit2 was incorporated into tcpl, is which curve-fitting models would best describe the data, given that in invitrodb v3.5 only Hill, gain-loss, and constant were available. In invitrodb v4.0, the highest frequency of models selected as winning models for active concentration-response series were exponential-5, power, Hill, or exponential-2. Considering the series with gain-loss winning model selected in v3.5, the roughly 70% change in v4.0 for this subset could be attributed to both the required minimum width difference in tcplfit2 as well as the availability of new models which fit the data better. With the exception of gain-loss and poly2 models, which allow for biphasic responses, all other tcplfit2 models are monotonic, unimodal distributions, that fit response in a single direction. Other models (u-shaped, inverted u-shape, multi-phasic) are not currently available within tcplfit2 to model concentration-response series. Although multimodal responses are typically observed with less frequency, especially for *in vitro* responses in models that in many cases lack adaptive response capability (Varret et al., 2018; Klimenko, 2021), the set of ToxCast assays is constantly growing and as such the new tcpltcplfit2 dependency allows for easier introduction of curve-fitting models as appropriate to the assay target or assay technology. Prior to model fitting, a biological understanding of the assay may indicate applicable models, e.g., it is unlikely that biphasic gain-loss model should be applied to a cell viability assay incapable of cell proliferation wherein cells would not be expected to recover viability at higher exposure concentrations. Specific model selection or exclusion, along with model averaging, could be considered for future tcpl and tcplfit2 development.

Differences in assay design or assay target may also lead to future expansion of concentration-response modeling options in tcplfit2 and tcpl. Assay sources may need to be considered individually, especially those observed to have larger potency shifts. For instance, data from BioMAP phenotypic profiling, represented by the assay source “BioSeek” or “BSK,” has many endpoints with one to two technical replicates per concentration, 4 concentrations, and limited dynamic range of the responses, such that invitrodb v3.5 utilized a special hit calling method based on the lowest effective concentration (LOEC) for these data, defined as the lowest concentration where activity exceeded the cutoff. Although data for BSK demonstrated consistent hitc between invitrodb v3.5 and v4.0, the magnitude of change in median ACC and median AC50 was 9.9 µM (approximately 1 log10-µM) and 12.9 µM (approximately 1.11 log10- µM), respectively, which was the second highest change in median ACC and third highest change in median AC50 among assay sources between invitrodb v3.5 and invitrodb v4.0. A LOEC method has yet to be implemented for tcpl v3.0 and tcplfit2 but is of interest for future development. In addition to assay design considerations, chemical coverage of the assay screening may explain assay source differences. Some assay sources have used a tiered screening approach to screen only efficacious chemicals from sc screening in concentration-response, whereas others may have run assays comprised of a panel of assay targets for more exploratory purposes with more diverse chemical profiles. For instance, the Eurofins (ERF) assay source only includes data from a few chemicals in 150 endpoints whereas other new assay sources, e.g., Leibniz Research Institute for Environmental Medicine (IUF) and University of Konstanz (UKN), only include a few hundred chemicals across their 22 combined endpoints. In contrast, the Attagene (ATG) assay source has data from over 4000 unique substances across 222 endpoints and the Tox21 assay source generally screens over 8300 unique chemical substances for primary screening assays. The assay endpoints within data sources that include screening data from high numbers of chemical substances may be more resistant to small methodological changes in curve-fitting and resultant differences in potency values than assay sources with fewer numbers of chemicals, especially in endpoints where the two lowest concentrations in the index are used to compute BMAD.

Tcpl v3.0 and invitrodb v4.0 introduce a new potency metric for ToxCast data, the BMD, which, does not have an equivalent measure in invitrodb v3.5. BMD could be compared with the ACC to understand relative potency. In a BMD to ACC comparison for invitrodb v4.0, the RMSD approaches 0.5 log10-µM (Table 5). This is an expected result as many assay endpoints in ToxCast use a cutoff of 3*BMAD, which is likely to exceed 1.349*standard deviation of the baseline (two lowest concentration) wells. Cutoff is user-specified during pipelining and may reflect statistical, assay-specific, and biological considerations, whereas BMR is arithmetically derived the same way for all endpoints: 1.349*the standard deviation of the wells comprising the two lowest concentration indices. BMR based on 1.349*standard deviation of neutral control wells (e.g., DMSO wells) is currently not included in tcpl, although many endpoints have cutoffs based on the baseline median absolute deviation of neutral control wells. As expected, ACC is greater than the lower bound on the BMD for 90% of curves where this could be evaluated for invitrodb v4.0. Future development could potentially include the capability to adjust the BMR considering the differences between the cutoff and BMR that likely result in larger differences between ACC and BMD.

ToxCast data is supplied post-pipelining with information that users can apply to filter or “clean” the data for specific use cases with the stringency required in terms of including potential false positives. For instance, curves can be filtered by effect size using fit category, filters such as a ‘top over cutoff’ of ≥1 (Nyffeler et al., 2022), by evaluating cautionary flags for borderline activity, or examining the sheer number of cautionary flags on fitting. Cautionary flags and fitc have been used in the past to rapidly indicate curve quality, along with other potential indicators such as the reproducibility of the hitc (Watt and Judson, 2018; Paul Friedman et al., 2020; Carstens et al., 2022). Developing a new version of these flags and fitc to maintain the ability to filter curve-fit data using these metrics required the data exercise undertaken herein for invitrodb v4.0: re-fitting all data in invitrodb v3.5 with tcpl v3.0.1 and learning how the resultant curves behaved and could be described systematically. Thus, these updated features were described herein but will not be fully available until the release of invitrodb v4.1. Ongoing work continues to enrich the contextual information provided to ToxCast data users for data interpretation. This includes updates to the representative sample determination logic previously encoded in tcpl (tcpl v2.1.0 function tcplSubsetChid) as well as the cytotoxicity threshold or burst computed in tcpl (tcpl v2.1.0 function tcplCytoPt) calculation to accommodate bidirectional curve-fitting and the growing database of cell viability information. Continued work to characterize the width of the confidence interval around the BMD, and if this confidence interval width is related to curve quality, could also be evaluated. Inclusion of these features with full functionality in invitrodb v4.1 will further assist with interpretations of ToxCast in vitro screening results.

Major updates to the invitrodb schema and tcpl R software package were required to enable the expansion of functionality for the ToxCast program as described here and in the supporting software documentation (USEPA, 2022b; USEPA, 2022c). The invitrodb schema transformation enables expansion of models and functionality. Assay annotation structure has been simplified to reduce duplication of data by allowing bidirectional fitting, which will further streamline any future adoption of these data into other interoperable frameworks or annotation and linkage to Organisation for Economic Co-operation and Development Harmonized Templates. Expanded curve-fitting consistent with multiple datastreams, continuous hit calling, and introduction of the BMD and corresponding confidence interval represent key updates to tcpl logic. Despite these changes, the overall degree of change in hitc and potency estimates appears relatively small between invitrodb 3.5 and 4.0, though some assay sources may experience greater potency shifting. Further inspection of individual curve-fits in combination with flags and fit categories in future invitrodb versions will provide more utility and avenues for improvement of ToxCast as a data resource. The updates made in tcpl v3.0.1 and invitrodb v4.0 represent a critical milestone in the maturation of the ToxCast program and the resources provided to users for toxicology applications of these data. Revised sc data tables implementing bidirectional data interpretation, updates to the cytotoxicity threshold calculation, and re-implementation of cautionary flags on curve-fitting, along with any newly pipelined data that was not in invitrodb v4.0, will be made available in invitrodb v4.1 as the ToxCast program continues to iteratively improve through each release of new data.

## Data Availability

The original contributions presented in the study are included in the article’s Supplementary Material, further inquiries can be directed to the corresponding author.

## Footnotes

1 See https://cran.r-project.org/package=tcpl for versioned releases, manual, and informative vignettes.

2 See https://github.com/USEPA/CompTox-ToxCast-tcpl for development and tagged versions.

3
https://www.epa.gov/chemical-research/exploring-toxcast-data-downloadable-data

4
https://comptox.epa.gov/dashboard/

5 Note that these changes to tcplCytoPt() were implemented subsequent to creation of the invitrodb v4.0 snapshot, and as such, the cytotox table in invitrodb v4.0 matches invitrodb v3.5, and should be considered deprecated. Invitrodb v4.1 will include an updated cytotox table.

## Supplementary material

Supplementary Material is organized into three downloadable files. Supplementary File 1 is a Word .docx file that contains Supplementary Figures 1 through 6. Supplementary File 2 contains Supplementary Table 1 (for assay endpoint mapping between database versions) and Supplementary Table 2 (for tcplfit2 curve-fitting model equations). Supplementary File 3 contains a Supplementary Presentation, which is an interactive HTML report, developed using RMarkdown, that provides the code used to create all of the analyses, figures, and tables used in this work.

Supplementary Data Sheet S1 Supplementary File S1, which contains all Supplementary Figures.

Supplementary Data Sheet S2 Supplementary File S2, which contains all Supplementary tabular data.

Supplementary Presentation S1 Supplemental File S3, which is an interactive html report (provided as a .zip file).

## Abbreviations

AC10, concentration at 10% of maximal activity, µM; AC50, concentration at 50% of maximal activity, µM; AC95, concentration at 95% of maximal activity, µM; ACB, concentration at baseline activity, as defined by 3*BMAD, µM; ACC, concentration at user-defined cutoff (threshold) for a positive response, µM; BMAD, baseline median absolute deviation (measure of baseline sampling variability); BMD, benchmark dose (concentration producing the BMR with the estimated curve-fit model), µM; BMDL, lower bound estimate (5%) on the 90% confidence interval for the BMD, µM; BMDU, upper bound estimate (95%) on the 90% confidence interval for the BMD, µM; BMR, benchmark response (in this work, 1.349 standard deviations from baseline as defined by the lowest two test concentrations), units of efficacy; Fitc, fit category to describe model fitting; Hitc, activity hit call; invitrodb, database that holds ToxCast bioactivity data; mc, multiple-concentration; sc, single concentration; tcpl, ToxCast Pipeline, operationalized by open-source R software.
