# Supplementary material for: The ToxCast pipeline: updates to curve-fitting approaches and database structure
Source: Front Toxicol. 2023 Sep 21;5:1275980. doi: 10.3389/ftox.2023.1275980 (PMC10552852; doi:10.3389/ftox.2023.1275980)
Supplement: Supplementary file 1 [file Presentation1.zip › SuppFile3_tcpl_v3+_invitrodb_v4_0_comparison_release_note.html]

Supplemental File 3: tcpl v3.0+ and InvitroDB v4.0 Comparison Release Note


Code 

- Show All Code
- Hide All Code

# Supplemental File 3: tcpl v3.0+ and InvitroDB v4.0 Comparison Release Note

#### Madison Feshuk, Lori Kolaczkowski, Kurt Dunham, Sarah Davidson-Fritz, Kelly Carstens, Jason Brown, Richard Judson, Katie Paul Friedman

#### August 2023

```
# Primary Packages #
library(tcpl)
library(tcplfit2)
# Data Formatting Packages #
library(data.table)
library(DT)
library(dplyr)
library(stringr)
library(magrittr)
library(reshape2)
library(tidyr)
# Plotting Packages #
library(cowplot)
library(ggplot2)
library(gridExtra)
library(ggridges)
library(RColorBrewer)
library(colorspace)
library(viridis)
library(viridisLite)
library(patchwork)
library(lemon)
library(scales)
#library(plotly)
# Data management Packages
library(readr)
library(RMySQL)
library(readxl)
library(xlsx)
# Table Packages #
library(htmlTable)
library(kableExtra)
# Analysis Packages #
library(boot)
```

# 1 Introduction

The US EPA Toxicity Forecaster (ToxCast) program makes in vitro
medium- and high-throughput screening assay data publicly available for
the prioritization and hazard characterization of thousands of chemicals
of interest. The assays included employ a variety of technologies to
evaluate the effects of chemical exposure on diverse biological targets
from distinct proteins to more complex cellular processes like
mitochondrial toxicity, nuclear receptor signaling, immune responses,
and developmental toxicity. The ToxCast data pipeline, tcpl, is an
open-source R package (https://cran.r-project.org/web/packages/tcpl/index.html)
that stores, manages, curve-fits, and visualizes ToxCast data as well as
populating the linked MySQL Database, InvitroDB. All ToxCast data is
made accessible via the CompTox Chemicals Dashboard (https://comptox.epa.gov/dashboard/) or for download at:
https://www.epa.gov/chemical-research/exploring-toxcast-data-downloadable-data.

InvitroDB v4.0 includes the same data as InvitroDB v3.5 but
reprocessed with tcpl v3.0 in an updated database schema to accommodate
tcpl enhancements as detailed in the accompanying manuscript. This
release note examines the changes between versions.

```
# #-----------------------------------------------------------------------------------#
# # COMPILE files
# #-----------------------------------------------------------------------------------#
# #endpoint agg files
# filepath <- "L:/Lab/Toxcast_Data/toxcast_data/misc/invitrodb_v4_0_processing/version_comparison/"
# filenames <- list.files(filepath, pattern = "endpoint_agg")
# 
# endpoint_agg <- NULL
# for (fname in filenames) {
#   f <- paste0(filepath, fname)
#   dat<- as.data.table(read_excel(f))
#   endpoint_agg <- as.data.table(rbind(endpoint_agg, dat, fill=TRUE))  
# }
# endpoint_agg <- endpoint_agg[is.na(acnm_v4.0), acnm_v4.0 :=acnm] #backfill where acnm used instead of acnm_v4.0
# endpoint_agg$acnm <-NULL #drop old column
# 
# #compile potency files
# filenames <- list.files(filepath, pattern = "potency")
# 
# potency <- NULL
# for (fname in filenames) {
#   f <- paste0(filepath, fname)
#   if (length(grep("\\.xlsx", f) > 0)){
#     dat<- as.data.table(read_excel(f))}
#   else 
#     dat<- as.data.table(read.csv(f))
#   potency <- as.data.table(rbind(potency, dat, fill=TRUE))  
# }
# 
# #-----------------------------------------------------------------------------------#
# # FILTER to only EXPORT-READY ENDPOINTS BY ACID 
# #-----------------------------------------------------------------------------------#
con <- dbConnect(drv = RMySQL::MySQL(), user="_dataminer", pass="pass", db="prod_internal_invitrodb_v4_0", host="ccte-mysql-res.epa.gov")
export_ready<- dbGetQuery(con, "SELECT * FROM assay_component_endpoint where export_ready=1;")
# endpoint_agg <- endpoint_agg[endpoint_agg$acid %in% export_ready$acid,] 
# potency <- potency[potency$acid %in% export_ready$acid,]
# 
# #add assay source info to potency table for facet plots
# tcplConf(user="_dataminer", pass="pass", db="prod_internal_invitrodb_v4_0", drvr="MySQL", host="ccte-mysql-res.epa.gov")
# tcplConfList()
# aeids <- tcplLoadAeid(fld="asid",val=1:40, add.fld = c("asid", "asnm", "aid", "anm", "acid", "acnm"))
# endpoint_agg <- merge(endpoint_agg, aeids[,c("aeid", "asid", "asnm", "aid", "anm")], by.x="aeid", by.y="aeid", all.x=TRUE)
# potency <- merge(potency, aeids[,c("aeid", "asid", "asnm", "aid", "anm")], by.x="aeid", by.y="aeid", all.x=TRUE)
# 
# save(endpoint_agg, potency, file = "comparison_data.RData")

load("comparison_data.RData")
```

```
#correct flip_dir column of potency table
potency <- potency %>%
  mutate(flip_dir = ifelse(
    aenm_v4.0 != "DELETED" & act_v3.5 == "inactive" & act_v4.0 == "active" & act_flip == 0,
    "AA",
    flip_dir
  ))

#correct STM_ratio endpoint
#create table that is subset of potency table with only rows of STM ratio 
#endpoints, and apply corrections
endpoint_ratio <- potency%>%
  filter(aeid %in% c(1690, 1691))%>%
  group_by(comb_id) %>%
  fill(everything(), .direction = "downup") %>%
  slice(1)
#update act_flip and flip_dir columns to correspond with corrections
#note: unique values of both act_v3.5 and act_v4.0 are "active" and "inactive". Also note that no logic for the bidirectional case where act_flip = 1 is converted to 0 is not needed, since this endpoint is unidirectional
endpoint_ratio <- endpoint_ratio %>%
  mutate(act_flip = ifelse(act_v3.5 != act_v4.0, 1, 0)) %>%
  mutate(flip_dir = case_when(
    (act_v3.5 == "active" & act_v4.0 == "inactive") ~ "AI",
    (act_v3.5 == "inactive" & act_v4.0 == "active") ~ "IA",
    (act_v3.5 == "active" & act_v4.0 == "active") ~ "AA",
    (act_v3.5 == "inactive" & act_v4.0 == "inactive") ~ "II",
    TRUE ~ flip_dir
  ))

#remove non-corrected STM ratio endpoint rows from potency table
potency <- potency %>% 
  filter(! aeid %in% c(1690, 1691))

#add the corrected rows for STM ratio endpoint back to potency table
potency <- rbind(potency, endpoint_ratio)

#correct: set ac50 to NA if hitc==0 in invitrodb v3.5. ac50s with hitc=0 usu means the max_med failed to exceed the coff
potency <- potency[hitc_v3.5==0, ac50_v3.5:= NA]
potency <- potency[hitc_v3.5==0, acc_v3.5:= NA]
potency <- potency[hitc_v3.5==0, ac10_v3.5:= NA]

#unlog potency values
potency$ac50_raw_v3.5 <- 10^potency$ac50_v3.5
potency$ac50_raw_v4.0 <- 10^potency$ac50_v4.0
potency$acc_raw_v3.5 <- 10^potency$acc_v3.5
potency$acc_raw_v4.0 <- 10^potency$acc_v4.0
potency$ac10_raw_v3.5 <- 10^potency$ac10_v3.5
potency$ac10_raw_v4.0 <- 10^potency$ac10_v4.0
potency$bmd_raw_v4.0 <- 10^potency$bmd_v4.0

#recalculate change
potency$ac50_raw_change <- abs(potency$ac50_raw_v4.0 - potency$ac50_raw_v3.5)
potency$acc_raw_change <- abs(potency$acc_raw_v4.0 - potency$acc_raw_v3.5)
potency$ac10_raw_change <- abs(potency$ac10_raw_v4.0 - potency$ac10_raw_v3.5)

#log
potency$ac50_change <- log10(potency$ac50_raw_change)
potency$acc_change <- log10(potency$acc_raw_change)
potency$ac10_change <- log10(potency$ac10_raw_change)
```

## 1.1 Version Counts

## Version Counts

The following table includes counts of assay sources, assays, assay
components, assay component endpoints, samples, chemicals, and
endpoint-samples between v3.5 and v4.0 of InvitroDB.

```
tcplConf(user="_dataminer", pass="pass", db="prod_internal_invitrodb_v3_5", drvr="MySQL", host="ccte-mysql-res.epa.gov") 
v3.5_aeids <- tcplLoadAeid(fld="asid",val=1:40, add.fld = c("asid", "asnm", "aid", "anm", "acid", "acnm")) #already filtered to export ready

tcplConf(user="_dataminer", pass="pass", db="prod_internal_invitrodb_v4_0", drvr="MySQL", host="ccte-mysql-res.epa.gov")
v4.0_aeids <- tcplLoadAeid(fld="asid",val=1:40, add.fld = c("asid", "asnm", "aid", "anm", "acid", "acnm"))

#fill in missing asid
potency[,c( "acnm", "asid", "asnm", "aid", "anm")] <- NULL
potency <- as.data.frame(merge(potency, v3.5_aeids[,c("aeid", "asnm")], by.x="aeid", by.y="aeid", all.x=TRUE))
potency <- as.data.frame(merge(potency, v4.0_aeids[,c("aeid", "asnm")], by.x="aeid", by.y="aeid", all.x=TRUE))
potency$asnm <- as.data.table(with(potency, coalesce(asnm.x,asnm.y)))

agg_hitc_v4.0 <- potency %>% 
  filter(!aenm_v4.0=="DELETED")

#create database comparison table
Output <- c("Assay Source", 
            "Assay",
            "Assay Component", 
            "Assay Component Endpoint", 
            "Samples",
            "Chemicals",
            "Endpoint-Samples")
v3.5 <- c(length(unique(v3.5_aeids$asid)),
          length(unique(v3.5_aeids$aid)), 
          length(unique(v3.5_aeids$acid)),
          length(unique(v3.5_aeids$aeid)), 
          length(unique(potency$spid)), 
          length(unique(potency$chnm)),
          length(potency$aenm_v3.5))

v4.0 <- c(length(unique(v4.0_aeids$asid)),
          length(unique(v4.0_aeids$aid)), 
          length(unique(v4.0_aeids$acid)),
          length(unique(v4.0_aeids$aeid)),
          length(unique(agg_hitc_v4.0$spid)),
          length(unique(agg_hitc_v4.0$chnm)),
          length(agg_hitc_v4.0$aenm_v4.0))

Change <- v4.0-v3.5
Table <- data.frame(Output, v3.5, v4.0, Change)

datatable(Table,
          filter='top',
          options=list(pageLength = 15,searching=FALSE, autoWidth=FALSE,  scrollX=TRUE, initComplete = JS(
            "function(settings, json) {",
            "$('body').css({'font-family': 'Calibri'});",
            "}"
          )))
```

## 1.2 tcpl Overview

## tcpl Overview

The ToxCast data pipeline, tcpl , is an open-source R package that
stores, manages, curve-fits, and visualizes ToxCast data as well as
populating the linked MySQL Database, InvitroDB. This flexible analysis
pipeline is capable of efficiently processing and storing large volumes
of data. The tcpl package includes processing functionality for two
screening paradigms: (1) single-concentration screening and (2)
multiple-concentration screening. Figure 1 provides this conceptual
overview of the ToxCast Pipeline functionality. Single-concentration
screening consists of testing chemicals at one concentration, often for
the purpose of identifying potentially active chemicals to test in the
multiple-concentration format. Multiple-concentration screening consists
of testing chemicals across a concentration range, such that the modeled
activity can give an estimate of potency, efficacy, etc. The diverse
data, received in heterogeneous formats from numerous vendors, are
transformed to a standard computable format and loaded into the tcpl
database by vendor-specific R scripts. Once data is loaded into the
database, ToxCast utilizes generalized processing functions provided in
this package to process, normalize, model, qualify, and visualize the
data.

Tcpl v3.0 was released in August 2022, which is a paradigm shift from
past versions by incorporating the tcplFit2 dependency package (https://cran.r-project.org/web/packages/tcplfit2/index.html)
for curve fitting and hit calling. The original tcplFit() functions
performed basic concentration response curve fitting with the constant,
hill, and gain-loss models. The main set of extensions includes all of
the concentration-response models that are contained in the program
BMDExpress. These include polynomial, exponential and power functions in
addition to the original Hill, gain-loss and constant models. Similar to
the program BMDExpress, tcplFit2 curve-fitting uses a defined Benchmark
Response (BMR) level to estimate a benchmark dose (BMD), which is the
concentration where the curve-fit intersects with this BMR threshold.
One final addition was to let the hitcall value be a continuous number
ranging from 0 to 1 (in contrast to binary hitcall values from
tcplFit(). In v3.5, a hit call of 0 was negative and 1 was positive. A
hit call of -1 corresponded to a “NA” model fitting, where the
concentration-response series was unable to be fit, usually due to
limited number of concentrations tested. For simplicity, “-1” hit calls
were considered inactive. In v4.0, hit calls are continuous as the
product of three probabilities: 1) probability the median response
exceeds the cutoff, 2) and probability the top of model exceeds the
cutoff, 3) and probability winning model’s AIC is less than that of the
constant model. Tcplfit2 is employed for analysis in Tier 1
high-throughput transcriptomics (HTTr) and high-throughput phenotypic
profiling (HTPP) screening results.

### 1.2.1 General Shapes of Models

This figure contains simulated concentration-response curves to
illustrate the general underlying curve shape covered by each of the
models included in the tcplFit2 package and used on the back-end of the
level 4 data processing in tcpl. Each sub-plot in the figure corresponds
to a single parametric model included in the model fitting process and
has a corresponding color and line type to accompany it. All sub-plots
are plotted such that the x-axis represents the log-transformed
concentration (base=10) and the y-axis represents the response
values.

```
## Example Data ##
# Load mc3 data for example endpoint
mc3 <- tcpl::tcplPrepOtpt(tcpl::tcplLoadData(lvl = 3L, type = 'mc', fld = 'aeid',val = 80) ) %>%
  dplyr::filter(spid == "01504209") # Level 3 - conc-resp series
# example fit concentration series
ex_conc <- seq(10^(min(mc3[,logc])),10^(max(mc3[,logc])),length.out = 100)

## Obtain the Continuous Fit of Level 4 Model Estimates ##
fits <- data.frame(
  # log-scale concentrations
  logc = log10(ex_conc),
  # parametric model fits from `tcplfit2`
  constant = tcplfit2::cnst(ps = c(er = 0.1),ex_conc),
  poly1 = tcplfit2::poly1(ps = c(a = 3.5,er = 0.1),x = ex_conc),
  poly2 = tcplfit2::poly2(ps = c(a = 0.13,b = 2,er = 0.1),x = ex_conc),
  power = tcplfit2::pow(ps = c(a = 1.23,p = 1.45,er = 0.1),x = ex_conc),
  hill = tcplfit2::hillfn(ps = c(tp = 750,ga = 5,p = 1.76,er = 0.1),
                          x = ex_conc),
  gnls = tcplfit2::gnls(ps = c(tp = 750,ga = 15,p = 1.45,la = 50,q = 1.34,
                               er = 0.1),
                        x = ex_conc),
  exp2 = tcplfit2::exp2(ps = c(a = 0.45,b = 13.5,er = 0.1),
                        x = ex_conc),
  exp3 = tcplfit2::exp3(ps = c(a = 1.67,b = 12.5,p = 0.87,er = 0.1),
                        x = ex_conc),
  exp4 = tcplfit2::exp4(ps = c(tp = 895,ga = 15,er = 0.1),x = ex_conc),
  exp5 = tcplfit2::exp5(ps = c(tp = 793,ga = 6.25,p = 1.25,er = 0.1),
                        x = ex_conc)
) %>% 
  reshape2::melt(data = .,measure.vars = c(
    "constant",
    "poly1","poly2","power",
    "hill","gnls","exp2","exp3","exp4","exp5"
  ))

## Updated Colors ##
fit_cols <-
  # choose 10 distinct colors
  viridis::magma(n = 10,direction = 1) %>% 
  # darken the original colors to make them more visible
  colorspace::darken(.,amount = 0.2)

## Plot ##
fig2a <- fits %>%
  ggplot()+
  geom_line(aes(x = logc,y = value,lty = variable,colour = variable))+
  facet_wrap(facets = "variable")+
  theme_bw()+
  labs(lty = "Models",colour = "Models")+
  theme(text = element_text(size=15))+
  scale_colour_manual(values = fit_cols)+
  ggtitle("General Shape of Models Included in `tcplfit2`")+
  xlab(expression(paste(log[10],"(Concentration) ",mu,"M")))+
  ylab("Response")

fig2a
```

### 1.2.2 Level 4 Curvefitting

This figure illustrates the results from the Level 4 analyses in the
tcpl pipeline. The plot depicts the observed concentration-response data
with white circles, where the x-axis is base 10 log-transformed
concentration values. All the ten model fits are displayed and
distinguished by color and line-type.

```
## Obtain Data ##
# #subset for a reference chemical by spid
# chnm <- "17beta-Estradiol"
# chem <- tcplLoadChem(field = 'chnm',val = chnm)
# mc3 <- mc3[spid == "TP0000306E01",]

# First, we need to obtain the subset of data related to spid = "01504209",
# which is our example spid.
mc3_ss <- tcpl::tcplPrepOtpt(tcpl::tcplLoadData(lvl = 3L, type = 'mc', fld = 'aeid',val = 80) ) %>%
  dplyr::filter(spid == "01504209") # Level 3 - conc-resp series
mc4 <- tcpl::tcplPrepOtpt(tcpl::tcplLoadData(lvl = 4L, type = 'mc', fld = 'aeid',val = 80, add.fld = TRUE) ) %>% 
  dplyr::filter(spid == "01504209") # Level 4 - model fits
mc5_ss <- tcpl::tcplPrepOtpt(tcpl::tcplLoadData(lvl = 5L, type = 'mc', fld = 'aeid',val = 80, add.fld=TRUE) ) %>% 
  dplyr::filter(spid == "01504209") # Level 5 - best fit & est.
# Next, we need to obtain the smooth curve estimate for the best model found
# in the Level 5 analyses of the `tcpl` pipeline.

## Create a Sequence of Concentration Values within Observed Range ##
X <- seq(
 10^(mc4[which(mc4[,spid] == "01504209"),logc_min]),
 10^(mc4[which(mc4[,spid] == "01504209"),logc_max]),
 length.out = 100
)
## Obtain the Continuous Fit of Level 4 Model Estimates ##
# Apply each model fit to continous concentration values (X) and estimated
# parameters from 'tcplfit2'.
estDR <- mc4 %>% 
  dplyr::filter(spid == "01504209") %>% 
  dplyr::summarise(
    cnst  = tcplfit2::cnst(.[,c(cnst_er)],X),
    poly1 = tcplfit2::poly1(.[,c(poly1_a,poly1_er)],X),
    poly2 = tcplfit2::poly2(.[,c(poly2_a,poly2_b,poly2_er)],X),
    power = tcplfit2::pow(.[,c(pow_a,pow_p,pow_er)],X),
    hill  = tcplfit2::hillfn(.[,c(hill_tp,hill_ga,hill_p)],X),
    gnls  = tcplfit2::gnls(.[,c(gnls_tp,gnls_ga,gnls_p,gnls_la,gnls_q,gnls_er)],
                           x = X),
    exp2  = tcplfit2::exp2(.[,c(exp2_a,exp2_b,exp2_er)],x = X),
    exp3  = tcplfit2::exp3(.[,c(exp3_a,exp3_b,exp3_p,exp3_er)],x = X),
    exp4  = tcplfit2::exp4(.[,c(exp4_tp,exp4_ga,exp4_er)],x = X),
    exp5  = tcplfit2::exp5(.[,c(exp5_tp,exp5_ga,exp5_p,exp5_er)],x = X)
  )
# Format data into a data.frame for ease of plotting.
estDR <- cbind.data.frame(X,estDR) %>%
  reshape2::melt(data = .,measure.vars = c(
    "cnst","poly1","poly2","power","hill","gnls","exp2","exp3","exp4","exp5"
  ))

## Updated Colors ##
fit_cols <-
  # choose 10 distinct colors
  viridis::magma(n = 10,direction = 1) %>% 
  # darken the original colors to make them more visible
  colorspace::darken(.,amount = 0.2)

## Plot the Model Fits from Level 4 ##
mc3 %>% 
  dplyr::filter(spid == "01504209") %>% 
  ggplot(.,aes(x = logc,y = resp))+
  geom_point(pch = 1,size = 2)+
  geom_line(data = estDR,
            aes(x = log10(X),y = value,colour = variable,lty = variable))+
  labs(colour = "Models",lty = "Models")+
  scale_colour_manual(values = fit_cols)+
  xlab(expression(paste(log[10],"(Concentration) ",mu,"M")))+
  ylab(expression(paste(log[2],"(Fold Induction)")))+
  ggtitle(
    label = paste("Level 4 Model Fits",
                  mc4[which(mc4[,spid] == "01504209"),dsstox_substance_id],
                  sep = "\n"),
    subtitle = paste("Assay Endpoint: ",
                     mc4[which(mc4[,spid] == "01504209"),aenm]))+
  theme(text = element_text(size=15))+
  theme_bw()
```

```
estDR <- estDR %>%
  dplyr::mutate(.,best_modl = ifelse(variable == mc5_ss[,modl],
                                     yes = "best model",no = NA))
```

### 1.2.3 Level 5 Winning Model Selection

This figure illustrates the results from the Level 5 analyses in the
tcpl pipeline package including the best model fit and subsequent
point-of-departure (POD) estimates. The model with the lowest AIC value
is selected as the winning model (modl), and is used to determine the
activity (or hit call) for the concentration series. If two models have
equal AIC values, then the simpler model (i.e. model with fewer
parameters) wins. The light-blue shaded region represents the estimated
efficacy cutoff (coff). Each of the concentration-response models fit in
Level 4 are included in the plot, where the blue curve indicates the
best model fit for the observed data (white circles) and the rest are
depicted by the gray curves. The horizontal lines show the activity
responses from which potency estimates of interest are defined, and the
vertical lines show the corresponding POD estimates. The black point
shows the AC5 (concentration producing 5% of the maximal response), the
purple point shows the AC10 (concentration producing 10% of the maximal
response), the yellow point shows the BMD (benchmark dose), the orange
point shows the ACC (concentration producing a response at the efficacy
cutoff), and the pink point shows the AC50 (concentration producing 50%
of the maximal response).

```
## Generate a Base Concentration-Response Plot ##
basePlot <- mc3_ss %>% 
  # Observed Concentration-Response Data
  ggplot()+
  geom_point(aes(x = logc,y = resp),pch = 1,size = 2)+
  # Cutoff Band
  geom_rect(data = mc5_ss,
            aes(xmin = logc_min,xmax = logc_max,ymin = -coff,ymax = coff),
            alpha = 0.15,fill = "skyblue")+
  # Best Model Fit
  geom_line(data = dplyr::filter(estDR,variable == mc5_ss[,modl]),
            aes(x = log10(X),y = value,color = mc5_ss[,modl]))+
  scale_colour_manual(values = c("royalblue3"),aesthetics = "color")+
  # Other Model Fits
  geom_line(data = dplyr::filter(estDR,variable != mc5_ss[,modl]),
            aes(x = log10(X),y = value,lty = variable),
            alpha = 0.3,show.legend = TRUE)+
  # Legend Information
  labs(lty = "Other Models",color = "Best Fit")+
  # Titles and Labels
  xlab(expression(paste(log[10],"(Concentration) ",mu,"M")))+
  ylab(expression(paste(log[2],"(Fold Induction)")))+# )+
  ggtitle(
    label = paste("Level 5 Best Model Fit",
                  mc4[which(mc4[,spid] == "01504209"),dsstox_substance_id],
                  sep = "\n"),
    subtitle = paste("Assay Endpoint: ",
                     mc4[which(mc4[,spid] == "01504209"),aenm]))+
  # Background Plot Theme
  theme_bw()

## Potency Estimate Layers ##
# First, we need to obtain/assign colors for the potency estimates to be
# displayed.
potency_cols <-
  # choose 5 distinct colors
  viridis::plasma(n = 5,direction = -1) %>% 
  # darken the original colors to make them more visible
  colorspace::darken(.,amount = 0.1)
  
## Compile the Full Level 5 Plot ##
linePlot <-
  # Start with the `basePlot` object.
  basePlot +
  # Next, add the various potency layers.
  # BMD
  geom_hline(
    data = mc5_ss,
    aes(yintercept = bmr),
    col = potency_cols[1]
  ) +
  geom_segment(
    data = mc5_ss,
    aes(x = log10(bmd), xend = log10(bmd), y = -0.5, yend = bmr),
    col = potency_cols[1]
  ) +
  geom_hline(
    data = mc5_ss,
    aes(yintercept = coff),
    col = potency_cols[2]
  ) +
  geom_segment(
    data = mc5_ss,
    aes(x = log10(acc), xend = log10(acc), y = -0.5, yend = coff),
    col = potency_cols[2]
  ) +
  geom_hline(
    data = mc5_ss,
    aes(yintercept = max_med * 0.5),
    col = potency_cols[3]
  ) +
  geom_segment(
    data = mc5_ss,
    aes(
      x = log10(ac50), xend = log10(ac50),
      y = -0.5, yend = max_med * 0.5
    ),
    col = potency_cols[3]
  ) +
  geom_hline(
    data = mc5_ss,
    aes(yintercept = max_med * 0.1),
    col = potency_cols[4]
  ) +
  geom_segment(
    data = mc5_ss,
    aes(
      x = log10(ac10), xend = log10(ac10),
      y = -0.5, yend = max_med * 0.1
    ),
    col = potency_cols[4]
  ) +
  geom_hline(
    data = mc5_ss,
    aes(yintercept = max_med * 0.05),
    col = potency_cols[5]
  ) +
  geom_segment(
    data = mc5_ss,
    aes(
      x = log10(ac5), xend = log10(ac5),
      y = -0.5, yend = max_med * 0.05
    ),
    col = potency_cols[5]
  )


# create data table for potency estimate points
mc5_points <- mc5_ss %>%
  select(bmd, acc, ac50, ac10, ac5) %>%
  tidyr::pivot_longer(everything(), names_to = "Potency Estimates") %>%
  mutate(x = log10(value)) %>%
  mutate(mc_color = potency_cols) %>%
  mutate(`Potency Estimates` = toupper(`Potency Estimates`))
yvals <- mc5_ss %>%
  select(bmr, coff, max_med) %>%
  tidyr::pivot_longer(everything()) %>%
  select(value) %>%
  mutate(reps = c(1, 1, 3)) %>%
  tidyr::uncount(reps) %>%
  mutate(y = value * c(1, 1, .5, .1, .05)) %>%
  select(y)
mc5_points <- mc5_points %>% cbind(yvals)

# add Potency Estimate Points and set colors
fig2b<- linePlot + geom_point(
  data = mc5_points,
  aes(x = x, y = y, fill = `Potency Estimates`), shape = 21, cex = 1.5) +
  theme(text = element_text(size=15), 
            # legend.title=element_blank(),
            legend.margin=margin(c(1,2,2,2)),
            legend.key.size = unit(.3, 'cm'))+
  scale_fill_manual(values = mc5_points %>% arrange(`Potency Estimates`) %>% pull(mc_color))


## Display the Compiled Plot ##
fig2b
```

```
fig2_all <- plot_grid(fig2a, fig2b, labels = c("A", "B"), label_size = 14, ncol=2, rel_widths = c(1,1),
          rel_heights = c(1,1))
fig2_all
```

```
file.dir <- paste(getwd(), sep="")
file.name <- paste("/Fig2_ex_fits", Sys.Date(), ".png", sep="")
file.path <- paste(file.dir, file.name, sep="")
dir.create(path=file.dir, showWarnings = FALSE, recursive = TRUE)
png(width=8000, height=3000, res=600)
fig2_all
dev.off()
```

### 1.2.4 Model Details

After the summary values are obtained for each concentration-response
series, all ten parametric models available in 
tcplFit2  are used to fit each series. Model details are provided
below.

|  |  |  |  |  |
| --- | --- | --- | --- | --- |
| *tcplfit2* model details. | | | | |
| Model | Abbreviation | Equations | OutputParameters | Details |
| Constant | cnst | \(f(x) = 0\) |  | Parameters always equals ‘er’. |
| Linear | poly1 | \(f(x) = ax\) | a (y-scale) |  |
| Quadratic | poly2 | \(f(x) = a(\frac{x}{b}+(\frac{x}{b})^{2})\) | a (y-scale), b (x-scale) |  |
| Power | pow | \(f(x) = ax^p\) | a (y-scale), p (power) |  |
| Hill | hill | \(f(x) = \frac{tp}{1 + (\frac{ga}{x})^{p}}\) | tp (top), ga (gain AC50), p (gain-power) | Concentrations are converted internally to log10 units and optimized with f(x) = tp/(1 + 10^(p\*(gax))), then ga and ga\_sd are converted back to regular units before returning. |
| Gain-Loss | gnls | \(f(x) = \frac{tp}{(1 + (\frac{ga}{x})^{p} )(1 + (\frac{x}{la})^{q} )}\) | tp (top), ga (gain AC50), p (gain power), la (loss AC50), q (loss power) | Concentrations are converted internally to log10 units and optimized with f(x) = tp/[(1 + 10^(p*(gax)))(1 + 10^(q*(x-la)))], then ga, la, ga\_sd, and la\_sd are converted back to regular units before returning. |
| Exponential 2 | exp2 | \(f(x) = a\*(exp(\frac{x}{b}) - 1)\) | a (y-scale), b (x-scale) |  |
| Exponential 3 | exp3 | \(f(x) = a\*(exp((\frac{x}{b})^{p}) - 1)\) | a (y-scale), b (x-scale), p (power) |  |
| Exponential 4 | exp4 | \(f(x) = tp\*(1-2^{\frac{-x}{ga}})\) | tp (top), ga (AC50) |  |
| Exponential 5 | exp5 | \(f(x) = tp\*(1-2^{-(\frac{x}{ga})^{p}})\) | tp (top), ga (AC50), p (power) |  |
|  |  |  |  |  |
| --- | --- | --- | --- | --- |
| Model descriptions are pulled from tcplFit2 manual at https://cran.r-project.org/package=tcplfit2/tcplfit2.pdf. | | | | |

## 1.3 Database Coverage

## Database Coverage

The following visuals may provide additional context about InvitroDB
database coverage. InvitroDB’s assay description tables include
information on source of the assay data, the assay principle and
technological platform, elements measured (raw readout), and how the
measurement was interpreted (normalized component data). Assay source,
assay, assay component, and assay endpoint, in hierarchical order, are
registered via tcpl commands into a collection of tables in
InvitroDB.

Complete annotations and assay description documentation are
available for download at https://www.epa.gov/chemical-research/downloadable-computational-toxicology-data.
The curation team are consistently refining annotations to better
reflect the assay design and support better data aggregation.

A list of chemicals included in InvitroDB with presence in associated
endpoints:

```
chemlist <- potency %>% 
                filter(!aenm_v4.0=="DELETED") %>% 
                distinct(chnm, aenm_v4.0)%>%
                group_by(chnm) %>%
                count(chnm)
setnames(chemlist, "n", "endpoints")

datatable(chemlist, 
          filter='top', 
          options=list(pagelength=25, autoWidth=FALSE,  scrollX=TRUE, initComplete = JS(
    "function(settings, json) {",
    "$('body').css({'font-family': 'Calibri'});",
    "}"
  )))
```

# 2 Aggregate Database Comparison

Differences across the whole database between versions can be
examined.

## 2.1 Hit Calls

## Hit Calls

In v3.5, hit call was binary, where 0 was negative and 1 was
positive. A hit call of -1 corresponded to a “NA” model fitting, where
the concentration-response series was unable to be fit, usually due to
limited number of concentrations tested. For simplicity, “-1” hit calls
were considered inactive.

In v4.0, hit calls are continuous as the product of three
probabilities: 1) probability the median response exceeds the cutoff, 2)
and probability the top of model exceeds the cutoff, 3) and probability
winning model’s AIC is less than that of the constant model. The “unable
to fit” series appear as model “none” with a hit call of 0, therefore
inactive. In the new tcpl paradigm, constant model can never win, but
the probability is account in continuous hit call.

Comparing the relative proportions of activity hit calls, v3.5
included 91% inactive and 9% active hit calls whereas v4.0 included 90%
inactive and 10% active

```
#-----------------------------------------------------------------------------------#
# AGGREGRATE DATABASE COMPARISON (HITS AND WINNING MODEL DISTRIBUTION)
#-----------------------------------------------------------------------------------#
agg_hitc_v3.5 <- potency %>% 
  group_by(act_v3.5) %>% 
  count() %>% 
  ungroup() %>% 
  mutate(perc = `n` / sum(`n`)) %>% 
  arrange(perc) %>%
  mutate(labels = scales::percent(perc))
agg_hitc_v3.5$version <- "v3.5"
setnames(agg_hitc_v3.5, "act_v3.5", "group")

agg_hitc_v4.0 <- potency %>% 
  filter(!aenm_v4.0=="DELETED") %>% 
  group_by(act_v4.0) %>% 
  count() %>% 
  ungroup() %>% 
  mutate(perc = `n` / sum(`n`)) %>% 
  arrange(perc) %>%
  mutate(labels = scales::percent(perc))
agg_hitc_v4.0$version <- "v4.0"
setnames(agg_hitc_v4.0, "act_v4.0", "group")

agg_hitc<- rbind(agg_hitc_v4.0, agg_hitc_v3.5)

agg_hitc$group <- factor(agg_hitc$group, levels = c("inactive", "active"))
```

```
fig4a <- ggplot(agg_hitc, aes(x = version, y= n, fill = group)) + 
  geom_bar(colour="black", stat = "identity") + 
  theme_minimal() +
  #scale_fill_manual( values = c(  "#5F96C2",  "#C87A8A"))+
  scale_fill_manual(values=c('gray', 'white'))+
  guides(fill = guide_legend(title = "Hit call")) +
  labs(y = "Endpoint-sample Proportion", x= "Version") +
  theme(axis.text = element_text(size=14),
        axis.title = element_text(size=16))+
  geom_text(aes(label = paste0(n, " (", labels, ")")), size=5, vjust = 1.2)

fig4a
```

When examined, the distribution of the v4.0 continuous hit call is
not normally distributed.

```
agg_hitc_dist <- potency %>% 
  filter(!aenm_v4.0=="DELETED")
```

```
#hist(agg_hitc_dist$hitc_v4.0, main="Distribution of v4.0 Continuous Hit Calls",
#     xlab="Hit Call")

suppfig2 <- ggplot(data=agg_hitc_dist) +
  geom_histogram(aes(x=hitc_v4.0), bins=10)+ 
  xlab('Hitcall')+
  ylab('Frequency of Hitcall')+
  scale_x_continuous(breaks=seq(0,1,0.1))+
 # scale_x_continuous(trans=log10_trans(),
  #                   #breaks=trans_breaks("log10", function(x) 10^x),
   #                  breaks=breaks_log(10),
    #                 labels=trans_format("log10", math_format(10^.x)))+
  theme_bw()+
  theme(axis.title.x = element_text(face='bold', size=14),
        axis.title.y = element_text(face='bold', size=14),
        axis.text.x = element_text(size=12),
        axis.text.y = element_text(size=12)) + theme_minimal()

suppfig2
```

```
file.dir <- paste(getwd(), sep="")
file.name <- paste("/SuppFig2_hitc_dist_", Sys.Date(), ".png", sep="")
file.path <- paste(file.dir, file.name, sep="")
dir.create(path=file.dir, showWarnings = FALSE, recursive = TRUE)
png(width=4000, height=3000, res=600)
suppfig2
dev.off()
```

## 2.2 Flipped Hit Calls

## Flipped Hit Calls

### 2.2.1 Flip Direction

Flipped hit calls can be examined between versions. Deleted endpoints
in v4.0 were mapped to the component level to assess overall change.
Flip directions include: AA (active in both v3.5 and v4.0), AI (active
in v3.5, but inactive in v4.0), II (inactive in both v3.5 and v4.0), or
IA (inactive in v3.5, but active in v4.0). II and AA represent no change
in hit call determination for 88.6% and 9.6% of cases respectively, or
98.2% combined.

```
#-----------------------------------------------------------------------------------#
# DATABASE COMPARISON FOR FLIPPED HITS 
#-----------------------------------------------------------------------------------#
agg_flip <- potency %>% 
  filter(!aenm_v4.0=="DELETED") %>% #filter to remove duplicate counts
  group_by(flip_dir)  %>%
  count() %>% 
  ungroup() %>% 
  mutate(perc = `n` / sum(`n`)) %>% 
  arrange(perc) %>%
  mutate(labels = scales::percent(perc))
```

```
fig4b <- ggplot(agg_flip, aes(x = "", y = perc, fill = flip_dir)) +
  geom_bar(stat="identity", color="black") +
  ggrepel::geom_label_repel(aes(label = labels),
                            position = position_stack(vjust = .5),
                            show.legend = FALSE, fontface='bold', color='black', size=5) +
 #scale_fill_manual(values=c("#C87A8A",  "#9189C7", "#2A9EB5", "#A29048")) +
 scale_fill_manual(values=c('#2A788EFF', '#FDE725FF', '#414487FF','#7AD151FF'))+
  guides(fill = guide_legend(title = "Flip Direction")) +
  labs(y = "proportion") +
  coord_polar(theta = "y") + theme_void()

fig4b
```

### 2.2.2 Flag Analysis

v3.5 flags may provide insight into flip direction. For this
analysis, all v3.5 endpoints were considered and compared to the kept
v4.0 hit call. Number of flags in relation to flip direction was
examined. Compared to AA and II flip directions (no change between v3.5
and v4.0), IA and AI have the most diverse presence of flags. About 50%
of IA endpoint-samples have 1 or more flags whereas 75% of AI
endpoint-samples have 2 or more flags.

The type of flag can also be considered. The largest proportion of
flags for each flip direction are detailed below:

- AA primary flags are efficacy.50 (efficacy values less than 50%)
  and singlept.hit.high (single point active with activities only at the
  highest concentration)
- AI primary flags are efficacy.50, border hit (active with
  borderline activity), overfit.hit (active that would be changed with
  aicc recalculation considering n), and singlept.hit.mid (single point
  active with activities not at the highest concentration)
- IA primary flag is noise
- II primary flags are multipoint.neg (inactive with multiple
  medians above baseline), border.miss (inactive with borderline
  activity), and noise

Updated flagging logic will be implemented in tcpl for InvitroDB
v4.1. Complete descriptions of flags are included in the mc6\_methods
table in InvitroDB.

### 2.2.3 Cutoff versus Top Analysis

The kernel density of fold difference of the endpoint-sample top
compared to cutoff can be examined by flip direction. Vertical lines
corresponding to 1 fold and 1.5 fold difference of top/cutoff were
added. II flip direction was excluded since density distribution always
falls below 1.

```
#tcplConf(user="_dataminer", pass="pass", db="prod_internal_invitrodb_v4_0", drvr="MySQL", host="ccte-mysql-res.epa.gov")
#mc5 <- tcplPrepOtpt(tcplLoadData(lvl=5, type='mc',add.fld=TRUE))
#save(mc5, mc5_top, file="invitrodb_v4_0_top_over_cutoff.RData") 

load(file="invitrodb_v4_0_top_over_cutoff.RData") # load invitrodb v4.0 mc5 .Rdata including top_over_cutoff (mc5_top)
# merge mc5 top_over_cutoff with 'potency' table
mc5_top2 <- as.data.table(merge(potency, mc5_top, by=c('spid','aeid'), all.x=TRUE))
mc5_top3 <- mc5_top2[!aenm_v4.0=="DELETED",]
mc5_top3$act_flip <- as.factor(mc5_top3$act_flip)

supp3<- ggplot()+
  geom_density(data=mc5_top3[!flip_dir %in% "II",], 
               aes(x=top_over_cutoff, fill=flip_dir), alpha=0.25)+
  theme(text = element_text(size = 14, face="bold"))+
  xlim(-1,7)+
  geom_vline(xintercept=1.5, linetype='dashed',color='black')+
  geom_vline(xintercept=1, linetype='dashed',color='black')+
  scale_fill_manual(values=c("#4B0055", "#009B95", "#FDE333")) +
  guides(fill = guide_legend(title = "Flip Direction")) +
  annotate(geom="text", x=1.4, y=2, label="1.5x fold difference", color="black", angle=90)+
  annotate(geom="text", x=.8, y=2, label="1x fold difference", color="black", angle=90)+
  labs(y = "Density of Endpoint-samples in v4.0", x='Top/cutoff Fold Difference') + theme_minimal()

supp3
```

```
file.dir <- paste(getwd(), sep="")
file.name <- paste("/SuppFig3_top_coff_diff", Sys.Date(), ".png", sep="")
file.path <- paste(file.dir, file.name, sep="")
dir.create(path=file.dir, showWarnings = FALSE, recursive = TRUE)
png(width=5000, height=3000, res=600)
supp3
dev.off()
```

## 2.3 Winning Model Selection

## Winning Model Selection

Winning model selection between versions can be visualized. This does
not distinguish between positive and negative hit calls. In v3.5, a hit
call of -1 correspond to a “NA” model fitting. These are
concentration-response series that tcpl was unable to fit, usually due
to limited number of concentrations tested. In v3.5, there were 428 “-1”
hit calls. In v4.0, these series may appear fit in model “none” with a
hit call of 0, therefore inactive. In the new tcpl paradigm, constant
model can never win.

Models included are: “hill”, “gnls”(gain-loss), “cnst”(constant),
“pow”(power), “poly1”(polynomial-1: linear), “poly2”(polynomial-2:
quadratic), “exp2”(exponential-2), “exp3”(exponential-3),
“exp4”(exponential-4), “exp5”(exponential-5), NA (unable to fit in
v3.5), and “none”(unable to fit in v4.0).

```
modl_agg_v3.5 <- potency %>% 
  group_by(modl_v3.5) %>% 
  filter(!is.na(modl_v3.5)) %>% 
  count() %>% 
  ungroup() %>% 
  mutate(perc = `n` / sum(`n`)) %>% 
  arrange(perc) %>%
  mutate(labels = scales::percent(perc))
modl_agg_v3.5$version <- "v3.5"
setnames(modl_agg_v3.5, "modl_v3.5", "modl")

modl_agg_v4.0 <- potency %>% 
  filter(!aenm_v4.0=="DELETED") %>% 
  group_by(modl_v4.0) %>% 
  count() %>% 
  ungroup() %>% 
  mutate(perc = `n` / sum(`n`)) %>% 
  arrange(perc) %>%
  mutate(labels = scales::percent(perc))
modl_agg_v4.0$version <- "v4.0"
setnames(modl_agg_v4.0, "modl_v4.0", "modl")

modl_agg_comp<- rbind(modl_agg_v4.0, modl_agg_v3.5)

modl_agg_comp$modl<-factor(modl_agg_comp$modl, levels = c("hill", "gnls", "cnst", "pow", "poly1", "poly2", "exp2", "exp3", "exp4", "exp5", "none")) 

ggplot(modl_agg_comp, aes(x = version, y= n, fill = modl)) + geom_bar(colour="black", stat = "identity", position = position_fill(reverse = TRUE)) + 
  #scale_fill_manual(values=c("#C87A8A","#BC8463", "#AF8B52" , "#7B9B4F", "#45A271", "#00A396", "#2A9EB5", "#7591C6","#A782C3", "#B37EBE", "#C578A8", "#C578A6")) +
  scale_fill_manual(values=c("#FDE333", "#C8DF32", "#AF8B52", "#3AC96D", "#00B983", "#00A691", "#009097", "#007896", "#005F8E", "#30437F","#45256B", "#4B0055")) +
  guides(fill = guide_legend(title = "Winning Model")) +
  theme(axis.text.x = element_text(angle = 65, 
                                   vjust = 1, 
                                   hjust = 1), text=element_text(size=20),
        plot.title = element_text(hjust = 0.5, face = "bold"),
        legend.key.size = unit(1, 'cm'), #change legend key size
        legend.key.height = unit(1, 'cm'), #change legend key height
        legend.key.width = unit(1, 'cm'), #change legend key width
        legend.title = element_text(size=20), #change legend title font size
        legend.text = element_text(size=20)) + #change legend text font size
  labs(y = "Endpoint-sample Proportion", x="Version") + theme_minimal()
```

## 2.4 Winning Model Selection, Active Hits Only

## Winning Model Selection, Active Hits Only

This data was filtered to remove endpoint-samples that were inactive
between both versions. With the inactive-constant hits removed, hill and
gain-loss were only options in v3.5. In v4.0, new tcplFit2 models were
selected for active fits in addition to hill and gain-Loss. Models
included: “hill”, “gnls”(Gain-Loss), “pow”(power), “poly1”(polynomial-1:
linear), “poly2”(polynomial-2: quadratic), “exp2”(exponential-2),
“exp3”(exponential-3), “exp4”(exponential-4), “exp5”(exponential-5).

85% of active hits were hill and 15% were gnls in v3.5. The winning
model selection for active hits in v4.0 breaks down as: 20.63% exp5,
18.22% pow, 14.13% hill, 14.11% exp2, 13.56% poly1, 10.42% exp4, 5.50%
gnls, 2.86% exp3, and 0.55% poly2.

```
# subset to examine only positive hits
modl_active <- potency %>% 
  filter(!flip_dir=="II") %>%
  filter(!aenm_v4.0=="DELETED")

modl_active_v3.5 <- modl_active %>% 
  filter(!act_v3.5=="inactive") %>% #remove inactives that could not be modeled 
  group_by(modl_v3.5) %>% 
  count() %>% 
  ungroup() %>% 
  mutate(perc = `n` / sum(`n`)) %>% 
  arrange(perc) %>%
  mutate(labels = scales::percent(perc))
modl_active_v3.5$version <- "v3.5"
setnames(modl_active_v3.5, "modl_v3.5", "modl")

modl_active_v4.0 <- modl_active %>% 
  group_by(modl_v4.0) %>% 
  count() %>% 
  ungroup() %>% 
  mutate(perc = `n` / sum(`n`)) %>% 
  arrange(perc) %>%
  mutate(labels = scales::percent(perc))
modl_active_v4.0$version <- "v4.0"
setnames(modl_active_v4.0, "modl_v4.0", "modl")

modl_active_comp<- rbind(modl_active_v4.0, modl_active_v3.5)
modl_active_comp$modl <- factor(modl_active_comp$modl, levels = c("hill", "gnls", "pow", "poly1", "poly2", "exp2", "exp3", "exp4", "exp5"))

fig5a <- ggplot(modl_active_comp, aes(x = version, y= n, fill = modl)) + 
  geom_bar(colour="black", stat = "identity", position = position_fill(reverse = TRUE)) + 
  scale_fill_manual(values=c("#FDE333", "#B2DC3C", "#53CC67", "#00B785", "#009B95" ,"#007B97", "#00588B", "#403173" ,"#4B0055")) +
  guides(fill = guide_legend(title = "Winning Model")) +
    theme(axis.text.x = element_text(angle = 65, 
                                   vjust = 1, 
                                   hjust = 1), text=element_text(size=20),
        plot.title = element_text(hjust = 0.5, face = "bold"),
        legend.key.size = unit(2, 'cm'), #change legend key size
        legend.key.height = unit(2, 'cm'), #change legend key height
        legend.key.width = unit(2, 'cm'), #change legend key width
        legend.title = element_text(size=30), #change legend title font size
        legend.text = element_text(size=30)) + #change legend text font size
  labs(y = "Endpoint-sample Proportion", x="Version") + theme_minimal()
```

This v3.5 winning model selection can be stratified by hill and gnls
to understand how v4.0 selection compares.

For the 85% of active hits that were hill in v3.5, the winning model
selection in v4.0 breaks down as: 20.25% exp5, 19.5% pow, 16.98% exp2,
16.87% hill, 12.05% poly1, 9.72% exp4, 3.48% exp3, 0.64% poly2, and
0.51% gnls.

For the 15% of active hits that were gnls in v3.5, the winning model
selection in v4.0 breaks down as: 32.66% gnls, 22.18% poly1, 21.94%
exp5, 15.03% exp4, 2.93% hill, 2.21% exp2, 1.59% pow, 0.79% exp3, and
0.04% poly1.

```
# subset to v3.5 hill model hits
modl_active <- potency %>% 
  filter(!flip_dir=="II") %>%
  filter(!aenm_v4.0=="DELETED") %>%
  filter(modl_v3.5=="hill")

modl_active_v3.5 <- modl_active %>% 
  filter(!act_v3.5=="inactive") %>%
  group_by(modl_v3.5) %>% 
  count() %>% 
  ungroup() %>% 
  mutate(perc = `n` / sum(`n`)) %>% 
  arrange(perc) %>%
  mutate(labels = scales::percent(perc))
modl_active_v3.5$version <- "v3.5"
setnames(modl_active_v3.5, "modl_v3.5", "modl")

modl_active_v4.0 <- modl_active %>% 
  group_by(modl_v4.0) %>% 
  count() %>% 
  ungroup() %>% 
  mutate(perc = `n` / sum(`n`)) %>% 
  arrange(perc) %>%
  mutate(labels = scales::percent(perc))
modl_active_v4.0$version <- "v4.0"
setnames(modl_active_v4.0, "modl_v4.0", "modl")

modl_active_comp<- rbind(modl_active_v4.0, modl_active_v3.5)
modl_active_comp$modl <- factor(modl_active_comp$modl, levels = c("hill", "gnls", "pow", "poly1", "poly2", "exp2", "exp3", "exp4", "exp5"))

hill_modl_agg_actives <- ggplot(modl_active_comp, aes(x = version, y= n, fill = modl)) + geom_bar(colour="black", stat = "identity", position = position_fill(reverse = TRUE)) + 
  #scale_fill_manual(values=c("#C87A8A","#BC8463",  "#7B9B4F", "#45A271", "#00A396", "#2A9EB5", "#7591C6","#A782C3", "#C279AD")) +
  scale_fill_manual(values=c("#FDE333", "#B2DC3C", "#53CC67", "#00B785", "#009B95" ,"#007B97", "#00588B", "#403173" ,"#4B0055")) +
  guides(fill = "none") +
  labs(y = "Endpoint-sample Proportion", x="Version") + theme_minimal()

# subset to v3.5 gnls model hits
modl_active <- potency %>% 
  filter(!flip_dir=="II") %>%
  filter(!aenm_v4.0=="DELETED") %>%
  filter(modl_v3.5=="gnls")

modl_active_v3.5 <- modl_active %>% 
  filter(!act_v3.5=="inactive") %>%
  group_by(modl_v3.5) %>% 
  count() %>% 
  ungroup() %>% 
  mutate(perc = `n` / sum(`n`)) %>% 
  arrange(perc) %>%
  mutate(labels = scales::percent(perc))
modl_active_v3.5$version <- "v3.5"
setnames(modl_active_v3.5, "modl_v3.5", "modl")

modl_active_v4.0 <- modl_active %>% 
  group_by(modl_v4.0) %>% 
  count() %>% 
  ungroup() %>% 
  mutate(perc = `n` / sum(`n`)) %>% 
  arrange(perc) %>%
  mutate(labels = scales::percent(perc))
modl_active_v4.0$version <- "v4.0"
setnames(modl_active_v4.0, "modl_v4.0", "modl")

modl_active_comp<- rbind(modl_active_v4.0, modl_active_v3.5)
modl_active_comp$modl <- factor(modl_active_comp$modl, levels = c("hill", "gnls", "pow", "poly1", "poly2", "exp2", "exp3", "exp4", "exp5"))

gnls_modl_agg_actives <- ggplot(modl_active_comp, aes(x = version, y= n, fill = modl)) + geom_bar(colour="black", stat = "identity", position = position_fill(reverse = TRUE)) + 
  #scale_fill_manual(values=c("#C87A8A","#BC8463",  "#7B9B4F", "#45A271", "#00A396", "#2A9EB5", "#7591C6","#A782C3", "#C279AD")) +
  scale_fill_manual(values=c("#FDE333", "#B2DC3C", "#53CC67", "#00B785", "#009B95" ,"#007B97", "#00588B", "#403173" ,"#4B0055")) +
  guides() +
  labs(y = "Endpoint-sample Proportion", x="Version") + theme_minimal()

fig5b <- hill_modl_agg_actives + gnls_modl_agg_actives
```

```
fig5_all <- ggdraw()+
  draw_plot(fig5a, 0, 0.5,1,0.5)+
  draw_plot(fig5b, 0,   0,1,0.5)+
  draw_plot_label(c('A','B'), c(0, 0), c(1, 0.5), size=16)
fig5_all
```

```
file.dir <- paste(getwd(), sep="")
file.name <- paste("/Fig5_modl_flip_", Sys.Date(), ".png", sep="")
file.path <- paste(file.dir, file.name, sep="")
dir.create(path=file.dir, showWarnings = FALSE, recursive = TRUE)
png(width=5000, height=5000, res=600)
fig5_all
dev.off()
```

## 2.5 Log10 Potency

## Log10 Potency

Point of departure potency estimates were based on modeled active
concentration series, including ACC (activity concentration at cutoff),
AC10 (activity concentration at 10% of maximal response), and AC50
(activity concentration at 50% of maximal response). tcplFit2 modeling
has incorporated new potency and uncertainty estimates, based on the
BMDExpress transcriptomics dose-response modeling software, which
includes benchmark dose (BMD) estimation (that is the dose at which a
defined Benchmark Response (BMR) level occurs). The BMR is defined to be
one standard deviation shift from the baseline response for the
endpoint(s) of interest. Here, the baseline response is estimated using
the two lowest concentrations across all tested chemicals. Potency
analysis examines \(log\_{10}\) potency
values, or the logged tested concentration in uM.

### 2.5.1 Log10 Potency Distribution

To examine the potency distributions, data was first filtered to
remove endpoint-samples that were inactive between both versions.
Inactives in v3.5 (hitc=0) do not have any associated potency values
(NA) therefore they cannot be compared to v4.0 values.

```
#subset remove rows where always inactive between versions since these often do not have potency values
potency <- as.data.table(potency)
potency_active <-  potency[!flip_dir %in% c("II","IA"),] 
potency_active <- potency_active %>% 
  filter(!aenm_v4.0=="DELETED")

potency_agg_comp_long <- as.data.table(melt(potency_active,
                                  # ID variables - all the variables to keep but not split apart on
                                  id.vars=c("spid" ,"asnm", "acid" , "acnm_v4.0" , "aeid",  "aenm_v3.5", "aenm_v4.0",  "chnm", "flip_dir"),
                                  # The source columns
                                  measure.vars=c( "bmd_v4.0","ac50_v3.5", "ac50_v4.0", "acc_v3.5", "acc_v4.0", "ac10_v3.5", "ac10_v4.0"),
                                  # Name of the destination column that will identify the original
                                  # column that the measurement came from
                                  variable.name="potency",
                                  value.name="value"))

potency_agg_comp_long$version <- "v4.0"
potency_agg_comp_long[potency %in% c("ac50_v3.5","ac10_v3.5","acc_v3.5"), version:= "v3.5"]
potency_agg_comp_long[potency %in% c("ac50_v3.5","ac50_v4.0"), potency:= "ac50"]
potency_agg_comp_long[potency %in% c("ac10_v3.5","ac10_v4.0"), potency:= "ac10"]
potency_agg_comp_long[potency %in% c("acc_v3.5","acc_v4.0"), potency:= "acc"]
potency_agg_comp_long[potency %in% c("bmd_v4.0"), potency:= "bmd"]

#filter to remove instances if NA in either one version (limit to values used in stat test comparison)
potency_agg_comp_long <- potency_agg_comp_long %>% 
  group_by(spid, aeid, potency) %>% 
    filter(!any(is.na(value))) 

ggplot(data = potency_agg_comp_long, aes(x = value, y = potency, fill=version, na.rm=TRUE)) +
  geom_boxplot(outlier.alpha = 0.2) + theme_minimal() +
  theme(strip.background = element_blank(),
        strip.text.y = element_text(angle=360)) +
  xlim(-10,10) +
  scale_fill_manual(values = c("gray", "white")) +
  labs(y= NULL, x = "Log 10 Potency (uM)") +
  guides(fill = guide_legend(title = "Version"))
```

Flip direction in relation to each of these potency values can be
considered.

```
ggplot(data = potency_agg_comp_long, aes(x = value, y = potency, color=flip_dir, fill=version, na.rm=TRUE)) +
  geom_boxplot(outlier.alpha = 0.2) + theme_minimal() +
  theme(strip.background = element_blank(),
        strip.text.y = element_text(angle=360)) +
  xlim(-10,10)+ 
  scale_fill_manual(values = c("gray", "white")) +
  scale_color_manual(values=c("#4B0055","#53CC67")) +
  labs(y= NULL, x = "Log 10 Potency (uM)") +
  guides(color = guide_legend(title = "Flip Direction"), fill = guide_legend(title = "Version"))
```

The following scatterplot compares \(log\_{10}\) potency values between the two
database versions. Values along the diagonal dashed line indicate
concordant (i.e. equivalent) \(log\_{10}\) potency values. Out of the \(log\_{10}\) potency values the most apparent
and systematic discordance is observed between the ACC values in the AI
flip direction. In the AC50 values for the AI flip direction we also
observe a relatively high level of discordance between potency
estimates, but there is not a consistent pattern in the shift.

```
ggplot(data = potency_active, aes(x = ac50_v4.0, y = ac50_v3.5, color=flip_dir, na.rm=TRUE)) +
  geom_point(alpha = 2/10) + theme_bw() +
  theme(strip.background = element_blank(),
        strip.text.y = element_text(angle=360)) +
  geom_abline(slope = 1,intercept = 0,col = "gray")+
  facet_grid(rows=vars(flip_dir)) +
  scale_color_manual(values=c( "#009B95", "#4B0055")) +
  labs(x = "Log10 AC50 (uM) in v4.0", y= "Log10 AC50 (uM) in v3.5") +
  guides(color = guide_legend(title = "Flip Direction"))
```

```
ggplot(data = potency_active, aes(x = acc_v4.0, y = acc_v3.5, color=flip_dir, na.rm=TRUE)) +
  geom_point(alpha = 2/10) + theme_bw() +
  theme(strip.background = element_blank(),
        strip.text.y = element_text(angle=360)) +
  geom_abline(slope = 1,intercept = 0,col = "gray")+
  facet_grid(rows=vars(flip_dir)) +
  scale_color_manual(values=c("#009B95", "#4B0055")) +
  labs(x = "Log10 ACC (uM) in v4.0", y= "Log10 ACC (uM) in v3.5")+
  guides(fill = guide_legend(title = "Flip Direction"))
```

```
# Create a dataframe where the potency estimate values
# are put into respective database version variables.
potency_agg_comp_vers <- reshape2::dcast(potency_agg_comp_long,spid+asnm+acid+aeid+chnm+flip_dir+potency~version)
```

```
## All potency estimates comparisons
# Scatterplot comparing potency estimates between databases
# within the same potency estimate

# filter the data
potency_agg_comp_vers %>%
  dplyr::filter(.,potency != "bmd") %>% 
  # start the plotting
  ggplot(.,aes(x = v4.0,y = v3.5,col = flip_dir))+
  geom_point(alpha = 0.2)+
  scale_color_manual(values=c( "#009B95", "#4B0055")) +
  # add reference lines
  geom_abline(slope = 1,intercept = 0,col = "gray")+
  geom_abline(slope = 1,intercept = c(-0.5,0.5),
              col = "purple",linetype = "dotted")+
  geom_abline(slope = 1,intercept = c(-2.5,2.5),
              col = "blue",linetype = "dashed")+
  # layout of the plots
  facet_grid(flip_dir ~ potency)+
  theme_bw() +
  theme(strip.background = element_blank(),
        strip.text.y = element_text(angle=360))+
  labs(x = "Log10 Potency Estimate (uM) in v4.0",
       y= "Log10 Potency Estimate (uM) in v3.5")+
  guides(col = guide_legend(title = "Flip Direction"))
```

### 2.5.2 Magnitude of Log10 Potency Change

Magnitude of potency change was assessed by calculating the \(log\_{10}\) absolute difference in point of
departure potency estimates between v3.5 and v4.0, \(log\_{10}(|pod\_{v4.0} - pod\_{v3.5}|)\),
where \(pod\_{v4.0}\) and \(pod\_{v4.0}\) are in the unlogged-space for
experimental concentrations in uM.

Though there are quite a few outliers, as seen in the first boxplot
below, this is still a fraction of the overall majority of the data
existing in the database. Overall, the majority of the differences in
potency estimates between v3.5 and v4.0 are within a factor of 10
(i.e. 1 \(log\_{10}\) unit) as shown by
the vertical blue dotted lines and the boxes falling within that
interval.

```
#subset remove rows where always inactive between versions since these often do not have potency values
potency_agg_change_long <- melt(potency_active,
                                  # ID variables - all the variables to keep but not split apart on
                                  id.vars=c("spid" ,"asnm", "acid" , "acnm_v4.0" , "aeid",  "aenm_v3.5", "aenm_v4.0",  "chnm"),
                                  # The source columns
                                  measure.vars=c("ac50_change", "acc_change", "ac10_change"),
                                  # Name of the destination column that will identify the oiginal
                                  # column that the measurement came from
                                  variable.name="change",
                                  value.name="value")

ggplot(data = potency_agg_change_long, aes(x = value, y = change, na.rm=TRUE)) +
  geom_boxplot(outlier.alpha = 0.2) +
  geom_vline(xintercept = c(-0.5,0.5),lty = "dashed",col = "purple")+ # 0.5 log-unit
  theme_minimal() +
  theme(strip.background = element_blank(),
        strip.text.y = element_text(angle=360)) +
  xlim(-20, 20) +
  labs(y= NULL, x = "Log10 Potency Change (uM)")
```

```
ggplot(data = potency_agg_change_long, aes(x = value, y = change, na.rm=TRUE)) +
  geom_boxplot(outlier.shape = NA) +
  geom_vline(xintercept = c(-0.5,0.5),lty = "dashed",col = "purple")+ # 0.5 log-unit
  theme_minimal() +
  theme(strip.background = element_blank(),
        strip.text.y = element_text(angle=360)) +
  xlim(-3, 3) +
  labs(y= NULL, x = "Log10 Potency Change (uM)")
```

## 2.6 Log10 Potency - AA only

## Log10 Potency - AA only

Potency analysis can be repeated to examine Log10 potency values, or
the logged tested concentration in uM.

Point of departure potency estimates were based on modeled active
concentration series, including ACC (activity concentration at cutoff),
AC10 (activity concentration at 10% of maximal response), and AC50
(activity concentration at 50% of maximal response). tcplFit2 modeling
includes new potency and uncertainty estimates, based on the BMDExpress
software, that is the benchmark dose (BMD) as defined by the Benchmark
Response (BMR) level. BMR is set to a one standard deviation shift from
the baseline response for the endpoint of interest. Here, the baseline
response is estimated using the two lowest concentrations across all
tested chemicals. The potency analysis examines \(log\_{10}\) potency values, or the
log-transformed experimental concentrations in uM.

### 2.6.1 Log10 Potency Distribution

To examine the \(log\_{10}\) potency
distributions, data was first filtered to remove endpoint-samples that
were not active in both versions.

Compared to \(log\_{10}\) potency
distributions which examine both AA and AI cases, v4.0 distributions of
ACC and BMD show far fewer extremely large values when only examining AA
cases. AC10 and AC50 distributions appear quite similar between
versions, while ACC distributions differ somewhat in that v3.5 spread
includes more extreme values on the larger end than that of v4.0.

```
#subset remove rows where always inactive between versions since these often do not have potency values
potency <- as.data.table(potency)
potency_active_AA <-  potency[!flip_dir %in% c("II","IA","AI"),] 
potency_active_AA <- potency_active_AA %>% 
  filter(!aenm_v4.0=="DELETED")

potency_agg_comp_long <- as.data.table(melt(potency_active_AA,
                                  # ID variables - all the variables to keep but not split apart on
                                  id.vars=c("spid" ,"asnm", "acid" , "acnm_v4.0" , "aeid",  "aenm_v3.5", "aenm_v4.0",  "chnm"),
                                  # The source columns
                                  measure.vars=c( "bmd_v4.0","ac50_v3.5", "ac50_v4.0", "acc_v3.5", "acc_v4.0", "ac10_v3.5", "ac10_v4.0"),
                                  # Name of the destination column that will identify the original
                                  # column that the measurement came from
                                  variable.name="potency",
                                  value.name="value"))

potency_agg_comp_long$version <- "v4.0"
potency_agg_comp_long[potency %in% c("ac50_v3.5","ac10_v3.5","acc_v3.5"), version:= "v3.5"]
potency_agg_comp_long[potency %in% c("ac50_v3.5","ac50_v4.0"), potency:= "ac50"]
potency_agg_comp_long[potency %in% c("ac10_v3.5","ac10_v4.0"), potency:= "ac10"]
potency_agg_comp_long[potency %in% c("acc_v3.5","acc_v4.0"), potency:= "acc"]
potency_agg_comp_long[potency %in% c("bmd_v4.0"), potency:= "bmd"]

#filter to remove instances if NA in either one version (limit to values used in stat test comparison)
potency_agg_comp_long <- potency_agg_comp_long %>% 
  group_by(spid, aeid, potency) %>% 
    filter(!any(is.na(value))) 

ggplot(data = potency_agg_comp_long, aes(x = value, y = potency, fill=version, na.rm=TRUE)) +
  geom_boxplot(outlier.alpha = 0.2) + theme_minimal() +
  theme(strip.background = element_blank(),
        strip.text.y = element_text(angle=360)) +
  xlim(-10,10) +
  scale_fill_manual(values = c("gray", "white")) +
  labs(y= NULL, x = "Log 10 Potency (uM)") +
  guides(fill = guide_legend(title = "Version"))
```

We can evaluate a linear regression model of AC50 values between
versions and plot values in a scatterplot:

```
ac50.model <- lm(ac50_v4.0 ~ ac50_v3.5, data = potency_active_AA)
summary(ac50.model)
```

```
## 
## Call:
## lm(formula = ac50_v4.0 ~ ac50_v3.5, data = potency_active_AA)
## 
## Residuals:
##     Min      1Q  Median      3Q     Max 
## -4.9386 -0.0259  0.0219  0.0563  6.7445 
## 
## Coefficients:
##              Estimate Std. Error  t value Pr(>|t|)    
## (Intercept) 0.0059526  0.0006653    8.947   <2e-16 ***
## ac50_v3.5   0.9745874  0.0004614 2112.315   <2e-16 ***
## ---
## Signif. codes:  0 '***' 0.001 '**' 0.01 '*' 0.05 '.' 0.1 ' ' 1
## 
## Residual standard error: 0.1932 on 248377 degrees of freedom
##   (60654 observations deleted due to missingness)
## Multiple R-squared:  0.9473, Adjusted R-squared:  0.9473 
## F-statistic: 4.462e+06 on 1 and 248377 DF,  p-value: < 2.2e-16
```

Mean square error is 0.0373402

```
ggplot(data = potency_active_AA, aes(x = ac50_v4.0, y = ac50_v3.5, na.rm=TRUE)) +
  geom_point(alpha = 0.2) +
  theme_bw() +
  theme(strip.background = element_blank(),
        strip.text.y = element_text(angle=360)) +
  labs(x = "Log10 AC50 (uM) in v4.0", y= "Log10 AC50 (uM) in v3.5") +
  xlim(-3, 3) +
  ylim(-3,3) +
  geom_abline(slope=1, intercept=0,col = "gray") +
  geom_abline(slope=1, intercept=c(-0.5,0.5),
              col = "purple",linetype="dashed") +
  theme_bw()+
  theme(axis.title.x = element_text(face='bold', size=14),
        axis.title.y = element_text(face='bold', size=14),
        axis.text.x = element_text(size=12),
        axis.text.y = element_text(size=12))+
  annotate('text', x=-2, y=2, face = 'bold', size = 5, label='Adj R2 = 0.9473')
```

We can evaluate a linear regression model of ACC values between
versions and plot values in a scatterplot:

```
acc.model <- lm(acc_v4.0 ~ acc_v3.5, data = potency_active_AA)
summary(acc.model)
```

```
## 
## Call:
## lm(formula = acc_v4.0 ~ acc_v3.5, data = potency_active_AA)
## 
## Residuals:
##     Min      1Q  Median      3Q     Max 
## -4.7352 -0.0340 -0.0150  0.0355  6.9467 
## 
## Coefficients:
##              Estimate Std. Error t value Pr(>|t|)    
## (Intercept) 0.0385577  0.0004703   81.99   <2e-16 ***
## acc_v3.5    0.9916184  0.0003391 2923.89   <2e-16 ***
## ---
## Signif. codes:  0 '***' 0.001 '**' 0.01 '*' 0.05 '.' 0.1 ' ' 1
## 
## Residual standard error: 0.155 on 248376 degrees of freedom
##   (60655 observations deleted due to missingness)
## Multiple R-squared:  0.9718, Adjusted R-squared:  0.9718 
## F-statistic: 8.549e+06 on 1 and 248376 DF,  p-value: < 2.2e-16
```

Mean square error is 0.0240138

```
ggplot(data = potency_active_AA, aes(x = acc_v4.0, y = acc_v3.5, na.rm=TRUE)) +
  geom_point(alpha = 2/10) + theme_bw() +
  theme(strip.background = element_blank(),
        strip.text.y = element_text(angle=360)) +
  labs(x = "Log10 ACC (uM) in v4.0", y= "Log10 ACC (uM) in v3.5") +
  xlim(-3, 3) +
  ylim(-3,3) +
  geom_abline(slope=1, intercept=0,col = "gray") +
  geom_abline(slope=1, intercept=c(-0.5,0.5),
              col = "purple",linetype="dashed") +
  theme_bw()+
  theme(axis.title.x = element_text(face='bold', size=14),
        axis.title.y = element_text(face='bold', size=14),
        axis.text.x = element_text(size=12),
        axis.text.y = element_text(size=12))+
  annotate('text', x=-2, y=2, face = 'bold', size = 5, label='Adj R2 = 0.9718')
```

```
fig6a <- potency_agg_comp_vers %>%
  dplyr::filter(.,potency != "bmd") %>% 
  dplyr::filter(.,flip_dir == "AA") %>%
  # start the plotting
  ggplot(.,aes(x = v4.0,y = v3.5))+
  geom_point(alpha = 0.2)+
  # add reference lines
  geom_abline(slope = 1,intercept = 0,col = "gray")+
  geom_abline(slope = 1,intercept = c(-0.5,0.5),
              col = "purple",linetype = "dotted")+
  geom_abline(slope = 1,intercept = c(-2.5,2.5),
              col = "blue",linetype = "dashed")+
  # layout of the plots
  facet_wrap(facets = "potency",ncol = 2)+
  theme_bw() +
  theme(strip.background = element_blank(),
        strip.text.y = element_text(angle=360))+
  scale_x_continuous(breaks = seq(-10,10,2.5))+
  scale_y_continuous(breaks = seq(-10,10,2.5))+
  labs(x = "Log10 Potency Estimate (uM) in v4.0",
       y= "Log10 Potency Estimate (uM) in v3.5")+
  guides(col = guide_legend(title = "Flip Direction")) 
# print the figure
fig6a
```

### 2.6.2 Magnitude of Log10 Potency Change

Magnitude of potency change was assessed by calculating the log 10
absolute difference in point of departure potency estimates between v3.5
and v4.0, where potency value reflects the unlogged tested concentration
in uM.

Majority fall within .5log10 change for these potency estimates
across the database between versions, although there are outliers.

```
#subset remove rows where always inactive between versions since these often do not have potency values
potency_agg_change_long <- melt(potency_active_AA,
                                  # ID variables - all the variables to keep but not split apart on
                                  id.vars=c("spid" ,"asnm", "acid" , "acnm_v4.0" , "aeid",  "aenm_v3.5", "aenm_v4.0",  "chnm"),
                                  # The source columns
                                  measure.vars=c("ac50_change", "acc_change", "ac10_change"),
                                  # Name of the destination column that will identify the oiginal
                                  # column that the measurement came from
                                  variable.name="change",
                                  value.name="value")

ggplot(data = potency_agg_change_long, aes(x = value, y = change, na.rm=TRUE)) +
  geom_boxplot(outlier.alpha = 0.2) +
  geom_vline(xintercept = c(-0.5,0.5),col = "purple",
             linetype = "dashed") +
  theme_minimal() +
  theme(strip.background = element_blank(),
        strip.text.y = element_text(angle=360)) +
  # xlim(-20, 20) +
  labs(y= NULL, x = "Log10 Potency Change (uM)")
```

```
ggplot(data = potency_agg_change_long, aes(x = value, y = change, na.rm=TRUE)) +
  geom_boxplot(outlier.shape = NA) +
  geom_vline(xintercept = c(-0.5,0.5),col = "purple",
             linetype = "dashed") +
  theme_minimal() +
  theme(strip.background = element_blank(),
        strip.text.y = element_text(angle=360)) +
  xlim(-3, 3) +
  labs(y= NULL, x = "Log10 Potency Change (uM)")
```

Other metrics can be output to evaluate the difference:

```
Metric <- c("Mean", 
            "Median",
            "Standard Deviation")
AC50_Difference_in_log10_uM<- c(mean(potency_active_AA$ac50_change, na.rm = TRUE),
          median(potency_active_AA$ac50_change, na.rm = TRUE), 
          sd(potency_active_AA$ac50_change, na.rm = TRUE))
ACC_Difference_in_log10_uM<- c(mean(potency_active_AA$acc_change, na.rm = TRUE),
          median(potency_active_AA$acc_change, na.rm = TRUE), 
          sd(potency_active_AA$acc_change, na.rm = TRUE))
AC10_Difference_in_log10_uM<- c(mean(potency_active_AA$ac10_change, na.rm = TRUE),
          median(potency_active_AA$ac10_change, na.rm = TRUE), 
          sd(potency_active_AA$ac10_change, na.rm = TRUE))

Table <- data.frame(Metric, AC50_Difference_in_log10_uM, ACC_Difference_in_log10_uM, AC10_Difference_in_log10_uM)

datatable(Table,
          filter='top',
          options=list(pageLength = 15,searching=FALSE, autoWidth=FALSE,  scrollX=TRUE, initComplete = JS(
            "function(settings, json) {",
            "$('body').css({'font-family': 'Calibri'});",
            "}"
          )))
```

## 2.7 Root Mean Squared Deviation (RMSD) of Log10 Potency Change

This section is aimed at getting an overall estimate of how different
\(log\_{10}\) potency estimates are for
(a) a single potency estimates between the two databases (within
estimate) or (b) across the BMD and ACC within database version 4.0. The
root mean square difference (RMSD) gives us an idea of how “far apart”
potency estimates any by how much. See the equation below for the RMSD
calculation:

\[ RMSD =
\sqrt{\frac{\Sigma\_{i=1}^{n}(x\_i-y\_i)^2}{n}}\]

where \(x\_i\) and \(y\_i\) are the same potency estimate from
v3.5 and v4.0, respectively, \(i\)
indicates the ‘i-th’ observed assay endpoint and chemical pair, and
\(n\) is the total number of observed
pairs. (Function is defined in the following code chunk.)

For the within potency estimate comparisons we will focus on only the
v4.0 active cases and break down the RMSD by flip direction category.
Additionally, we explore whether there is a particular set of assays
that may have greater disparity in potency estimtes between database
versions.

For the BMD and ACC comparison, we evaluate the disparity of
estimates within the various version 4.0 database activity categories
then the RMSD within flip direction categories.

```
# x - is the previous version
# y - is the current version
rmsd <- function(x,y){
  # # check whether the length of the vectors being compared is different
  if(length(x)!=length(y)){
    stop("Length of x and y differ. Note elements should be matched pairs")
  }
  # obtain the difference between x and y
  d <- y - x
  # remove any cases where the difference is NA
  if(any(is.na(d))==TRUE){d <- d[-which(is.na(d))]}
  # obtain the root mean squared difference (RMSD)
  out <- sqrt(mean(d^2))
  # return the results
  return(out)
}
```

### 2.7.1 Within Potency Estimate (across DB version)

In this sub-section we will compare the individual potency estimates
between the two database versions, namely AC10, AC50, and ACC.

For most of the comparison groups the RMSD is close to 0 indicating
that the \(log\_{10}\) potency estimates
are relatively similar. However, as with the scatterplots shown
previously we observe that there is more disparity between potency
estimates for assay endpoint and chemical pairs for which the response
was considered active in v3.5 and inactive in v4.0 (AI flip direction).
This is especially apparent for the ACC potency estimate for AI flip
direction cases.

```
potency_active %>%
  dplyr::group_by(.,flip_dir) %>% 
  dplyr::summarise(.data = .,
                   RMSD_AC50 = rmsd(ac50_v3.5,ac50_v4.0),
                   RMSD_ACC = rmsd(acc_v3.5,acc_v4.0),
                   RMSD_AC10 = rmsd(ac10_v3.5,ac10_v4.0))
```

### 2.7.2 Mean Absolute Difference (Supplemental Statistic)

To get an understanding of the average difference between the potency
estimates of the two databases we can calculate the mean absolute
difference (MAD) between v3.5 and v4.0.

```
# x - is the previous version
# y - is the current version
mean_abs_diff <- function(x,y){
  # # check whether the length of the vectors being compared is different
  if(length(x)!=length(y)){
    stop("Length of x and y differ. Note elements should be matched pairs")
  }
  # obtain the difference between x and y
  d <- y - x
  # remove any cases where the difference is NA
  if(any(is.na(d))==TRUE){d <- d[-which(is.na(d))]}
  # obtain the root mean squared difference (RMSD)
  out <- mean(abs(d))
  # return the results
  return(out)
}
```

```
potency_active %>%
  dplyr::group_by(.,flip_dir) %>% 
  dplyr::summarise(.data = .,
                   MeanAD_AC50 = mean_abs_diff(ac50_v3.5,ac50_v4.0),
                   MeanAD_AC50 = mean_abs_diff(acc_v3.5,acc_v4.0),
                   MeanAD_AC50 = mean_abs_diff(ac10_v3.5,ac10_v4.0))
```

### 2.7.3 ACC to BMD (v4.0)

In this sub-section we will compare the BMD and ACC from the v4.0
database to evaluate the relationship between these two potency
estimates.

In the case of the ACC compared with the BMD estimate, from v4.0, we
see that the RMSD is higher for AA flip-direction cases compared to the
other ‘within potency estimate’ comparisons, which is to be expected
considering that the ACC and BMD are are inherently different potency
estimates.

```
# Activity counts for version 4.0
table(potency$act_v4.0,exclude = FALSE) %>% as.data.frame()
```

```
# RMSD within activity counts for v4.0
potency %>%
  dplyr::group_by(.,act_v4.0) %>% 
  dplyr::summarise(.data = .,
                   RMSD_BMD_ACC = rmsd(bmd_v4.0,acc_v4.0))
```

```
# RMSD for observations by their flip direction (actives only)
potency %>%
  dplyr::group_by(.,flip_dir) %>% 
  dplyr::summarise(.data = .,
                   RMSD_BMD_ACC = rmsd(bmd_v4.0,acc_v4.0))
```

### 2.7.4 Bootstrapped RMSD Estimates

To understand the uncertainty in the RMSD estimate for each of the
comparisons we perform a bootstrap calculation for 10,000 iterations. It
should be noted in this section we are only interested in observations
that are active in both v3.5 and v4.0 for all comparisons.

```
# data - dataset with all unapplicable observations removed (diff = NA)
# index - the randomly chosen observations for the bootstrap iteration
rmsd_boot <- function(data,index){
  tdat <- data[index,] # obtain the bootstrap resample
  out <- rmsd(tdat[,1],tdat[,2]) # estimate RMSD
  return(out)
}
```

For the within potency estimates (AC10, AC50, and ACC) we only obtain
the bootstrapped estimation of RMSD for the “AA” flip direction cases.
However, for the comparison between the BMD and ACC from version v4.0 we
consider all “actives” from the same database (i.e. IA and AA) which is
determined based on continuous hitcall greater than or equal to 0.9
(\(hitc \geq 0.9\)).

All of the RMSD boostrapped percentile confidence intervals are
fairly tight around the overall RMSD estimate. For the active chemical
endpoint pairs, then within potency estimate shows overall fairly high
concordance between the v3.5 and v4.0 values as the RMSD values are
fairly close to 0. Based on the RMSD estimates and the confidence
intervals ACC, AC50, followed by AC10 are most consistent across the two
databases for active to active cases (note listed in order of highest to
lowest concordance, respectively).

*Note: Running the following bootstrapped code chunks is
computationally intensive. Suggest running one chunk at a time if you
are a user replicating this analysis.*

```
## Number of Bootstrap Samples to Take
MyR <- 5000
## Common Seed to Set for Random Number Generation
set.seed(525)
MySeed <- sample(x = 101:1231,size = 4)
MySeed # print the seeds for each bootstrapping set
```

```
## [1]  802 1059 1160 1002
```

```
## AC50 Potency Estimation ##
set.seed(MySeed[1])
ac50_rmsd_boot <- potency_active %>% 
  dplyr::mutate(.,log10diff = ac50_v4.0 - ac50_v3.5) %>% 
  dplyr::filter(.,is.na(log10diff)==FALSE) %>% 
  dplyr::filter(.,flip_dir == "AA") %>%
  dplyr::select(.,c(ac50_v3.5,ac50_v4.0)) %>%
  data.frame() %>% 
  boot::boot(data = .,statistic = rmsd_boot,R = MyR)
# export data - large files
save(ac50_rmsd_boot,file = "ac50_rmsd_boot.RData")
# remove data from memory to allow for next set
rm(ac50_rmsd_boot)
```

```
## ACC Potency Estimation ##
set.seed(MySeed[2])
acc_rmsd_boot <- potency_active %>% 
  dplyr::mutate(.,log10diff = acc_v4.0 - acc_v3.5) %>% 
  dplyr::filter(.,is.na(log10diff)==FALSE) %>% 
  dplyr::filter(.,flip_dir == "AA") %>%
  dplyr::select(.,c(acc_v3.5,acc_v4.0)) %>%
  data.frame() %>% 
  boot::boot(data = .,statistic = rmsd_boot,R = MyR)
# export data - large files
save(acc_rmsd_boot,file = "acc_rmsd_boot.RData")
# remove data from memory to allow for next set
rm(acc_rmsd_boot)
```

```
## AC10 Potency Estimate ##
set.seed(MySeed[3])
ac10_rmsd_boot <- potency_active %>% 
  dplyr::mutate(.,log10diff = ac10_v4.0 - ac10_v3.5) %>% 
  dplyr::filter(.,is.na(log10diff)==FALSE) %>% 
  dplyr::filter(.,flip_dir == "AA") %>%
  dplyr::select(.,c(ac10_v3.5,ac10_v4.0)) %>%
  data.frame() %>% 
  boot::boot(data = .,statistic = rmsd_boot,R = MyR)
# export data - large files
save(ac10_rmsd_boot,file = "ac10_rmsd_boot.RData")
# remove data from memory to allow for next set
rm(ac10_rmsd_boot)
```

```
## BMD with ACC Potency Estimate ##
set.seed(MySeed[4])
bmd2acc_rmsd_boot <- potency %>% 
  dplyr::mutate(.,log10diff = bmd_v4.0 - acc_v4.0) %>% 
  dplyr::filter(.,is.na(log10diff)==FALSE) %>% 
  dplyr::filter(.,hitc_v4.0>=0.9) %>% 
  dplyr::select(.,c(acc_v4.0,bmd_v4.0)) %>%
  data.frame() %>% 
  boot::boot(data = .,statistic = rmsd_boot,R = MyR)
## Export Data ##
save(bmd2acc_rmsd_boot,file = "bmd2acc_rmsd_boot.RData")
# remove data from memory to allow for next set
rm(bmd2acc_rmsd_boot)
```

```
## Load in the Bootstrap Data
boot_files <- list.files(pattern = "boot.RData")
for(f in boot_files){load(f)}
## Obtain the Percentile Confidence Intervals for RMSD
rmsd_boot_potency_summary <- data.frame(
  RMSD_AC10 = c(ac10_rmsd_boot$t0,
                quantile(ac10_rmsd_boot$t,probs = c(0.025,0.975))),
  RMSD_AC50 = c(ac50_rmsd_boot$t0,
                quantile(ac50_rmsd_boot$t,probs = c(0.025,0.975))),
  RMSD_ACC = c(acc_rmsd_boot$t0,
               quantile(acc_rmsd_boot$t,probs = c(0.025,0.975))),
  RMSD_BMD2ACC_v4.0 = c(bmd2acc_rmsd_boot$t0,
                   quantile(bmd2acc_rmsd_boot$t,probs = c(0.025,0.975)))
)

round(rmsd_boot_potency_summary,digits = 3)
```

## 2.8 Benchmark Dose (BMD) Review

## BMD

### 2.8.1 Examine BMR and Cutoff

In addition to calculation of the AC50, AC10, ACC, and ACB potency
estimates mentioned above, benchmark dose (BMD) concentrations were
added.

```
#uses mc5 from invitrodb_v4_0_top_over_cutoff.Rdata
#data cleaning
threshold <- unique(mc5[, c('aeid','aenm','bmr', 'coff')])
threshold <- threshold[!coff==0]
threshold <- threshold[!bmr==0]
#log10 thresholds
threshold[,log10_bmr := log10(bmr)]
threshold[,log10_coff := log10(coff)]

#How often is bmr < coff?
threshold[, coff.bmr.diff := log10_coff - log10_bmr]
```

```
supp_fig5a <- ggplot(data=threshold) +
  geom_histogram(aes(x=coff.bmr.diff))+ 
  xlab('log10(Cutoff) - log10(BMR)')+
  ylab('Frequency of Difference')+
  scale_x_continuous(breaks=seq(-2,3,0.5))+
  theme_bw() +
  theme(axis.title.x = element_text(face='bold', size=14),
        axis.title.y = element_text(face='bold', size=14),
        axis.text.x = element_text(size=12),
        axis.text.y = element_text(size=12))
```

When we examine how often these values compare, BMR is less than
cutoff in 1161 aeids, and BMR greater than cutoff 315. Cutoff than BMD
is greater in 78.6585366% of endpoint-samples.

Other metrics can be output to evaluate the BMD vs COFF
difference:

```
Metric <- c("Mean", 
            "Median",
            "Standard Deviation")
Difference_in_log10_uM<- c(mean(threshold$coff.bmr.diff),
          median(threshold$coff.bmr.diff), 
          sd(threshold$coff.bmr.diff))

Table <- data.frame(Metric, Difference_in_log10_uM)

datatable(Table,
          filter='top',
          options=list(pageLength = 15,searching=FALSE, autoWidth=FALSE,  scrollX=TRUE, initComplete = JS(
            "function(settings, json) {",
            "$('body').css({'font-family': 'Calibri'});",
            "}"
          )))
```

Evaluating a linear regression model of BMR compared to COFF:

```
threshold.model <- lm(log10_bmr ~ log10_coff, data = threshold)
summary(threshold.model)
```

```
## 
## Call:
## lm(formula = log10_bmr ~ log10_coff, data = threshold)
## 
## Residuals:
##     Min      1Q  Median      3Q     Max 
## -1.7188 -0.2866 -0.0743  0.1709  1.6424 
## 
## Coefficients:
##             Estimate Std. Error t value Pr(>|t|)    
## (Intercept) -0.07418    0.01538  -4.823 1.56e-06 ***
## log10_coff   0.78998    0.01345  58.720  < 2e-16 ***
## ---
## Signif. codes:  0 '***' 0.001 '**' 0.01 '*' 0.05 '.' 0.1 ' ' 1
## 
## Residual standard error: 0.4761 on 1474 degrees of freedom
## Multiple R-squared:  0.7005, Adjusted R-squared:  0.7003 
## F-statistic:  3448 on 1 and 1474 DF,  p-value: < 2.2e-16
```

The RMSE value is 0.4757619 log10-uM.

```
supp_fig5b <- ggplot(data=threshold) +
  geom_point(aes(x=coff, y=bmr),size=2, alpha=0.2)+ 
  xlab('Log10 Cutoff')+
  ylab('Log10 BMR')+
  coord_cartesian(xlim=c(-3,3), ylim=c(-3,3))+
  # add reference lines
  geom_abline(slope = 1,intercept = 0,col = "gray")+
  geom_abline(slope = 1,intercept = c(-0.5,0.5),
              col = "purple",linetype = "dotted")+
  geom_abline(slope = 1,intercept = c(-2.5,2.5),
              col = "blue",linetype = "dashed")+
  theme_bw() +
  theme(axis.title.x = element_text(face='bold', size=14),
        axis.title.y = element_text(face='bold', size=14),
        axis.text.x = element_text(size=12),
        axis.text.y = element_text(size=12))+
  annotate('text', x=-2, y=2, face = 'bold', size = 5, label='Adj R2 = 0.7055')
```

```
supp_fig5 <- plot_grid(supp_fig5a, supp_fig5b, labels = c("A", "B"), label_size = 16, ncol=2, rel_widths = c(1,1),
          rel_heights = c(1,1))
supp_fig5
```

```
file.dir <- paste(getwd(), sep="")
file.name <- paste("/Supp_Fig_BMR_cutoff_", Sys.Date(), ".png", sep="")
file.path <- paste(file.dir, file.name, sep="")
dir.create(path=file.dir, showWarnings = FALSE, recursive = TRUE)
png(width=6000, height=4000, res=600)
supp_fig5
dev.off()
```

```
fig6b <- potency %>% 
  dplyr::filter(.,hitc_v4.0 >= 0.9) %>% 
  ggplot(.,aes(x = acc_v4.0,y = bmd_v4.0))+
  geom_point(alpha = 0.2,na.rm = TRUE)+
  # add reference lines
  geom_abline(slope = 1,intercept = 0,col = "gray")+
  geom_abline(slope = 1,intercept = c(-0.5,0.5),
              col = "purple",linetype = "dotted")+
  geom_abline(slope = 1,intercept = c(-2.5,2.5),
              col = "blue",linetype = "dashed")+
  # layout of the plots
  theme_bw() +
  theme(strip.background = element_blank(),
        strip.text.y = element_text(angle=360))+
  scale_x_continuous(breaks = seq(-10,10,2.5))+
  scale_y_continuous(breaks = seq(-10,10,2.5))+
  labs(x = "Log10 ACC (uM) in v4.0",
       y= "Log10 BMD (uM) in v4.0")+
  guides(col = guide_legend(title = "Flip Direction"))
# print the figure
fig6b
```

```
fig6_all <- ggdraw()+
  draw_plot(fig6a, 0, 0.5,1,0.5)+
  draw_plot(fig6b, 0,   0,1,0.5)+
  draw_plot_label(c('A','B'), c(0, 0), c(1, 0.5), size=16)
fig6_all
```

```
file.dir <- paste(getwd(), sep="")
file.name <- paste("/Fig6_potency_comp_", Sys.Date(), ".png", sep="")
file.path <- paste(file.dir, file.name, sep="")
dir.create(path=file.dir, showWarnings = FALSE, recursive = TRUE)
png(width=5000, height=5000, res=600)
fig6_all
dev.off()
```

```
potency %>% 
  dplyr::filter(.,hitc_v4.0 >= 0.9) %>% 
  ggplot(.,aes(x = acc_v4.0,y = bmd_v4.0,col = flip_dir))+
  geom_point(alpha = 0.2)+
  facet_grid(facets = "flip_dir")+
  scale_color_manual(values=c( "#009B95", "#4B0055"))+
  # add reference lines
  geom_abline(slope = 1,intercept = 0,col = "gray")+
  geom_abline(slope = 1,intercept = c(-0.5,0.5),
              col = "purple",linetype = "dotted")+
  geom_abline(slope = 1,intercept = c(-2.5,2.5),
              col = "blue",linetype = "dashed")+
  # layout of the plots
  theme_bw() +
  theme(strip.background = element_blank(),
        strip.text.y = element_text(angle=360))+
  scale_x_continuous(breaks = seq(-10,10,2.5))+
  scale_y_continuous(breaks = seq(-10,10,2.5))+
  labs(x = "Log10 ACC (uM) in v4.0",
       y= "Log10 BMD (uM) in v4.0")+
  guides(col = guide_legend(title = "Flip Direction"))
```

```
## Warning: Removed 12287 rows containing missing values (`geom_point()`).
```

```
potency %>% 
  dplyr::filter(.,flip_dir == "AA") %>% 
  ggplot(.,aes(x = acc_v4.0,y = bmd_v4.0,col = flip_dir))+
  geom_point(alpha = 0.2,na.rm = TRUE)+
  scale_color_manual(values=c( "#009B95", "#4B0055"))+
  # add reference lines
  geom_abline(slope = 1,intercept = 0,col = "gray")+
  geom_abline(slope = 1,intercept = c(-0.5,0.5),
              col = "purple",linetype = "dotted")+
  geom_abline(slope = 1,intercept = c(-2.5,2.5),
              col = "blue",linetype = "dashed")+
  # layout of the plots
  theme_bw() +
  theme(strip.background = element_blank(),
        strip.text.y = element_text(angle=360))+
  scale_x_continuous(breaks = seq(-10,10,2.5))+
  scale_y_continuous(breaks = seq(-10,10,2.5))+
  labs(x = "Log10 ACC (uM) in v4.0",
       y= "Log10 BMD (uM) in v4.0")+
  guides(col = guide_legend(title = "Flip Direction"))
```

### 2.8.2 BMDU to BMDL Interval

```
mc5[, log10_bmdu := log10(bmdu)]
mc5[, log10_bmdl := log10(bmdl)]
mc5[, log10_ci_width := log10_bmdu - log10_bmdl, by=m4id]
```

38.9008276% of endpoint samples do not have a BMD lower bound (BMDL).
For the series with a full BMDL and BMD lower bound (BMDU) interval
calculated:

```
Metric <- c("Mean", 
            "Median",
            "Standard Deviation")
BMDL_BMDU_CI_width_in_log10_uM<- c( mean(mc5$log10_ci_width, na.rm=TRUE),
          median(mc5$log10_ci_width, na.rm=TRUE), 
          sd(mc5$log10_ci_width, na.rm = TRUE))

Table <- data.frame(Metric, BMDL_BMDU_CI_width_in_log10_uM)

datatable(Table,
          filter='top',
          options=list(pageLength = 15,searching=FALSE, autoWidth=FALSE,  scrollX=TRUE, initComplete = JS(
            "function(settings, json) {",
            "$('body').css({'font-family': 'Calibri'});",
            "}"
          )))
```

We can examine how ACC compares to BMDL-BMDU interval?

```
mc5[, acc_lt_bmdu := acc < bmdu]
mc5[, acc_gt_bmdl := ifelse(!is.na(bmdl), acc > bmdl, acc>bmd)]
mc5[, acc_in_ci := acc_lt_bmdu==TRUE & acc_gt_bmdl==TRUE]


Output <- c("Count", 
            "Total",
            "Percent")
ACC_less_than_BMDU<- c( nrow(mc5[acc_lt_bmdu==TRUE]),
          nrow(mc5[!is.na(bmdu)]), 
          ((nrow(mc5[acc_lt_bmdu==TRUE])/ (nrow(mc5[!is.na(bmdu)]))*100 )))

ACC_greater_than_BMDL<- c( nrow(mc5[acc_gt_bmdl==TRUE]),
          nrow(mc5[!is.na(bmdl)]), 
          ((nrow(mc5[acc_gt_bmdl==TRUE])/ (nrow(mc5[!is.na(bmdl)]))*100 )))

ACC_in_CI<- c( nrow(mc5[acc_in_ci==TRUE]),
          nrow(mc5[!is.na(acc_lt_bmdu) & !is.na(acc_gt_bmdl)]), 
          ((nrow(mc5[acc_in_ci==TRUE])/ (nrow(mc5[!is.na(acc_lt_bmdu) & !is.na(acc_gt_bmdl)]))*100 )))

Table <- data.frame(Output, ACC_less_than_BMDU, ACC_greater_than_BMDL, ACC_in_CI)

datatable(Table,
          filter='top',
          options=list(pageLength = 15,searching=FALSE, autoWidth=FALSE,  scrollX=TRUE, initComplete = JS(
            "function(settings, json) {",
            "$('body').css({'font-family': 'Calibri'});",
            "}"
          )))
```

```
ggplot(data=mc5[acc_in_ci==TRUE]) +
  geom_point(aes(x=acc, y=bmd),size=2, alpha=0.2)+ 
  geom_segment(aes(y=bmdl, yend=bmdu, x=acc, xend=acc))+
  xlab('Cutoff')+
  ylab('BMR')+
  coord_cartesian(xlim=c(10^-3,10^3), ylim=c(10^-3,10^3))+
  scale_x_continuous(trans=log10_trans(),
                     breaks=trans_breaks("log10", function(x) 10^x),
                     labels=trans_format("log10", math_format(10^.x)))+
    scale_y_continuous(trans=log10_trans(),
                     breaks=trans_breaks("log10", function(x) 10^x),
                     labels=trans_format("log10", math_format(10^.x)))+
  theme_bw()+
  theme(axis.title.x = element_text(face='bold', size=14),
        axis.title.y = element_text(face='bold', size=14),
        axis.text.x = element_text(size=12),
        axis.text.y = element_text(size=12))
```

# 3 Assay Source Comparison

Data may be stratified by assay source to further examine differences
between versions.

## 3.1 Flipped Hit Calls

## Flipped Hit Calls

This flip direction analysis was extended to examine flipped hit
calls across the 25 assay sources between versions. Majority of assay
sources show negligible change, with only four assay sources showing
greater than 10% change.

```
#-----------------------------------------------------------------------------------#
# FLIPPED HITS BY ASSAY SOURCE
#-----------------------------------------------------------------------------------#
as_flip <- potency %>% 
  filter(!aenm_v4.0=="DELETED") %>% #filter to remove duplicate counts
  group_by(asnm, flip_dir)  %>%
  count() %>% 
  group_by(asnm) %>%
  mutate(perc = `n` / sum(`n`))
```

```
ggplot(as_flip, aes(x = "", y = perc, fill = flip_dir)) +
  geom_bar(stat="identity", color="black") + facet_grid(rows=vars(as_flip$asnm)) +
  #scale_fill_manual(values=c("#C87A8A",  "#9189C7", "#2A9EB5", "#A29048")) +
  scale_fill_manual(values=c('#2A788EFF', '#FDE725FF', '#414487FF','#7AD151FF'))+
  guides(fill = guide_legend(title = "Flip Direction")) + coord_flip() + theme_void()
```

```
fig4c <- ggplot(as_flip, aes(x = "", y = perc, fill = flip_dir)) +
  geom_bar(stat="identity", color="black") + facet_grid(rows=vars(as_flip$asnm)) +
  #scale_fill_manual(values=c("#C87A8A",  "#9189C7", "#2A9EB5", "#A29048")) +
  scale_fill_manual(values=c('#2A788EFF', '#FDE725FF', '#414487FF','#7AD151FF'))+
  coord_flip() + theme_void()+
  theme(legend.position='none',
        axis.text = element_text(size=12),
        text = element_text(size=12))
```

```
fig4_all <- ggdraw()+
  draw_plot(fig4a, 0,0.5,0.5,0.5)+
  draw_plot(fig4b, 0.5, 0.5,0.5, 0.5 )+
  draw_plot(fig4c, 0, 0,1, 0.5 )+
  draw_plot_label(c('A','B','C'), c(0, 0.5, 0), c(1, 1, 0.5), size=16)
```

```
file.dir <- paste(getwd(), sep="")
file.name <- paste("/Fig4_hitc_flip_", Sys.Date(), ".png", sep="")
file.path <- paste(file.dir, file.name, sep="")
dir.create(path=file.dir, showWarnings = FALSE, recursive = TRUE)
png(width=8000, height=6000, res=600)
fig4_all
dev.off()
```

Assay design specifics within each assay source could also provide
insight into observed flipping. For example, CEETOX and BSK have low
number of data points screened (e.g. 4 concentrations tested with 2
replicates) and high proportion of inactive to active flipping occurring
between versions.

Relative proportions could also be skewed if the assay source
contains only a few endpoints or screened a small number of chemicals.
This table reflects endpoint-sample counts per assay source and relative
change in percent of active hit calls changed in v3.5 to v4.0, which
often corresponds to a reduction in inactive hit calls given deleted
endpoints.

## 3.2 Winning Model Selection

## Winning Model Selection

Winning model selection for each assay source between versions can be
visualized. In this figure, each block shows relative model selection
within each assay source. This does not distinguish between positive and
negative hit calls. In v3.5, a hit call of -1 correspond to a “NA” model
fitting. These are concentration-response series that tcpl was unable to
fit, usually due to limited number of concentrations tested, but are
represented as “inactive” in visuals below. In v4.0, these series may
appear fit in model “none” with a hit call of 0, therefore inactive. In
the new tcpl paradigm, constant model can never win.

Models included: “hill”, “gnls”(gain-loss), “cnst”(constant),
“pow”(power), “poly1”(polynomial-1: linear), “poly2”(polynomial-2:
quadratic), “exp2”(exponential-2), “exp3”(exponential-3),
“exp4”(exponential-4), “exp5”(exponential-5), NA (unable to fit in
v3.5), and “none”(unable to fit in v4.0).

```
as_modl_v3.5 <- potency %>% 
  group_by(asnm, modl_v3.5)  %>%
  filter(!is.na(asnm)) %>%
  count() %>%
  group_by(asnm) %>%
  mutate(perc = `n` / sum(`n`))
setnames(as_modl_v3.5, "modl_v3.5", "modl")
as_modl_v3.5$modl <- factor(as_modl_v3.5$modl, levels = c("hill", "gnls", "cnst"))

p_as_modl_v3.5 <- ggplot(as_modl_v3.5, aes(x = "", y = perc, fill = modl)) +
  geom_bar(stat="identity", color="black") + facet_grid(rows=vars(as_modl_v3.5$asnm)) +
  scale_fill_manual(values=c("#FDE333", "#B2DC3C", "#AF8B52" )) +
  guides(fill = guide_legend(title = "Model")) + coord_flip() + theme_void()

as_modl_v4.0 <- potency %>%
  filter(!aenm_v4.0=="DELETED") %>% #filter to remove duplicate counts
  group_by(asnm, modl_v4.0)  %>%
  count() %>%
  group_by(asnm) %>%
  mutate(perc = `n` / sum(`n`))
setnames(as_modl_v4.0, "modl_v4.0", "modl")
as_modl_v4.0$modl <- factor(as_modl_v4.0$modl, levels = c("hill", "gnls", "pow", "poly1", "poly2", "exp2", "exp3", "exp4", "exp5", "none"))

p_as_modl_v4.0 <- ggplot(as_modl_v4.0, aes(x = "", y = perc, fill = modl)) +
  geom_bar(stat="identity", color="black") + facet_grid(rows=vars(as_modl_v4.0$asnm)) +
  scale_fill_manual(values=c("#FDE333", "#B2DC3C", "#53CC67", "#00B785", "#009B95" ,"#007B97", "#00588B", "#185086" , "#422C70", "#4B0055")) +
  guides(fill = guide_legend(title = "Model")) + coord_flip() + theme_void()

p_as_modl_v3.5 + p_as_modl_v4.0
```

## 3.3 Winning Model Selection, Active Hits Only

## Winning Model Selection, Active Hits Only

This data was filtered to remove endpoint-samples that were inactive
between both versions. In this figure, each block shows relative model
selection within each assay source. With the inactive-constant hits
removed, hill and gain-loss were only options in v3.5. In v4.0, new
tcplFit2 models were selected for active fits in addition to hill and
gain-Loss. Models included: “hill”, “gnls”(Gain-Loss), “pow”(power),
“poly1”(polynomial-1: linear), “poly2”(polynomial-2: quadratic),
“exp2”(exponential-2), “exp3”(exponential-3), “exp4”(exponential-4),
“exp5”(exponential-5).

Trends in winning model selection are minor and/or not apparent.

```
as_active_modl_v3.5 <- potency %>% 
  filter(act_v3.5=="active")  %>%
  group_by(asnm, modl_v3.5)  %>%
  filter(!is.na(asnm)) %>%
  count() %>%
  group_by(asnm) %>%
  mutate(perc = `n` / sum(`n`))
setnames(as_active_modl_v3.5, "modl_v3.5", "modl")
as_active_modl_v3.5$modl <- factor(as_active_modl_v3.5$modl, levels = c("hill", "gnls", "cnst"))

p_as_active_modl_v3.5 <- ggplot(as_active_modl_v3.5, aes(x = "", y = perc, fill = modl)) +
  geom_bar(stat="identity", color="black") + facet_grid(rows=vars(as_active_modl_v3.5$asnm)) +
  scale_fill_manual(values=c("#FDE333", "#B2DC3C")) +
  guides(fill = guide_legend(title = "Winning Model")) + coord_flip() + theme_void()

as_active_modl_v4.0 <- potency %>%
  filter(act_v4.0=="active")  %>%
  filter(!aenm_v4.0=="DELETED") %>% #filter to remove duplicate counts
  group_by(asnm, modl_v4.0)  %>%
  count() %>%
  group_by(asnm) %>%
  mutate(perc = `n` / sum(`n`))
setnames(as_active_modl_v4.0, "modl_v4.0", "modl")
as_active_modl_v4.0$modl <- factor(as_active_modl_v4.0$modl, levels = c("hill", "gnls", "pow", "poly1", "poly2", "exp2", "exp3", "exp4", "exp5"))

p_as_active_modl_v4.0 <- ggplot(as_active_modl_v4.0, aes(x = "", y = perc, fill = modl)) +
  geom_bar(stat="identity", color="black") + facet_grid(rows=vars(as_active_modl_v4.0$asnm)) +
  scale_fill_manual(values=c("#FDE333", "#B2DC3C", "#53CC67", "#00B785", "#009B95" ,"#007B97", "#00588B", "#185086" , "#422C70", "#4B0055")) +
  guides(fill = guide_legend(title = "Winning Model")) + coord_flip() + theme_void()

p_as_active_modl_v3.5 + p_as_active_modl_v4.0
```

## 3.4 Log10 Potency

## Log10 Potency

Point of departure potency estimates were based on modeled active
concentration series, including ACC (activity concentration at cutoff),
AC10 (activity concentration at 10% of maximal response), and AC50
(activity concentration at 50% of maximal response). Based on the
program BMDExpress, tcplFit2 modeling outputs new potency and
uncertainty estimates related to a benchmark dose (BMD) as defined by
the Benchmark Response (BMR) level. BMR is estimated as one standard
deviation of the baseline response for the endpoint using the two lowest
concentrations across all tested chemicals. Potency analysis examines
Log10 potency values, or the logged tested concentration in uM.

### 3.4.1 Log10 Potency Distributions

To examine the log10 potency distributions, data was first filtered
to remove endpoint-samples that were inactive between both versions.
Inactives in v3.5 (hitc=0) do not have any associated potency values
(NA) therefore they cannot be compared to v4.0 values. In this series of
figures, each row corresponds to potency estimates by version for each
assay source.

There appears to be little change in potency values for most assay
sources between versions.

```
#subset remove rows where always inactive between versions since these often do not have potency values
potency_as_comp_long <- as.data.table(melt(potency_active,
                                  # ID variables - all the variables to keep but not split apart on
                                  id.vars=c("spid" ,"asnm",  "acid" , "acnm_v4.0" , "aeid",  "aenm_v3.5", "aenm_v4.0",  "chnm"),
                                  # The source columns
                                  measure.vars=c( "bmd_v4.0","ac50_v3.5", "ac50_v4.0", "acc_v3.5", "acc_v4.0", "ac10_v3.5", "ac10_v4.0" ),
                                  # Name of the destination column that will identify the original
                                  # column that the measurement came from
                                  variable.name="potency",
                                  value.name="value"))
#reformat
potency_as_comp_long$version <- "v4.0"
potency_as_comp_long[potency %in% c("ac50_v3.5","ac10_v3.5","acc_v3.5"), version:= "v3.5"]
potency_as_comp_long[potency %in% c("ac50_v3.5","ac50_v4.0"), potency:= "ac50"]
potency_as_comp_long[potency %in% c("ac10_v3.5","ac10_v4.0"), potency:= "ac10"]
potency_as_comp_long[potency %in% c("acc_v3.5","acc_v4.0"), potency:= "acc"]
potency_as_comp_long[potency %in% c("bmd_v4.0"), potency:= "bmd"]

#filter to remove instances if NA in either one version (limit to values used in stat test comparison)
potency_as_comp_long <- potency_as_comp_long %>% 
  group_by(spid, aeid, potency) %>% 
    filter(!any(is.na(value)))

potency_as_comp_long <- as.data.table(potency_as_comp_long)
potency_as_comp_long_ac50 <- potency_as_comp_long[potency =="ac50",]
potency_as_comp_long_ac10 <- potency_as_comp_long[potency =="ac10",]
potency_as_comp_long_acc <- potency_as_comp_long[potency =="acc",]
potency_as_comp_long_bmd <- potency_as_comp_long[potency =="bmd",]
potency_as_comp_long_acc_bmd <- potency_as_comp_long[potency %in% c("acc", "bmd"),]

ggplot(data = potency_as_comp_long_ac50, aes(x = value, y = potency, fill=version, na.rm=TRUE)) +
  geom_boxplot(outlier.shape = NA) +
  facet_grid(rows=vars(asnm)) + theme_minimal() +
  theme(strip.background = element_blank(),
        strip.text.y = element_text(angle=360),
        axis.text.y = element_blank(),
        axis.ticks.y = element_blank(),
        axis.line.y = element_blank()) +
  scale_fill_manual(values=c("#4B0055","#53CC67")) +
  xlim(-5, 5) +
  labs(y= "AC50 by Assay Source", x = "Log10 Potency (uM)")
```

```
ggplot(data = potency_as_comp_long_acc, aes(x = value, y = potency, fill=version, na.rm=TRUE)) +
  geom_boxplot(outlier.shape = NA) +
  facet_grid(rows=vars(asnm)) + theme_minimal() +
  theme(strip.background = element_blank(),
        strip.text.y = element_text(angle=360),
        axis.text.y = element_blank(),
        axis.ticks.y = element_blank(),
        axis.line.y = element_blank()) +
  scale_fill_manual(values=c("#4B0055","#53CC67")) +
  xlim(-5, 5) +
  labs(y= "ACC by Assay Source", x = "Log10 Potency (uM)") +
  guides(fill = guide_legend(title = "Version"))
```

```
ggplot(data = potency_as_comp_long_acc_bmd, aes(x = value, y = potency, fill=version, color= potency, na.rm=TRUE)) +
  geom_boxplot(outlier.shape = NA) +
  facet_grid(rows=vars(asnm)) + theme_minimal() +
  theme(strip.background = element_blank(),
        strip.text.y = element_text(angle=360),
        axis.text.y = element_blank(),
        axis.ticks.y = element_blank(),
        axis.line.y = element_blank()) +
  scale_fill_manual(values=c("gray", "white")) +
  scale_color_manual(values=c("#4B0055","#53CC67")) +
  xlim(-5, 5) +
  labs(y= "ACC + BMD by Assay Source", x = "Log10 Potency (uM)") +
  guides(fill = guide_legend(title = "Version", color= guide_legend(title = "Potency")))
```

```
ggplot(data = potency_as_comp_long_ac10, aes(x = value, y = potency, fill=version, na.rm=TRUE)) +
  geom_boxplot(outlier.shape = NA) +
  facet_grid(rows=vars(asnm)) + theme_minimal() +
  theme(strip.background = element_blank(),
        strip.text.y = element_text(angle=360),
        axis.text.y = element_blank(),
        axis.ticks.y = element_blank(),
        axis.line.y = element_blank()) +
  scale_fill_manual(values=c("#4B0055","#53CC67"))  +
  xlim(-5, 5) +
  labs(y= "AC10 by Assay Source", x = "Log10 Potency (uM)") +
  guides(fill = guide_legend(title = "Version"))
```

```
ggplot(data = potency_as_comp_long_bmd, aes(x = value, y = potency, fill=version, na.rm=TRUE)) +
  geom_boxplot(outlier.shape = NA) +
  facet_grid(rows=vars(asnm)) + theme_minimal() +
  theme(strip.background = element_blank(),
        strip.text.y = element_text(angle=360),
        axis.text.y = element_blank(),
        axis.ticks.y = element_blank(),
        axis.line.y = element_blank()) +
  scale_fill_manual(values=c("white")) +
  xlim(-5, 5) +
  labs(y= "BMD by Assay Source", x = "Log10 Potency (uM)") +
  guides(fill = guide_legend(title = "Version"))
```

### 3.4.2 Magnitude of Log10 Potency Change

Magnitude of potency change was assessed by calculating the log 10
absolute difference in point of departure potency estimates between v3.5
and v4.0, where potency value reflects the unlogged tested concentration
in uM.

Majority fall around .5log10 change for these potency estimates
across the database between versions, although there are outliers.

```
#subset remove rows where always inactive between versions since these often do not have potency values
potency_as_change_long <- as.data.table(melt(potency_active,
                                  # ID variables - all the variables to keep but not split apart on
                                  id.vars=c("spid" , "asnm", "acid" , "acnm_v4.0" , "aeid",  "aenm_v3.5", "aenm_v4.0",  "chnm"),
                                  # The source columns
                                  measure.vars=c("ac50_change", "acc_change", "ac10_change"),
                                  # Name of the destination column that will identify the original
                                  # column that the measurement came from
                                  variable.name="change",
                                  value.name="value"))

potency_as_change_long_ac50 <- potency_as_change_long[change =="ac50_change",]
potency_as_change_long_ac10 <- potency_as_change_long[change =="ac10_change",]
potency_as_change_long_acc <- potency_as_change_long[change =="acc_change",]

ggplot(data = potency_as_change_long_ac50, aes(x = value, y = change, na.rm=TRUE)) +
  geom_boxplot(outlier.shape = NA) +
  facet_grid(rows=vars(asnm)) + theme_minimal() +
  theme(strip.background = element_blank(),
        strip.text.y = element_text(angle=360),
        axis.text.y = element_blank(),
        axis.ticks.y = element_blank(),
        axis.line.y = element_blank()) +
  xlim(-5, 5)  +
  labs(y= "AC50 by Assay Source", x = "Log10 Potency Change (uM)")
```

```
ggplot(data = potency_as_change_long_acc, aes(x = value, y = change, na.rm=TRUE)) +
  geom_boxplot(outlier.shape = NA) +
  facet_grid(rows=vars(asnm)) + theme_minimal() +
  theme(strip.background = element_blank(),
        strip.text.y = element_text(angle=360),
        axis.text.y = element_blank(),
        axis.ticks.y = element_blank(),
        axis.line.y = element_blank()) +
  xlim(-5, 5) +
  labs(y= "ACC by Assay Source", x = "Log10 Potency Change (uM)")
```

```
ggplot(data = potency_as_change_long_ac10, aes(x = value, y = change, na.rm=TRUE)) +
  geom_boxplot(outlier.shape = NA) +
  facet_grid(rows=vars(asnm)) + theme_minimal() +
  theme(strip.background = element_blank(),
        strip.text.y = element_text(angle=360),
        axis.text.y = element_blank(),
        axis.ticks.y = element_blank(),
        axis.line.y = element_blank()) +
  xlim(-5, 5) +
  labs(y= "AC10 by Assay Source", x = "Log10 Potency Change (uM)")
```

## 3.5 Log10 Potency - AA only

## Log10 Potency - AA only

Potency analysis can be repeated to examine Log10 potency values, or
the logged tested concentration in uM.

### 3.5.1 Log10 Potency Distributions

To examine the log10 potency distributions, data was first filtered
to remove endpoint-samples that were not active in both versions. In
this series of figures, each row corresponds to potency estimates by
version for each assay source.

There appears to be little change in potency values for most assay
sources between versions. In comparing these distributions back to those
which consider both AA and AI cases, AC50 medians for TAMU and VALA
became less aligned, ACC median alignment worsened for BSK and VALA and
improved for TAMU and UPITT, and AC10 median alignment improved for TAMU
and worsened slightly for LTEA, UPITT, and CCTE SHAFER.

```
#subset remove rows where always inactive between versions since these often do not have potency values
potency_as_comp_long <- as.data.table(melt(potency_active_AA,
                                  # ID variables - all the variables to keep but not split apart on
                                  id.vars=c("spid" ,"asnm",  "acid" , "acnm_v4.0" , "aeid",  "aenm_v3.5", "aenm_v4.0",  "chnm"),
                                  # The source columns
                                  measure.vars=c( "bmd_v4.0","ac50_v3.5", "ac50_v4.0", "acc_v3.5", "acc_v4.0", "ac10_v3.5", "ac10_v4.0" ),
                                  # Name of the destination column that will identify the original
                                  # column that the measurement came from
                                  variable.name="potency",
                                  value.name="value"))
#reformat
potency_as_comp_long$version <- "v4.0"
potency_as_comp_long[potency %in% c("ac50_v3.5","ac10_v3.5","acc_v3.5"), version:= "v3.5"]
potency_as_comp_long[potency %in% c("ac50_v3.5","ac50_v4.0"), potency:= "ac50"]
potency_as_comp_long[potency %in% c("ac10_v3.5","ac10_v4.0"), potency:= "ac10"]
potency_as_comp_long[potency %in% c("acc_v3.5","acc_v4.0"), potency:= "acc"]
potency_as_comp_long[potency %in% c("bmd_v4.0"), potency:= "bmd"]

#filter to remove instances if NA in either one version (limit to values used in stat test comparison)
potency_as_comp_long <- potency_as_comp_long %>% 
  group_by(spid, aeid, potency) %>% 
    filter(!any(is.na(value)))

potency_as_comp_long <- as.data.table(potency_as_comp_long)
potency_as_comp_long_ac50 <- potency_as_comp_long[potency =="ac50",]
potency_as_comp_long_ac10 <- potency_as_comp_long[potency =="ac10",]
potency_as_comp_long_acc <- potency_as_comp_long[potency =="acc",]
potency_as_comp_long_bmd <- potency_as_comp_long[potency =="bmd",]
potency_as_comp_long_acc_bmd <- potency_as_comp_long[potency %in% c("acc", "bmd"),]

ggplot(data = potency_as_comp_long_ac50, aes(x = value, y = potency, fill=version, na.rm=TRUE)) +
  geom_boxplot(outlier.shape = NA) +
  facet_grid(rows=vars(asnm)) + theme_minimal() +
  theme(strip.background = element_blank(),
        strip.text.y = element_text(angle=360),
        axis.text.y = element_blank(),
        axis.ticks.y = element_blank(),
        axis.line.y = element_blank()) +
  scale_fill_manual(values=c("#4B0055","#53CC67"))  +
  xlim(-5, 5) +
  labs(y= "AC50 by Assay Source", x = "Log10 Potency (uM)")
```

```
ggplot(data = potency_as_comp_long_acc, aes(x = value, y = potency, fill=version, na.rm=TRUE)) +
  geom_boxplot(outlier.shape = NA) +
  facet_grid(rows=vars(asnm)) + theme_minimal() +
  theme(strip.background = element_blank(),
        strip.text.y = element_text(angle=360),
        axis.text.y = element_blank(),
        axis.ticks.y = element_blank(),
        axis.line.y = element_blank()) +
  scale_fill_manual(values=c("#4B0055","#53CC67")) +
  xlim(-5, 5) +
  labs(y= "ACC by Assay Source", x = "Log10 Potency (uM)") +
  guides(fill = guide_legend(title = "Version"))
```

```
ggplot(data = potency_as_comp_long_acc_bmd, aes(x = value, y = potency, fill=version, color= potency, na.rm=TRUE)) +
  geom_boxplot(outlier.shape = NA) +
  facet_grid(rows=vars(asnm)) + theme_minimal() +
  theme(strip.background = element_blank(),
        strip.text.y = element_text(angle=360),
        axis.text.y = element_blank(),
        axis.ticks.y = element_blank(),
        axis.line.y = element_blank()) +
  scale_fill_manual(values=c("gray", "white")) +
  scale_color_manual(values=c("#4B0055","#53CC67")) +
  xlim(-5, 5) +
  labs(y= "ACC + BMD by Assay Source", x = "Log10 Potency (uM)") +
  guides(fill = guide_legend(title = "Version", color= guide_legend(title = "Potency")))
```

```
ggplot(data = potency_as_comp_long_ac10, aes(x = value, y = potency, fill=version, na.rm=TRUE)) +
  geom_boxplot(outlier.shape = NA) +
  facet_grid(rows=vars(asnm)) + theme_minimal() +
  theme(strip.background = element_blank(),
        strip.text.y = element_text(angle=360),
        axis.text.y = element_blank(),
        axis.ticks.y = element_blank(),
        axis.line.y = element_blank()) +
  scale_fill_manual(values=c("#4B0055","#53CC67")) +
  xlim(-5, 5) +
  labs(y= "AC10 by Assay Source", x = "Log10 Potency (uM)") +
  guides(fill = guide_legend(title = "Version"))
```

```
ggplot(data = potency_as_comp_long_bmd, aes(x = value, y = potency, fill=version, na.rm=TRUE)) +
  geom_boxplot(outlier.shape = NA) +
  facet_grid(rows=vars(asnm)) + theme_minimal() +
  theme(strip.background = element_blank(),
        strip.text.y = element_text(angle=360),
        axis.text.y = element_blank(),
        axis.ticks.y = element_blank(),
        axis.line.y = element_blank()) +
    scale_fill_manual(values=c("white")) +
  xlim(-5, 5) +
  labs(y= "BMD by Assay Source", x = "Log10 Potency (uM)") +
  guides(fill = guide_legend(title = "Version"))
```

### 3.5.2 Log10 Difference in Medians of Raw ACC and of Raw AC50 Between Versions

```
potency_as_raw_comp_long <- as.data.table(melt(potency_active,
                                  # ID variables - all the variables to keep but not split apart on
                                  id.vars=c("spid" ,"asnm", "acid" , "acnm_v4.0" , "aeid",  "aenm_v3.5", "aenm_v4.0",  "chnm"),
                                  # The source columns
                                  measure.vars=c( "bmd_raw_v4.0","ac50_raw_v3.5", "ac50_raw_v4.0", "acc_raw_v3.5", "acc_raw_v4.0", "ac10_raw_v3.5", "ac10_raw_v4.0"),
                                  # Name of the destination column that will identify the original
                                  # column that the measurement came from
                                  variable.name="potency",
                                  value.name="value"))
#reformat
potency_as_raw_comp_long$version <- "v4.0"
potency_as_raw_comp_long[potency %in% c("ac50_raw_v3.5","ac10_raw_v3.5","acc_raw_v3.5"), version:= "v3.5"]
potency_as_raw_comp_long[potency %in% c("ac50_raw_v3.5","ac50_raw_v4.0"), potency:= "ac50"]
potency_as_raw_comp_long[potency %in% c("ac10_raw_v3.5","ac10_raw_v4.0"), potency:= "ac10"]
potency_as_raw_comp_long[potency %in% c("acc_raw_v3.5","acc_raw_v4.0"), potency:= "acc"]
potency_as_raw_comp_long[potency %in% c("bmd_raw_v4.0"), potency:= "bmd"]

#filter to remove instances if NA in either one version (limit to values used in stat test comparison)
potency_as_raw_comp_long <- potency_as_raw_comp_long %>% 
  group_by(spid, aeid, potency) %>% 
    filter(!any(is.na(value)))

potency_as_raw_comp_long <- as.data.table(potency_as_raw_comp_long)
potency_as_raw_comp_long_ac50 <- potency_as_raw_comp_long[potency =="ac50",]
potency_as_raw_comp_long_ac10 <- potency_as_raw_comp_long[potency =="ac10",]
potency_as_raw_comp_long_acc <- potency_as_raw_comp_long[potency =="acc",]
potency_as_raw_comp_long_bmd <- potency_as_raw_comp_long[potency =="bmd",]
potency_as_raw_comp_long_acc_bmd <- potency_as_raw_comp_long[potency %in% c("acc", "bmd"),]


 med.diffs.acc <- potency_as_raw_comp_long_acc %>%
  group_by(asnm, version) %>%
  summarize(median = median(value)) %>% #median of raw acc values
  summarize(log10_diff_in_acc_median = signif(log10(abs(diff(median))),4)) %>%
  arrange(desc(log10_diff_in_acc_median))

med.diffs.ac50 <- potency_as_raw_comp_long_ac50 %>%
  group_by(asnm, version) %>%
  summarize(median = median(value)) %>% #median of raw ac50 values
  summarize(log10_diff_in_ac50_median = signif(log10(abs(diff(median))),4)) %>%
  arrange(desc(log10_diff_in_ac50_median))

log10_median_diffs_AA <- cbind(med.diffs.acc, med.diffs.ac50)
```

```
#make results table
datatable(log10_median_diffs_AA,
          caption = "Log10 Absolute Difference in ACC Median and AC50 Median Between v3.5 and v4.0, by Assay Source",
          filter='top',
          options=list(pageLength = 10,searching=FALSE, autoWidth=FALSE,  scrollX=TRUE, initComplete = JS(
            "function(settings, json) {",
            "$('body').css({'font-family': 'Calibri'});",
            "}"
          )))
```

### 3.5.3 Magnitude of Log10 Potency Change

Magnitude of potency change was assessed by calculating the log 10
absolute difference in point of departure potency estimates between v3.5
and v4.0, where potency value reflects the unlogged tested concentration
in uM.

Majority fall around .5log10 change for these potency estimates
across the database between versions, although there are outliers.

```
#subset remove rows where always inactive between versions since these often do not have potency values
potency_as_change_long <- as.data.table(melt(potency_active_AA,
                                  # ID variables - all the variables to keep but not split apart on
                                  id.vars=c("spid" , "asnm", "acid" , "acnm_v4.0" , "aeid",  "aenm_v3.5", "aenm_v4.0",  "chnm"),
                                  # The source columns
                                  measure.vars=c("ac50_change", "acc_change", "ac10_change"),
                                  # Name of the destination column that will identify the original
                                  # column that the measurement came from
                                  variable.name="change",
                                  value.name="value"))

potency_as_change_long_ac50 <- potency_as_change_long[change =="ac50_change",]
potency_as_change_long_ac10 <- potency_as_change_long[change =="ac10_change",]
potency_as_change_long_acc <- potency_as_change_long[change =="acc_change",]

ggplot(data = potency_as_change_long_ac50, aes(x = value, y = change, na.rm=TRUE)) +
  geom_boxplot(outlier.shape = NA) +
  facet_grid(rows=vars(asnm)) + theme_minimal() +
  theme(strip.background = element_blank(),
        strip.text.y = element_text(angle=360),
        axis.text.y = element_blank(),
        axis.ticks.y = element_blank(),
        axis.line.y = element_blank()) +
  xlim(-5, 5)  +
  labs(y= "AC50 by Assay Source", x = "Log10 Potency Change (uM)")
```

```
ggplot(data = potency_as_change_long_acc, aes(x = value, y = change, na.rm=TRUE)) +
  geom_boxplot(outlier.shape = NA) +
  facet_grid(rows=vars(asnm)) + theme_minimal() +
  theme(strip.background = element_blank(),
        strip.text.y = element_text(angle=360),
        axis.text.y = element_blank(),
        axis.ticks.y = element_blank(),
        axis.line.y = element_blank()) +
  xlim(-5, 5) +
  labs(y= "ACC by Assay Source", x = "Log10 Potency Change (uM)")
```

```
ggplot(data = potency_as_change_long_ac10, aes(x = value, y = change, na.rm=TRUE)) +
  geom_boxplot(outlier.shape = NA) +
  facet_grid(rows=vars(asnm)) + theme_minimal() +
  theme(strip.background = element_blank(),
        strip.text.y = element_text(angle=360),
        axis.text.y = element_blank(),
        axis.ticks.y = element_blank(),
        axis.line.y = element_blank()) +
  xlim(-5, 5) +
  labs(y= "AC10 by Assay Source", x = "Log10 Potency Change (uM)")
```

# 4 Cytotoxicity Threshold Change

Estimates of chemical concentrations that elicit cytotoxicity and/or
cell stress have been informative for contextualizing bioactivity
screening data in ToxCast and the likelihood that these data may be
confounded by assay interference resulting from cytotoxicity. General
estimates of the median and lower bound concentrations that might elicit
cytotoxicity and/or cell stress in vitro across a suite of cell-based
assays of different kinds have previously been calculated using the tcpl
function, tcplCytoPt, which considers activity across a suite of
cell-based assays. The resultant cytotoxicity threshold change from this
update to the tcplCytoPt() function was evaluated via comparison of
burst endpoint data in invitrodb v3.5 and v4.0 (noting that the cytotox
table in invitrodb v4.0 has not been updated).

## 4.1 Examine endpoint change between versions

v3.5 has 91 burst endpoints, whereas v4.0 currently has 73 given
deleted BSK and APR endpoints. The following table lists endpoints
included in each version:

## 4.2 Compare cytotoxicity burst calculation between versions

The tcplCytoPt function has been updated in response to major changes
in invitrodb and curve-fitting, namely via changes in units and a
requirement that a chemical included in a computation of the global
median absolute deviation (MAD), an estimate of the variance expected
for a chemical tested in many cytotoxicity and cell stress assays, be
assayed in greater than or equal to 60 assay endpoints annotated as
“burst” endpoints (previously it was required that the chemicals
included in the global MAD calculation be assayed in all cytotoxicity
burst assay endpoints). Additional filtering of burst assay data was
also required to ensure only losses in cell viability were included and
any cell proliferation responses were excluded given the bidirectional
endpoints.

```
#incorporate code from from https://github.com/USEPA/CompTox-ToxCast-tcpl/blob/21-tcplcytopt-update/R/tcplCytoPt.R
  #cat("7: Filtering chemical data (if necessary)\n")
  zdat <- v3.5_mc5[!is.na(chid),]
  #filter out gnls curves
  zdat <- zdat[modl != "gnls",]

  #cat("8: Determining representative sample\n") 
  #tcplSubsetChid doesn't seem to be backwards compatible, so use the mc5_chid to pull representative information
  con <- dbConnect(drv = RMySQL::MySQL(), user="_dataminer", pass="pass", db="prod_internal_invitrodb_v3_5", host="ccte-mysql-res.epa.gov")
  v3.5_chid <- dbGetQuery(con, "SELECT * FROM invitrodb.mc5_chid;")
  zdat <- zdat[m5id %in% v3.5_chid$m5id,]
  v4.0_spids <- as.data.table(unique(zdat$spid))
  
  #cat("9: Calculating intermediate summary statistics\n")
  # prior to version 4.0 modl_ga was used as ac50 variable
  # check schema and if using new schema use ac50 instead.

  zdst <- zdat[, list(med = median(eval(modl_ga[hitc==1])),
                        mad = mad(eval(modl_ga[hitc==1])),
                        ntst = .N,
                        nhit = lw(hitc ==1),
                        burstpct = (lw(hitc==1)/.N)), # added burst percent as condition instead of number of hits
                 by = list(chid,code, chnm, casn)]

  #cat("10: Calculating the cytotoxicity point based on the 'burst' endpoints\n")
  zdst[, `:=`(used_in_global_mad_calc, burstpct > 0.05 & ntst>=60)] # updated to 5% from nhit > 1 and originally ntst= length(ae) for tested in all 88 burst assays tested
  gb_mad <- median(zdst[used_in_global_mad_calc=='TRUE', mad])  #calculate global mad
  zdst[,global_mad := gb_mad] # add column for global mad
  zdst[, cyto_pt := med]
  zdst[burstpct < 0.05, `:=`(cyto_pt, .05)] # if the burst percent is less than .05 use the default pt instead
  zdst[, `:=`(cyto_pt_um, 10^cyto_pt)]
  zdst[, `:=`(cyto_pt_log10_um, log10(cyto_pt_um))]
  zdst[, `:=`(lower_bnd_um, 10^(cyto_pt - 3 * global_mad))]
  zdst[, `:=`(lower_bnd_log10_um, log10(lower_bnd_um))]
  zdst[,burstpct:=NULL] # remove burstpct from final results to match previous iterations of tcplCytopt

  zdst3.5 <- zdst[, version:= "v3.5"]
```

```
#incorporate code from from https://github.com/USEPA/CompTox-ToxCast-tcpl/blob/21-tcplcytopt-update/R/tcplCytoPt.R
  #cat("7: Filtering chemical data (if necessary)\n")
  zdat <- v4.0_mc5[!is.na(chid),]
  #filter out gnls curves
  zdat <- zdat[modl != "gnls",]
  
  #for bidirectional burst endpoints only consider down response (BSK and APR aeids below)
  zdat <- zdat %>% filter(!(aeid %in% c(26, 46, 158, 160, 178, 198, 222, 226, 252, 254, 270, 292, 316, 318, 2873, 2929, 2931) & top>0))
  
  #cat("8: Determining representative sample\n") ##not available yet?
  zdat <- zdat[spid %in% v4.0_spids$V1,]

  #cat("9: Calculating intermediate summary statistics\n")
  # prior to version 4.0 modl_ga was used as ac50 variable
  # check schema and if using new schema use ac50 instead.
  
  zdst <- zdat[, list(med = median(eval(log10(ac50)[hitc >= .9])),
                        mad = mad(eval(log10(ac50)[hitc >= .9])), 
                        ntst = .N, 
                        nhit = lw(hitc >= .9), 
                        burstpct = (lw(hitc>=.9)/.N)), # added burst percent as condition instead of number of hits
                 by = list(chid,code, chnm, casn)]
  
  #cat("10: Calculating the cytotoxicity point based on the 'burst' endpoints\n")
  zdst[, `:=`(used_in_global_mad_calc, burstpct > 0.05 & ntst>10)] # updated to 5% from nhit > 1 and ntst= all 73 burst assays
  gb_mad <- median(zdst[used_in_global_mad_calc=='TRUE', mad])  #calculate global mad
  zdst[,global_mad := gb_mad] # add column for global mad
  zdst[, cyto_pt := med]
  zdst[burstpct < 0.05, `:=`(cyto_pt, .05)] # if the burst percent is less than .05 use the default pt instead
  zdst[, `:=`(cyto_pt_um, 10^cyto_pt)]
  zdst[, `:=`(cyto_pt_log10_um, log10(cyto_pt_um))]
  zdst[, `:=`(lower_bnd_um, 10^(cyto_pt - 3 * global_mad))]
  zdst[, `:=`(lower_bnd_log10_um, log10(lower_bnd_um))]
  zdst[,burstpct:=NULL] # remove burstpct from final results to match previous iterations of tcplCytopt

  zdst4.0 <- zdst[, version:= "v4.0"]
```

In v4.0, a slightly higher global MAD was calculated at 0.1629086
compared to 0.1590979 in v3.5. Note that these changes to tcplCytoPt()
were implemented subsequent to creation of the invitrodb v4.0 snapshot,
and as such, the cytotox table in invitrodb v4.0 matches invitrodb v3.5,
and should be considered deprecated. Invitrodb v4.1 will include an
updated cytotox table.

The median of the cytotoxicity AC50 median values is equivalent
between versions at log10-µM, and the median lower bound cytotoxicity
value are extremely similar between datasets (-0.427 and -0.439
log10-µM).

We can evaluate a linear regression model of median cytotoxicity
points between versions:

```
cytotox.model <- lm(v4_cyto_pt_log10_um ~ v3.5_cyto_pt_log10_um, data = zdst.combined2)
summary(cytotox.model)
```

```
## 
## Call:
## lm(formula = v4_cyto_pt_log10_um ~ v3.5_cyto_pt_log10_um, data = zdst.combined2)
## 
## Residuals:
##     Min      1Q  Median      3Q     Max 
## -4.3611 -0.0101 -0.0101  0.0959  1.9039 
## 
## Coefficients:
##                       Estimate Std. Error t value Pr(>|t|)    
## (Intercept)           0.017248   0.004049    4.26 2.06e-05 ***
## v3.5_cyto_pt_log10_um 0.856107   0.004652  184.03  < 2e-16 ***
## ---
## Signif. codes:  0 '***' 0.001 '**' 0.01 '*' 0.05 '.' 0.1 ' ' 1
## 
## Residual standard error: 0.3052 on 8883 degrees of freedom
## Multiple R-squared:  0.7922, Adjusted R-squared:  0.7922 
## F-statistic: 3.387e+04 on 1 and 8883 DF,  p-value: < 2.2e-16
```

The RMSE value is 0.3051228 log10-uM.

```
suppfig6b <- ggplot(data=zdst.combined2) +
  geom_point(aes(x=v4_cyto_pt_log10_um, y=v3.5_cyto_pt_log10_um),size=2, alpha=0.2)+ 
  xlab("v4.0 Log10 Burst Median (uM)")+
  ylab("v3.5 Log10 Burst Median (uM)")+
  xlim(-3, 3) +
  ylim(-3,3) +
  # add reference lines
  geom_abline(slope = 1,intercept = 0,col = "gray")+
  geom_abline(slope = 1,intercept = c(-0.5,0.5),
              col = "purple",linetype = "dotted")+
  geom_abline(slope = 1,intercept = c(-2.5,2.5),
              col = "blue",linetype = "dashed")+
  theme_bw() +
  theme(axis.title.x = element_text(face='bold', size=14),
        axis.title.y = element_text(face='bold', size=14),
        axis.text.x = element_text(size=12),
        axis.text.y = element_text(size=12))+
  annotate('text', x=-2, y=2, face = 'bold', size = 5, label='Adj R2 = 0.7922')
```

```
suppfig6 <- plot_grid(suppfig6a, suppfig6b, labels = c("A", "B"), label_size = 16, ncol=2, rel_widths = c(1,.75),
          rel_heights = c(1,1))
suppfig6
```

```
file.dir <- paste(getwd(), sep="")
file.name <- paste("/Supp_Fig6_burst_", Sys.Date(), ".png", sep="")
file.path <- paste(file.dir, file.name, sep="")
dir.create(path=file.dir, showWarnings = FALSE, recursive = TRUE)
png(width=6000, height=4000, res=600)
suppfig6
dev.off()
```
