# Supplementary material for: The ToxCast pipeline: updates to curve-fitting approaches and database structure
Source: Front Toxicol. 2023 Sep 21;5:1275980. doi: 10.3389/ftox.2023.1275980 (PMC10552852; doi:10.3389/ftox.2023.1275980)

The ToxCast Pipeline: Updates to Curve-fitting Approaches and Database Structure

Feshuk M^1^, Kolaczkowski L^1,2^, Dunham K^1,2^, Davidson-Fritz S^1^, Carstens KE^1^, Brown J^1^, Judson RS^1^, Paul Friedman K^1^

# Supplemental Figures

## Supplemental Figure 1. Invitrodb v4.0 general schema

A generic representation of the ToxCast database, invitrodb, schema for invitrodb v4.0. Highlighted in gray are the new tables (when compared to invitrodb v3.5) or tables impacted by tcpl v3.0 updates.


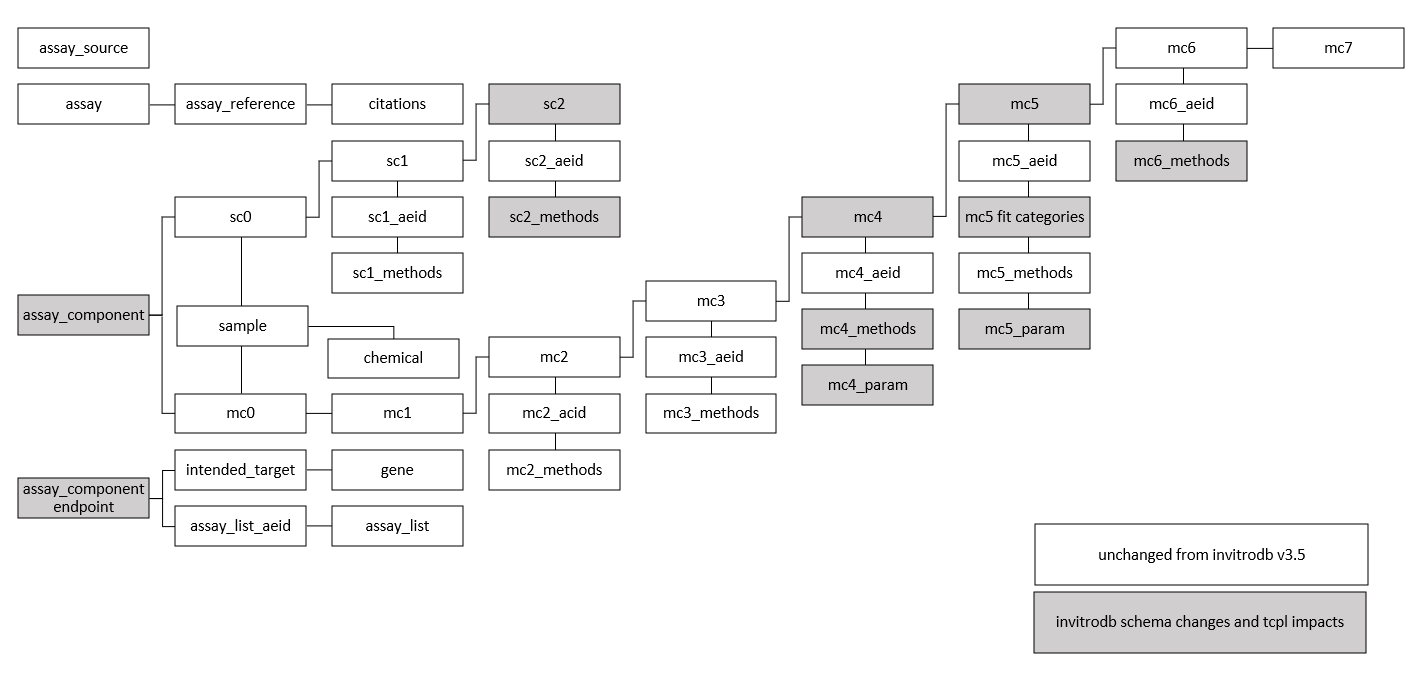


## Supplemental Figure 2. Frequency distribution of invitrodb v4.0 continuous hitcall

The continuous hitcall is the product of three proportional weights and is not normally distributed, as visualized here with the frequency of curve-fits versus the continuous hitcall, 0 to 1. Most values fall between 0 and 0.1 and 0.9 and 1.


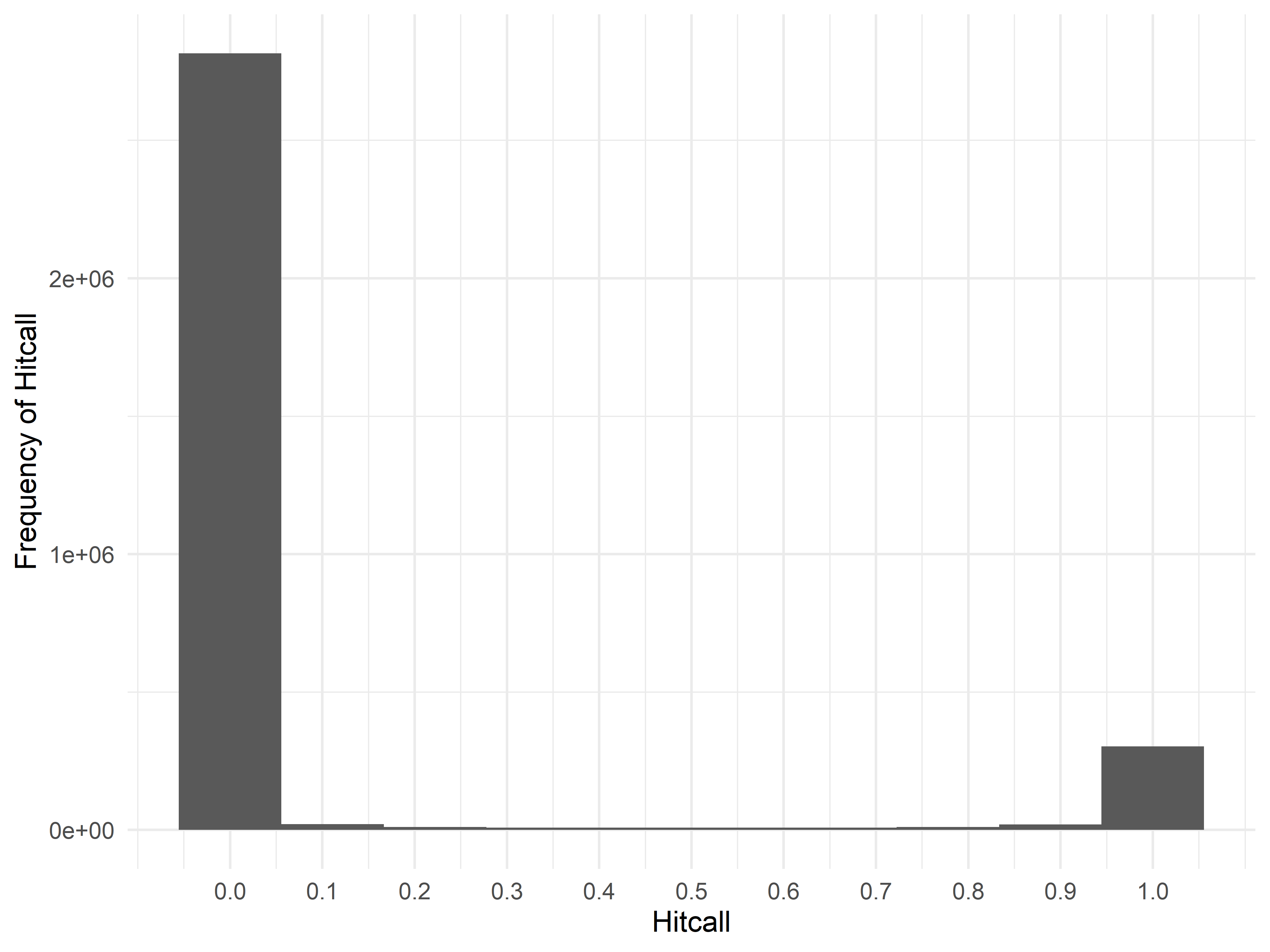


## Supplemental Figure 3. Cutoff versus top analysis

The kernel density plot showing the top/cutoff fold difference (‘top_over_cutoff’ in invitrodb v4.0) for AA, AI, IA flip directions. The vertical lines indicate 1 or 1.5 top/cutoff fold difference. AA = active to active; AI = active to inactive; IA = inactive to active; all flips are from invitrodb v3.5 to invitrodb v4.0.


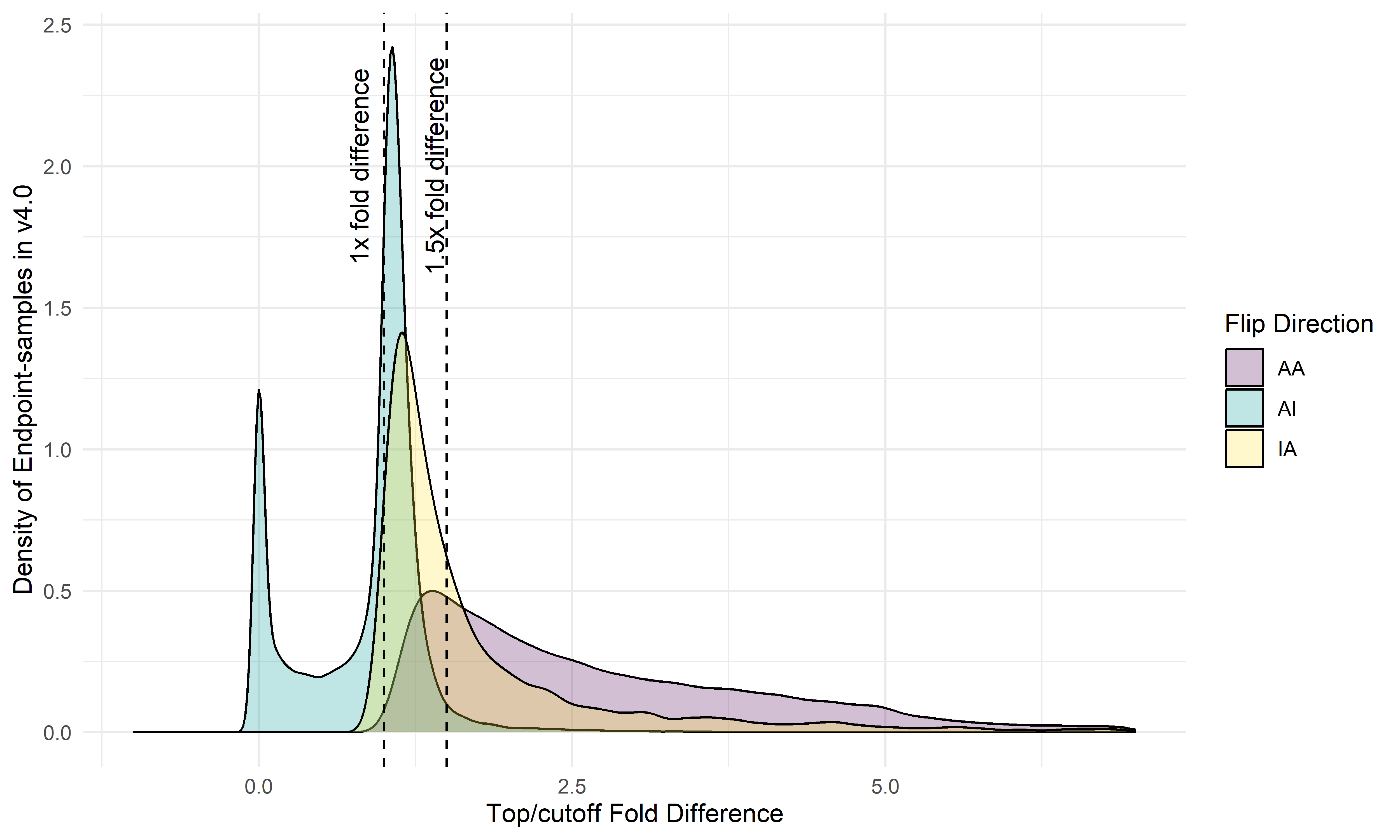


## Supplemental Figure 4. Potency Estimate Shifts.

In A, comparisons of the potency metrics from invitrodb v3.5 (white) to invitrodb v4.0 (gray) are illustrated, noting that BMD values are new to invitrodb v4.0. In B, the flip direction (AA or active to active; AI or active to inactive) and its impacts on this comparison of potency metrics between invitrodb v3.5 and v4.0 are illustrated.


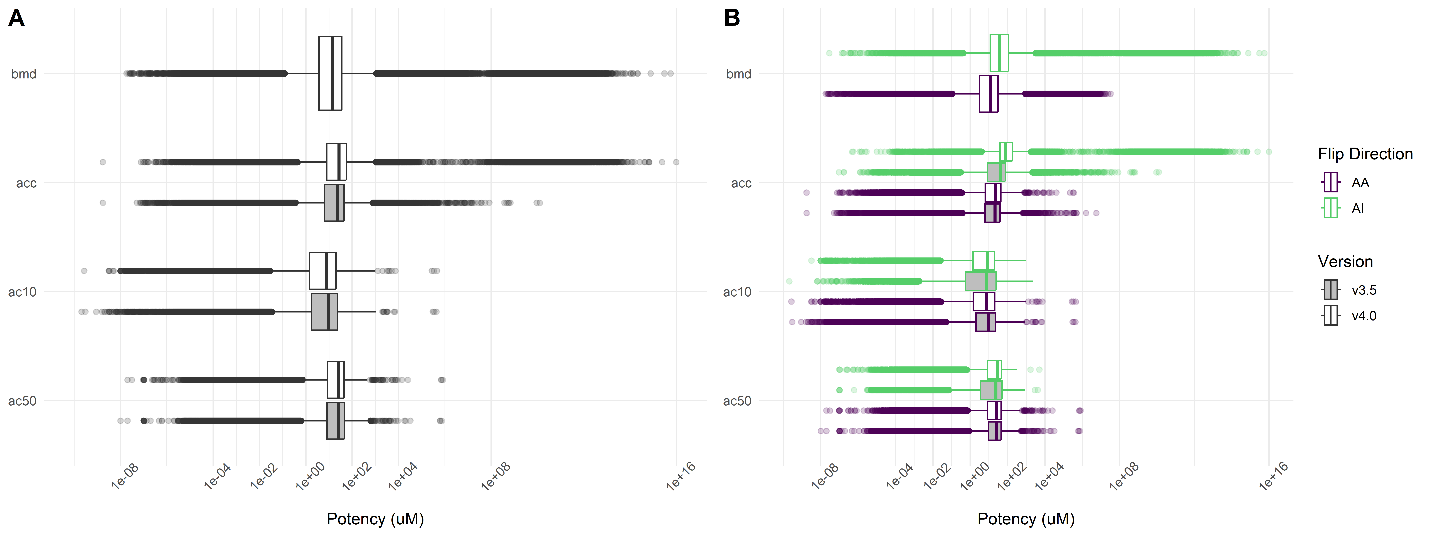


## Supplemental Figure 5. Potency Estimate Shifts by Assay Source

In A, boxplots of AC50 by assay source are compared between invitrodb v3.5 and invitrodb v4.0. In B, boxplots of ACC by assay source are compared between invitrodb v3.5 and invitrodb v4.0. The differences are generally minor.


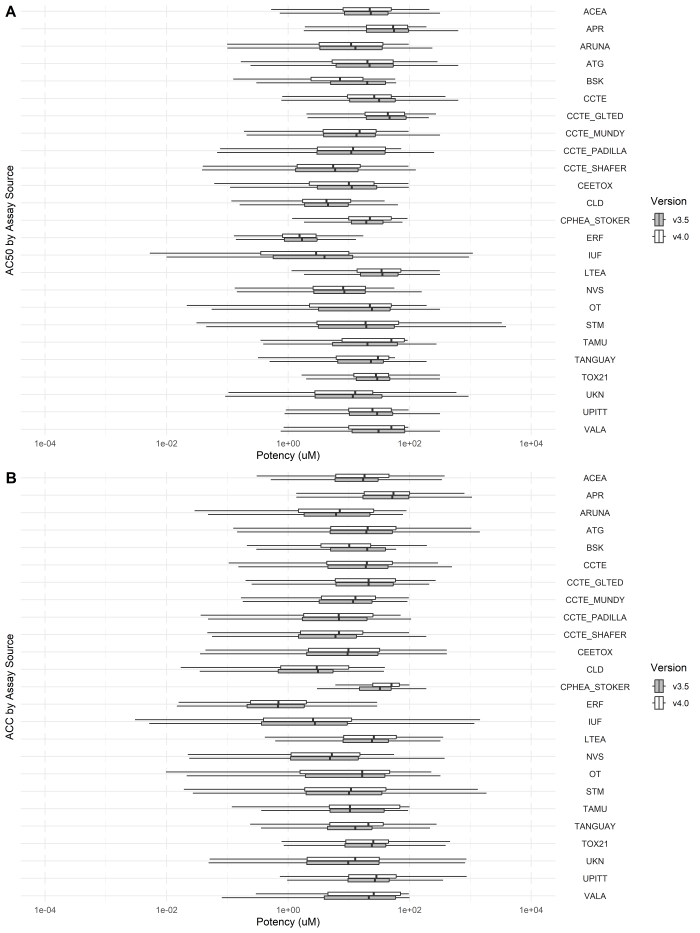


## Supplemental Figure 6. Cutoff versus benchmark response.

In A, the log10(cutoff) – log10(BMR) frequency suggests that most cutoff values are greater than BMR values, often by 0.5. In B, a linear relationship between Cutoff and BMR approached an adjusted coefficient of variation of 0.7, with most BMR values within ± 0.5 log10 of the corresponding cutoff value for the same assay endpoint.


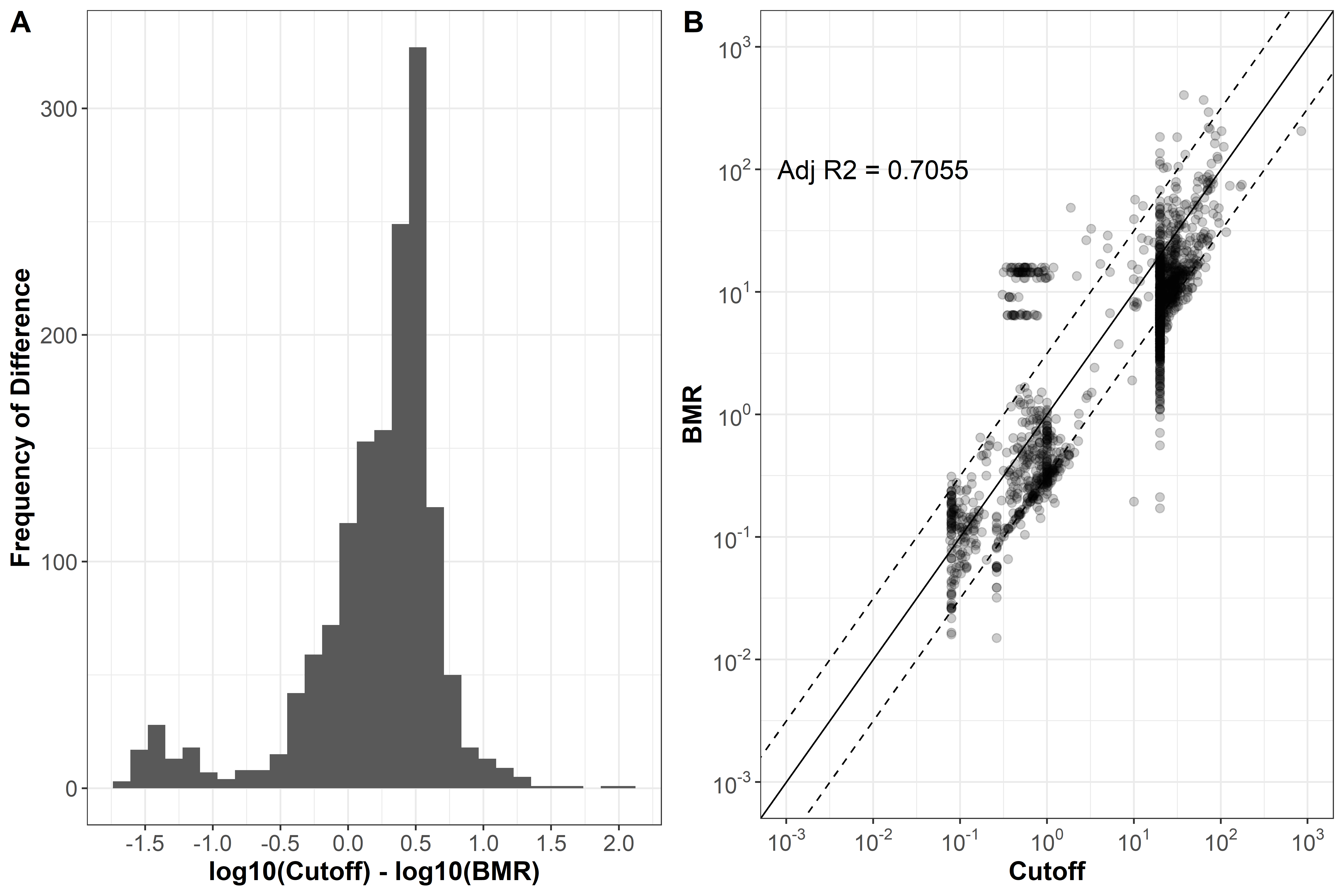


## Supplemental Figure 7: Cytotoxicity Threshold Change

In A, density plots of the log10-µM median and lower bound on the median estimate of the cytotoxicity burst are shown. The density plots for invitrodb v3.5 and invitrodb v4.0 overlap. In B, a linear relationship is evident between the cytotoxicity burst median values between invitrodb v3.5 and invitrodb v4.0. The cytotoxicity burst median values for most chemicals change <|0.5 log10-µM|, as indicated by the dashed lines.


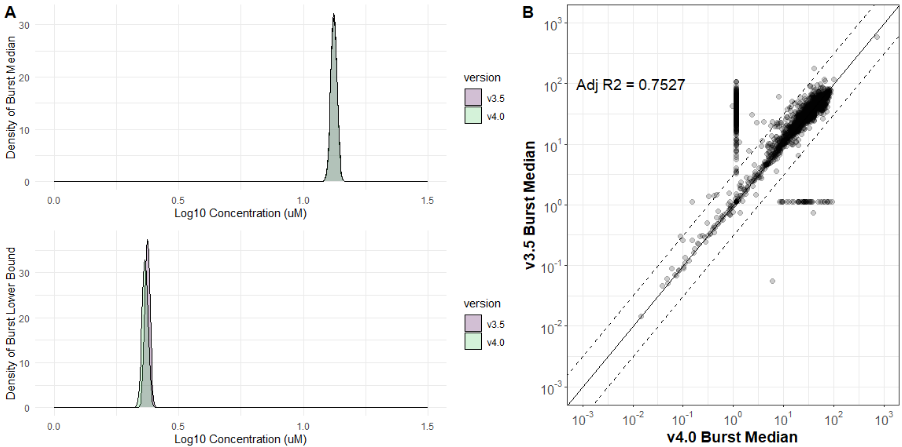

Supplement: Supplementary file 2 [file DataSheet1.docx]
